# Discovery of *N*-quinazolinone-4-hydroxy-2-quinolone-3-carboxamides as DNA gyrase B-targeted antibacterial agents

**DOI:** 10.1080/14756366.2022.2084088

**Published:** 2022-06-07

**Authors:** Wenjie Xue, Yaling Wang, Xu Lian, Xueyao Li, Jing Pang, Johannes Kirchmair, Kebin Wu, Zunsheng Han, Xuefu You, Hongmin Zhang, Jie Xia, Song Wu

**Affiliations:** a State Key Laboratory of Bioactive Substance and Function of Natural Medicines, Department of New Drug Research and Development, Institute of Materia Medica, Chinese Academy of Medical Sciences and Peking Union Medical College, Beijing, China; b Department of Pharmacy, Shanxi Bethune Hospital, Shanxi Academy of Medical Sciences, Tongji Shanxi Hospital, Third Hospital of Shanxi Medical University, Taiyuan, China; c School of Pharmacy, Jiangsu Ocean University, Lianyungang, China; d Institute of Medicinal Biotechnology, Chinese Academy of Medical Sciences and Peking Union Medical College, Beijing, China; e Division of Pharmaceutical Chemistry, Department of Pharmaceutical Sciences, University of Vienna, Vienna, Austria; f Department of Biology, Guangdong Provincial Key Laboratory of Cell Microenvironment and Disease Research, Shenzhen Key Laboratory of Cell Microenvironment and SUSTech-HKU Joint Laboratories for Matrix Biology, Southern University of Science and Technology, Shenzhen, China

**Keywords:** Antibiotic resistance, MRSA, antibacterial agent, DNA Gyrase inhibitors, computer-aided drug design

## Abstract

Emerging drug resistance is generating an urgent need for novel and effective antibiotics. A promising target that has not yet been addressed by approved antibiotics is the bacterial DNA gyrase subunit B (GyrB), and GyrB inhibitors could be effective against drug-resistant bacteria, such as methicillin-resistant *S. aureus* (MRSA). Here, we used the 4-hydroxy-2-quinolone fragment to search the Specs database of purchasable compounds for potential inhibitors of GyrB and identified **AG-690/11765367,** or **f1**, as a novel and potent inhibitor of the target protein (IC_50_: 1.21 µM). Structural modification was used to further identify two more potent GyrB inhibitors: **f4** (IC_50_: 0.31 µM) and **f14** (IC_50_: 0.28 µM). Additional experiments indicated that compound **f1** is more potent than the others in terms of antibacterial activity against MRSA (MICs: 4–8 µg/mL), non-toxic to HUVEC and HepG2 (CC_50_: approximately 50 µM), and metabolically stable (t_1/2_: > 372.8 min for plasma; 24.5 min for liver microsomes). In summary, this study showed that the discovered N-quinazolinone-4-hydroxy-2-quinolone-3-carboxamides are novel GyrB-targeted antibacterial agents; compound **f1** is promising for further development.

## 1. Introduction

Antibiotic resistance poses a significant threat to global public health. The number of individuals who die from infections caused by antibiotic-resistant bacteria is projected to rise to 10 million by 2050.^1^ In response to antibiotic resistance, the WHO published a priority list of pathogens in 2017, for which new antibiotics are urgently needed. In the category of Gram-positive bacteria, methicillin-resistant *S. aureus* (MRSA) was designated as “high-priority status”.^2^ The antibiotics currently available to treat infectious diseases caused by MRSA are vancomycin, daptomycin, and linezolid. Unfortunately, their use has been limited in clinical practice. The first concern is their safety, e.g. the nephrotoxicity of vancomycin, the unknown proper dose of daptomycin, as well as the potential risk of thrombocytopenia caused by the high plasma levels of linezolid.^3^ The second is the antibiotic resistance, which makes them lose antibacterial activity; strains of resistant *S. aureus* have been isolated in the clinic.^4^ Emerging antibacterial compounds in late stage clinical trials often have favourable toxicity profiles, but they are still in the same class as existing drugs and thus may still fail to treat resistant strains.^5–7^ Therefore, new classes of antibacterial agents for MRSA are urgently needed.

**Figure 1.**
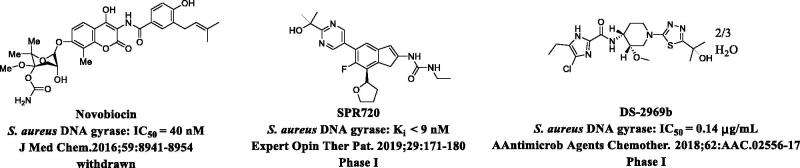
Overview of representative bacterial DNA Gyrase B inhibitors.

Bacterial DNA gyrase B subunit (GyrB) is a promising target for discovery and development of a new class of antibiotics.^8^ As an indispensable component of DNA gyrase (A_2_B_2_), GyrB binds ATP at the ATPase domain and catalyses ATP hydrolysis; it provides energy for DNA supercoiling.^9^ When the GyrB inhibitor novobiocin was approved for clinical use (cf. Figure 1), antibiotics with the same mode of action were considered as promising therapeutics for the treatment of bacterial infections.^7^ Since the decline of novobiocin in 1960s due to its toxicity and low efficacy, several diverse GyrB inhibitors have been discovered, e.g. ethyl ureas,^10^ pyrazolopyridones,^11^ pyrrolamides,^12^ and quercetin diacylglycosides.^13^ Unfortunately, none of these have been approved. Two compounds, i.e. SPR720 (ethyl ureas)^14^ and DS-2969b (pyrrolamides),^15^ are in phase I clinical trials (cf. Figure 1), but the clinical outcomes of these chemotypes are also unpredictable. Therefore, the identification of diverse structures as GyrB inhibitors is still necessary.

Here, we used the chemical information of the N-thiadiazole-4-hydroxy-2-quinolone-3-carboxamides to identify new GyrB inhibitors. With the essential fragment for GyrB inhibition as the substructure, we searched the Specs database of purchasable compounds for related substances. By testing the compounds in *S. aureus* GyrB inhibition assay, we discovered that the 4-hydroxy-2-quinolone-3-carboxamide derivative that has an N-quinazolinone moiety inhibits GyrB. To understand the preliminary structure–activity relationship (SAR), we synthesised derivatives and evaluated their activities against GyrB. Representative GyrB inhibitors were submitted for *in vitro* evaluation of the antibacterial activity against a panel of *S. aureus* strains. Finally, we studied the cytotoxicity, ADMET profile, and important physicochemical properties of the most active antibacterial agent.

## 2. Results and discussions

### 2.1. Computer-aided hit identification

**Figure 2.**
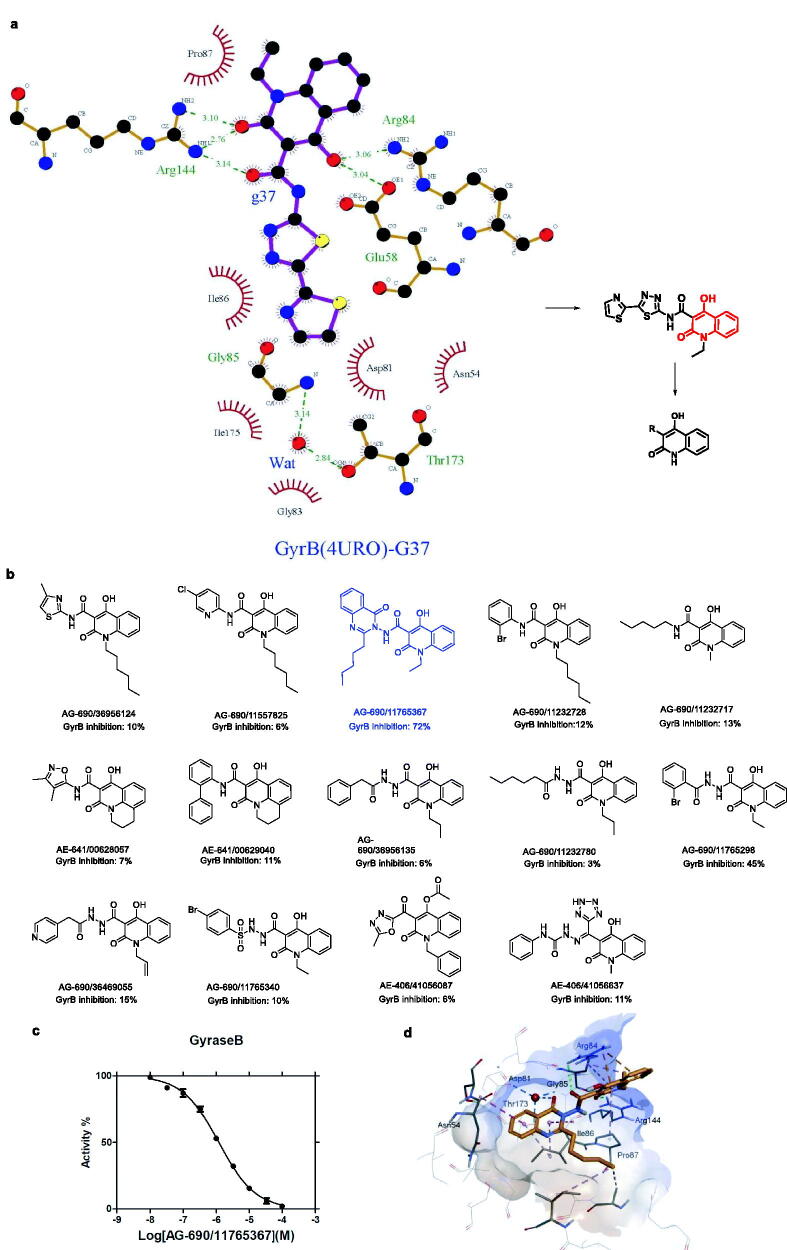
Computer-guided search for novel GyrB inhibitors. (a) Essential fragment for the binding of **g37** to the ATPase domain of *S. aureus* GyrB. The image was generated with LigPlot + in which hydrogen bonds and names of the interacting residues are coloured in green. (b) Structures selected from the Specs compound library and their GyrB inhibition rates (%) at 10 μM. Novobiocin was set as the positive control (inhibition rate at 10 μM: 99%). (c) Concentration-dependent ATPase inhibition of *S. aureus* Gyrase B (GyrB). The calculated IC_50_ value of **AG-690/11765367** was 1.21 μM. Novobiocin was set as the positive control (IC_50_: 0.02 μM). (d) Likely binding mode of the hit compound **AG-690/11765367** to the ATP binding site of *S. aureus* GyrB as derived by molecular docking. The interacting residues and the hit compound are shown in stick representations; the binding site is shown as a surface representation.

We previously^16^ proposed the likely binding mode of the N-thiadiazole-4-hydroxy-2-quinolone-3-carboxamides bearing heteroaromatic rings to the ATP binding site of *S. aureus* GyrB by molecular docking. Here, we utilised LigPlot+^17^ to generate a 2D diagram of the representative GyrB inhibitor **g37**. Figure 2(a) clearly shows that the 4-hydroxy-2-quinolone fragment plays the most important role in the binding to GyrB: its carbanyl group forms hydrogen bonds with Arg144, and its 4-hydroxyl group is involved in the formation of hydrogen bonds with Glu58 and Arg84. We performed a substructure search of the Specs database with the 4-hydroxy-2-quinolone fragment and identified 272 matches (in approximately 210,000 compounds). Aided by a clustering analysis and visual inspection of the molecular structures, we selected 14 potential GyrB inhibitors for experimental validation. Figure 2(b) shows that all compounds except for **AE-406/41056087** and **AE-406/41056637** are based on the chemotype of 4-hydroxy-2-quinolone-3-carboxamides and have diverse substituents attached to the amide nitrogen atom, i.e. thiazole, pyridine, 4-oxoquinazolin, phenyl, alkyl, oxazole, biphenyl, phenacetylamino, alkyl amide, benzamide, pyridine acetamide, and benzsulfamide.

In the *S. aureus* GyrB assay, **AG-690/11765367** (also named as **f1**) reduced the activity of *S. aureus* GyrB by 72% at a concentration of 10 μM (cf. Figure 2(b)). The IC_50_ value was determined to be 1.21 ± 0.13 μM based on the dose-response curve (cf. Figure 2(c)). Interestingly, it contained a 4-oxoquinazolin fragment that was different from the previously reported thiadiazole. The identification of such a novel GyrB inhibitor confirms the capacity of the computer-aided strategy. Notably, the other 13 compounds could not inhibit *S. aureus* GyrB by 50% at 10 μM (cf. Figure 2(b)), thus indicating that the type of substituents at position N of the amide is essential to GyrB inhibition. We performed molecular docking with OEDocking version 3.0.1 (OpenEye Scientific Software, Inc., Santa Fe, NM)^18^ to derive a plausible binding mode of **AG-690/11765367** to the ATP binding site of GyrB. In general, the binding mode of **AG-690/11765367** is predicted to be similar to that of compound **g37**.^16^ More specifically, the 4-hydroxy-2-quinolone-3-carboxamide is expected to form hydrogen bonds with Arg84 and Arg144, electrostatic interactions with Arg84, and hydrophobic interactions with Pro87. The 4-oxoquinazolin fragment is predicted to be located in a sub-pocket surrounded by the hydrophobic residues including Asn54 and Ile86. The carbonyl group of the 4-oxoquinazolin fragment is expected to be uniquely involved in water-mediated interactions with Thr173, Asp81, and Gly85 (cf. Figure 2(d)).

### 2.2. Molecular design and chemical synthesis

**Scheme 1.**
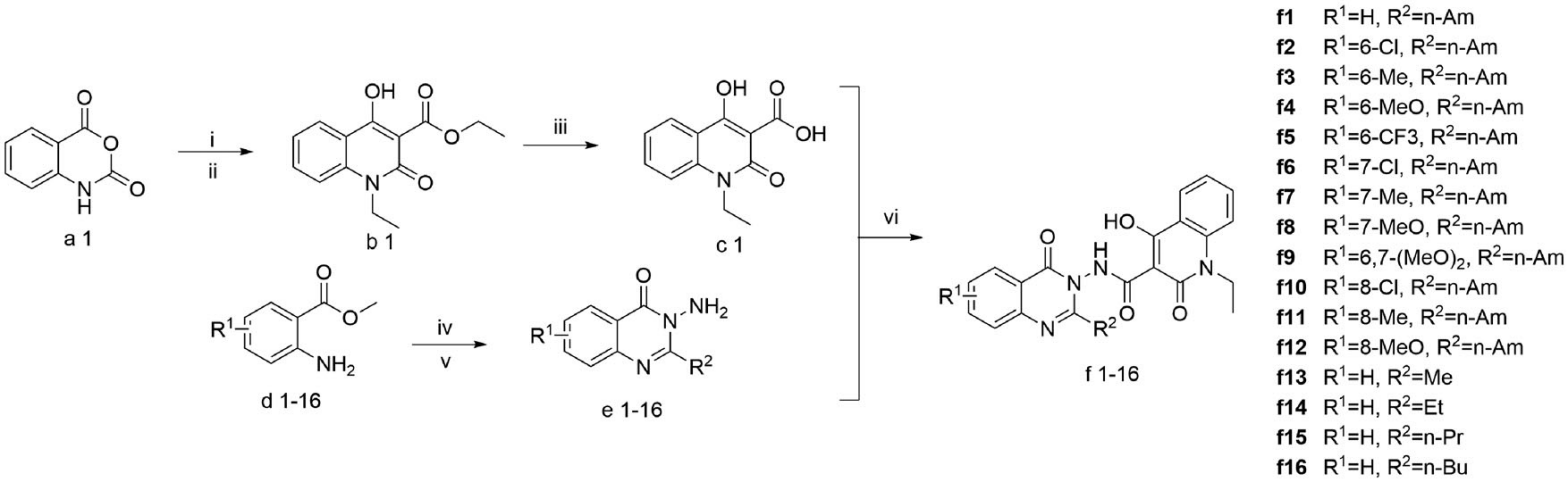
Reagents and conditions: (i) DIPEA, 45 °C, DMF, CH_3_CH_2_I, 10 h; (ii) NaH, DMF, diethyl malonate, 70 °C, 8 h; (iii) 12 N HCl, MeOH, 65 °C, 10 h; (iv) R^2^COCl, TEA, DCM, r.t.; (v) hydrazine hydrate, EtOH, 78 °C, 10 h; and (vi) HATU, DIPEA, DMF, r.t, 48 h.

To preliminarily study the SAR of **f1**, we designed two series of derivatives by (i) introducing the electron-donating groups or electron-withdrawing substituents to the benzene ring of the newly identified 4-oxoquinazolin fragment (R^1^ in Scheme 1, **f2–f12**) and (ii) replacing the pentyl group with other alkyl groups at position 2 of the 4-oxoquinazolin fragment (R^2^ in Scheme 1, **f13–f16**).

The synthetic route of the above-mentioned derivatives was planned as the condensation reactions between the 4-hydroxy-2-oxo-1,2-dihydroquinoline-3-carboxylic acid and different substituted 3-aminoquinazolin-4(3*H*)-ones according to published methods.^19^

In practice, the synthesis of the key intermediate **c1**, namely 1-ethyl-4-hydroxy-2-quinolone-3-carboxylic acid, was composed of three consecutive steps (cf. Scheme 1): first, isatoic anhydride was ethylated in the presence of *N*,*N*-diisopropylethylamine and iodoethane. This reaction introduced the ethyl group to the heterocyclic nitrogen of isatoic anhydride. Second, the ethylated anhydride was treated with diethyl malonate and sodium hydride to afford the intermediate **b1**. Third, **b1** was converted to the acid by hydrolysis under the condition of 12N hydrochloric acid and the refluxing methanol. The key amine intermediates **e1–e16** were prepared by conversion of different substituted methyl 2-aminobenzoates **d1–d16** into amides under mild condition and with triethylamine as base, followed by annulation with hydrazine hydrate in boiling ethanol. The target molecules **f1–f16** were obtained by coupling the above-mentioned acid **c1** with the corresponding amines (**e1–e16**). All synthesised compounds were characterised by melting points, ^1^H NMR, ^13^C NMR, and HRMS. The details of the chemical synthesis and structural characterisation are described in the experimental section.

The ^1^H NMR spectra of **f1-f16** revealed the appearance of a methylene signal from 4.46 to 4.07 (–CH_2_–N) and a methyl signal from 1.20 to 1.44 (CH_3_–CH_2_–N). The singlet at 12.62–12.31 ppm represented the NH proton of the 3-carboxamide. For **f1**–**f12** and **f15**–**f16**, the methylene proton signals of the quinazolinone side chain were observed at 2.98–2.56 ppm (–CH_2_–C = N), 1.90–1.71 ppm (–CH_2_–CH_2_–C = N), and 1.44–1.20 ppm (– (CH_2_)_2_–CH_2_–CH_2_–C = N); the methyl proton signal appeared at 0.93–0.83 ppm (CH_3_(CH_2_)_n_-C = N). For **f13** and **f14**, the methyl protons were shown as the signals at 2.55 ppm and 1.34–1.28 ppm, respectively. The aromatic C–H protons of **f1**–**f16** were displayed in ^1^H NMR spectra as signals at 8.56–7.06 ppm. The ^13 ^C NMR spectra of **f1**–**f16** showed the characteristic ethyl carbon (N–C–C) at 37.61–33.92 ppm and 14.36–13.26 ppm as well as the carbonyl carbon (C = O) at 172.02–157.56 ppm.

### 2.3. Inhibitory activity against S. aureus Gyrase B

The inhibitory activities of all the derivatives for *S. aureus* GyrB in terms of IC_50_ are reported in Table 1. In general, the IC_50_ values of most derivatives were close to that of compound **f1** (1.21 μM). Among the derivatives with R^1^ substituents at different positions of the benzene ring, the most potent one was compound **f4** with a 6-methoxyl substituent (IC_50_: 0.31 μM); the weakest was compound **f7** with a 7-methyl group (24.40 μM). Other potent compounds include **f3** (0.83 μM), **f5** (0.81 μM), **f6** (0.83 μM), **f11** (0.77 μM), and **f12** (0.88 μM). The introduction of electron-donating groups to position 6/8 or electron-withdrawing groups to position 7 is favourable for GyrB inhibition. For instance, **f4** (6-MeO, 0.31 μM) showed greater inhibition against GyrB than **f6** (6-CF_3_, 0.81 μM). Compound **f12** (8-MeO, 0.878 μM) was more potent than compound **f10** (8-Cl, 9.70 μM). In contrast, **f8** substituted by a 7-methoxyl group (7.90 μM) was much less potent than **f6** with a 7-chlorine moiety (0.83 μM). Derivatives with different alkyl substituents at position 2 of the 4-oxoquinazolin fragment (compounds **f13**–**f16**) have IC_50_ values between 0.28 and 11.9 μM (cf, Table 1). These values are close to that of compound **f1 (**1.21 μM**)**, and the replacement of the pentyl group with other alkyl groups only led to a small change in GyrB inhibition.

**Table 1.** Chemical structures and GyrB inhibitory activity of compound **f1** and its derivatives (**f2–f16**).

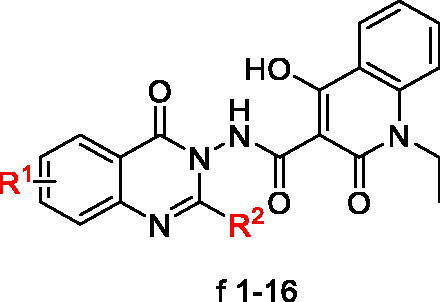			
Compound ID	R^1^	R^2^	IC_50_ for GyrB (µM, mean ± SD)^a^
**f1**	H	n-Am	1.21 ± 0.13
**f2**	6-Cl	n-Am	1.58 ± 0.06
**f3**	6-Me	n-Am	0.83 ± 0.11
**f4**	6-MeO	n-Am	0.31 ± 0.07
**f5**	6-CF_3_	n-Am	0.81 ± 0.17
**f6**	7-Cl	n-Am	0.83 ± 0.01
**f7**	7-Me	n-Am	24.40 ± 4.50
**f8**	7-MeO	n-Am	7.90 ± 0.10
**f9**	6,7-(MeO)_2_	n-Am	1.06 ± 0.13
**f10**	8-Cl	n-Am	9.70 ± 2.10
**f11**	8-Me	n-Am	0.77 ± 0.02
**f12**	8-MeO	n-Am	0.88 ± 0.15
**f13**	H	Me	1.64 ± 0.23
**f14**	H	Et	0.28 ± 0.04
**f15**	H	n-Pr	11.90 ± 2.30
**f16**	H	n-Bu	2.34 ± 0.34

^a^Mean: average of duplicates; SD: standard deviation

In this assay, novobiocin was set as the positive control (IC_50_: 0.02 µM).

**Figure 3.**
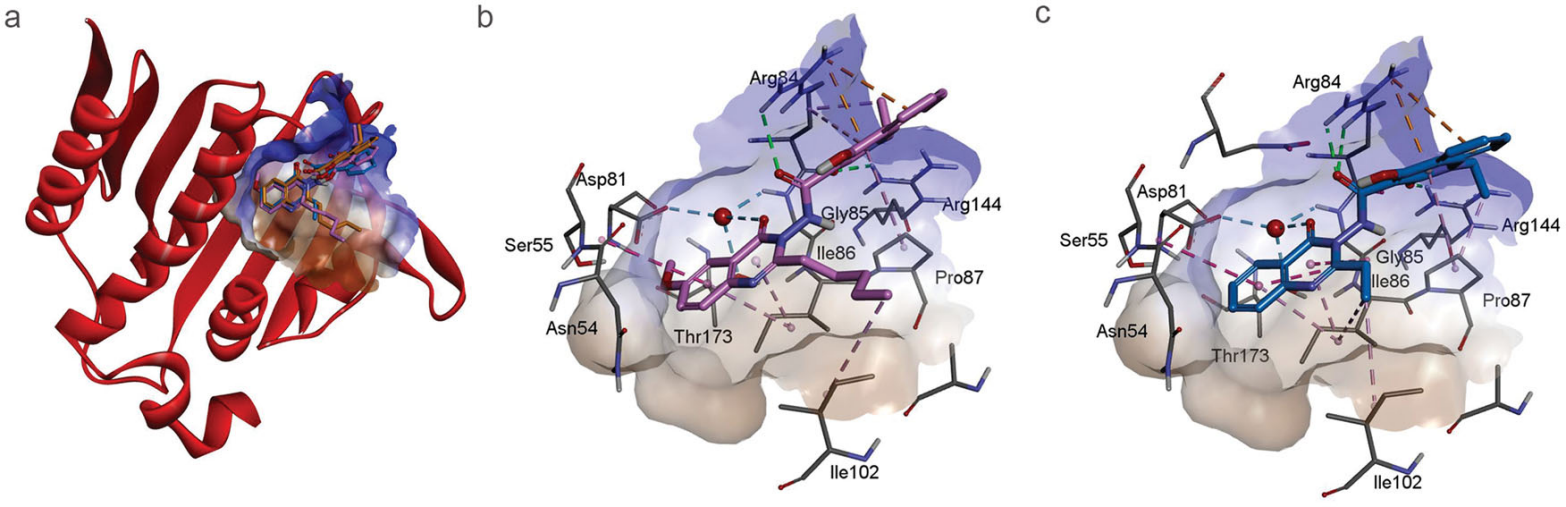
Predicted binding modes of **f4** and **f14** to the ATP binding site of *S. aureus* GyrB as derived from molecular docking experiments. (a) Compounds **f4** and **f14** superimposed with **f1**. (b) Interactions formed between **f4** and *S. aureus* GyrB. (c) Interactions formed between **f14** and *S. aureus* GyrB. The interacting residues and the ligands are shown in stick representation. Colour code: pink, compound **f4**; blue, compound **f14**.

The most potent derivatives in two series, i.e. **f4** and **f14**, were docked against the ATPase domain of GyrB. Their predicted binding modes are shown in Figure 3. As both **f4** and **f14** are based on a 4-hydroxy-2-quinolone-3-carboxamide scaffold and the 4-oxoquinazolin fragment, the predicted binding poses were similar to that of **f1**. First, they are predicted to bind to the same position of the ATP binding site and are superimposed well with the predicted binding mode of **f1** (cf. Figure 3(a)). Second, the predicted key interactions are identical including the hydrogen bonds with Arg84 and Arg144, electrostatic interactions with Arg84, hydrophobic interactions with Pro87, and water-mediated interactions with Thr173, Asp81, and Gly85. Uniquely, **f4**, with a methoxy substituent at position 6, seems to better occupy the hydrophobic region defined by Asn54, Ser55, and Thr173 (cf. Figure 3(b)). The pentyl group of compound **f1** is outside the pocket (cf. Figure 2(d)), while the ethyl group fits the ligand binding pocket well and is positioned close to the edge of the surface (cf. Figure 3(c)).

### 2.4. Antibacterial activity against a panel of S. aureus strains

To identify GyrB inhibitors with potent antibacterial activity, we next tested the hit compound **f1** and the two highly potent derivatives, **f4** and **f14**, against five isolates of methicillin-sensitive *S. aureus* (MSSA; ATCC 29213, 15, 18–3, BAA976, and BAA1708) in a broth microdilution assay.^20^ The MIC (minimal inhibitory concentration) values of **f4** and **f14** were 32 µg/mL, 64 µg/mL, or greater than 64 µg/mL for different isolates (Table 2); the MICs for **f1** ranged from 4 to 8 µg/mL. Compounds **f4** and **f14** showed much weaker activity against *S. aureus* though they inhibited *S. aureus* GyrB more potently. To explain this inconsistency, we calculated the log*P* values of the three compounds with ChemDraw Ultra version 14.0 (Cambridge Scientific Computing, Inc., Cambridge, MA), i.e. 3.13 for compound **f1**, 3.01 for compound **f4**, and 1.88 for compound **f14**. Accordingly, we postulate that high hydrophobicity may favour the antibacterial activity of GyrB inhibitors.

**Table 2.** Antibacterial activity of **f1** and its derivatives, **f4** and **f14**, against a panel of *S. aureus* strains.

Bacterium	Strain	MIC (μg/mL)			
		f1	f4	f14	Vancomycin^b^
MSSA^a^	ATCC 29213	4.0	>64.0	>64.0	1.0
	15	8.0	64.0	64.0	1.0
	18-3	4.0	64.0	>64.0	0.5
	BAA976	4.0	32.0	32.0	0.5
	BAA1708	4.0	>64.0	64.0	1.0
MRSA^a^	ATCC 33591	4.0	n.d.	n.d.	2.0
	ATCC 43300	4.0			1.0
	18-2	8.0			0.5
VISA^a^	ATCC 700699	8.0			4.0
	HIP 5836	16.0			4.0
	HIP 5827	4.0			4.0

^a^MSSA: methicillin-sensitive *S. aureus*; MRSA: methicillin-resistant *S. aureus*; VISA: vancomycin intermediate resistant *S. aureus*

^b^Vancomycin was the positive control drug used in this assay.

The MIC value of compound **f1 (**4–8 µg/mL**)** was close to that of vancomycin (0.5–1 µg/mL), and thus, we further tested **f1** against a panel of MRSA strains (ATCC 33591, ATCC 43300, 18–2) and vancomycin-intermediate-resistant *S. aureus* (VISA) strains (ATCC 700699, HIP 5836, and HIP 5827). Likewise, the antibacterial activities of compound **f1** for MRSA and VISA were similar to those of vancomycin (MICs: 4–16 *vs.* 0.5–4 µg/mL). The data listed in Table 2 demonstrate that the GyrB inhibitor **f1** was the most potent antibacterial agent.

### 2.5. Drug-likeness of compound f1

To see whether **f1** is a promising lead compound for further optimisation, we first investigated its cytotoxicity against human umbilical vein endothelial cells (HUVECs) and human hepatocellular liver carcinoma cells (HepG2) in the sulforhodamine B (SRB) assay.^21^ The CC_50_ values of this compound were 49.6 ± 0.2 µM for HepG2 and 51.5 ± 4.5 µM for HUVECs (Table 3). The MIC values for the tested MRSA/VISA strains were between 4 and 16 µg/mL (i.e. 8.97 and 35.87 µM). These data indicate that compound **f1** is not toxic to mammalian cells at concentrations at which bacterial growth is inhibited.

**Table 3.** Drug-likeness properties measured or predicted for **f1**.

Property		Compound **f1** (**AG-690/11765367**)
*In vitro* cytotoxicity	HepG2	49.6 ± 0.2
CC_50_ (μM, mean ± SD)^a^	HUVEC	51.5 ± 4.5
*In vitro* metabolic stability^b^ (mouse)	In plasma	> 372.8
*t*_1/2_ (min)	In liver microsomes	24.5
*In-silico* predictions	Gastrointestinal (GI) absorption	High
	Blood–brain barrier (BBB) permeability	No
	Lipinski’s Rule-of-Five	No violations
	Water solubility	Moderately or poorly soluble

^a^Cytotoxicity measured after 72-h treatment with **f1**.

Mean: average of duplicates; SD: standard deviation

Paclitaxel was the positive control for the cytotoxicity assay (CC_50_ for HepG2 cells: 12.7 ± 2.1 nM; CC_50_ for HUVECs: 1.7 ± 0.2 nM).

^b^For mouse plasma and microsomal stability assays, propantheline and dextromethorphan were used as the positive control, and their half-life (*t*_1/2_) values were 66.3 and 16.0 min, respectively.

To gain insights into the pharmacokinetic profile of **f1**, the compound was first tested *in vitro* for metabolic stability by incubating it with the mouse plasma and liver microsomes. The data indicate that **f1** is quite stable in mouse plasma, with a half-life (*t*_1/2_) value greater than 372.8 min. Compound **f1** was also metabolically stable in mouse liver microsomes – the *t*_1/2_ value was 24.5 min. These results are consistent with our predictions with FAME 3 – a model for the prediction of sites of metabolism for phase 1 and 2 metabolic enzymes.^22^^,^^23^ FAME 3 flagged only the ethyl side chain of **f1** with a moderate likelihood of being a site of metabolism (cf. Figure S3).

Apart from *in vitro* cytotoxicity and metabolic stability, we used a free web service, SwissADME (http://www.swissadme.ch/),^24^ to predict further key properties related to drug-likeness. Compound **f1** was predicted to have high gastrointestinal (GI) absorption and did not penetrate the brain–blood barrier (BBB). Also, the physicochemical properties do not violate Lipinski’s Rule-of-Five. Nevertheless, aqueous solubility may be an issue that should be addressed. Full details on all predictions are provided in Figure S4.

## 3. Conclusion

*S. aureus* has developed resistance against multiple antibiotics in clinical use including even the recently introduced daptomycin and linezolid.^4^ Thus, antibacterial agents based on novel chemotypes or modes of actions are urgently needed to tackle antibiotic resistance. GyrB is a promising target for compounds breaking the antimicrobial resistance of *S. aureus*. Unfortunately, there is no GyrB inhibitor approved for clinical use, and the outcome of GyrB inhibitors in clinical trials is unpredictable at this stage. Here, we combined computer-aided hit identification, chemical synthesis, and *in vitro* biological evaluation to identify diverse GyrB-targeted antibacterial agents.

We concluded that the 4-hydroxy-2-quinolone fragment is essential to GyrB inhibition. Structural searches of the Specs compound library and experimental testing helped identify **f1** (**AG-690/11765367**) as a novel, moderate inhibitor of *S. aureus* GyrB (IC_50:_ 1.21 µM). This hit compound is of great interest because it contains a 4-oxoquinazolin moiety instead of the previously identified thiadiazole. According to the predicted binding mode of **f1**, the water-mediated interactions that involve the carbonyl group of the 4-oxoquinazolin moiety seem to be of relevance to bioactivity.

We also performed a preliminary SAR study by synthesising 15 new derivatives and evaluating their GyrB inhibitory activities in *S. aureus* Gyrase ATPase inhibition assays. This led to eight derivatives that were more potent than **f1**. Compounds **f4** and **f14** were the two most potent *S. aureus* GyrB inhibitors, with IC_50_ values of 0.31 and 0.28 µM, respectively. We compared these two derivatives with **f1** in terms of antibacterial activity against a panel of *S. aureus* strains and decided to select the initial hit compound **f1** for further testing in light of its better anti-MRSA activity (MICs: 4–8 µg/mL).

The *in vitro* cytotoxicity assay and metabolic stability assay (mouse) indicated that compound **f1** does not exhibit significant cytotoxicity against HUVECs and HepG2 cells and is metabolically stable. The compound has favourable physicochemical properties, although its aqueous solubility may require optimisation.

In summary, this study reports on the computer-aided discovery of N-quinazolinone-4-hydroxy-2-quinolone-3-carboxamides as new GyrB inhibitors. The GyrB inhibitor **f1** is a good starting structure for the development of new antibacterial agents.

## 4. Experimental

### 4.1. Substructure search

The protocol for “substructure search” implemented in Pipeline Pilot version 16.2.0.58; (Dassault Systèmes Biovia Corp., San Diego, CA) was used for our purpose. Here, the 4-hydroxy-2-quinolone scaffold (cf. Figure 2) was set as the substructure, while the Specs chemical library version 2015 (accessed at http://www.specs.net) that contains more than 210,000 compounds was selected as the screening database. The outputs from the protocol, i.e. the compounds with the 4-hydroxy-2-quinolone scaffold from the Specs chemical library, were further assigned to 30 subsets by clustering based on MACCS fingerprints. Clustering was performed with the “Cluster Ligands” protocol of Discovery Studio version 16.1.0; (Dassault Systèmes Biovia Corp., San Diego, CA) Finally, the list of compounds for further bioassays was determined according to the structural diversity shown by the clustering, commercial availability, and synthetic feasibility.

### 4.2. Molecular docking

The structural model of *S. aureus* GyrB that we previously generated^16^ was used for molecular docking of the new inhibitors. This model was derived from the crystal structure of *S. aureus* GyrB in complex with Novobiocin (PDB code 4URO; http://www.rcsb.org/). The binding site of this model was defined by Novobiocin in the crystal structure. Here, the conserved co-crystallised water (i.e. wat46 in the publication^25^) was retained, and the hydrogen bond acceptor on the guanidine of Arg144 was defined as a docking restraint. These settings aimed to facilitate the generation of binding poses that may form hydrogen bonds with the conserved water and the guanidine of Arg144.

Prior to molecular docking, a maximum of 200 ligand conformers were generated with OMEGA version 2.5.1.4 (OpenEye Scientific Software, Inc., Santa Fe, NM).^26^ These conformers were subsequently placed into the binding site of the protein model with OEDocking version 3.0.1 (OpenEye Scientific Software, Inc., Santa Fe, NM).^18^ Finally, the docking poses were visually inspected, and the most plausible binding pose was selected.

### 4.3. Chemistry

#### 4.3.1. General methods

All of the reagents were obtained from commercial sources and used without further purification unless stated otherwise. Thin-layer chromatography (TLC) on the silica gel plates GF254 (200–300 mm; Qingdao Haiyang Chemical Co., Ltd., Qingdao, China) with UV light illumination was used to monitor chemical reactions. ^1^H NMR (500 MHz) and ^13 ^C NMR (100 MHz) spectra were measured by Avance spectrometer (Bruker, Varian Mercury, Billerica, MA). Chemical shifts were reported in *δ* values (ppm) with tetramethylsilane as the internal standard. High-resolution mass spectrometry (HRMS) was performed using the Thermo Scientific^™^ Exactive^™^ Plus mass spectrometer (Thermo, Waltham, MA). The melting points were recorded with a Mettler Toledo melting point apparatus. The purity of all the target compounds was determined by high-performance liquid chromatography (HPLC) on a Waters Acquity machine with a BEH C18 column (1.7 µm, 50 × 2.1 mm); mobile phase A = water (containing 0.1% formic acid) and mobile phase B = acetonitrile; the flow rate was 0.25 mL/min.

#### 4.3.2. Preparation of intermediate b1

Intermediate **b1** was prepared according to the reported method.^27^ A solution of isatoic anhydride (**a1**, 1 equiv.) in dry DMF (10 mL) was treated with DIPEA (1.3 equiv.) and iodoethane (1.3 equiv.), and the mixture was heated to 45 °C and stirred for 10 h. After cooling to room temperature, diethylmalonate and sodium hydride were added to the solution under N_2_ atmosphere. The reaction mixture was heated to 70 °C and stirred for 8 h. The solution was then poured into 50 mL of cool water, and 4 M HCl was added to make its pH less than 5. The product was obtained after filtering and recrystallisation with EtOAc.

##### 4.3.2.1. Ethyl 1-ethyl-4-hydroxy-2-oxo-1,2-dihydroquinoline-3-carboxylate (**b1**)

Yield 44.3%, white solid. ESI-MS (*m/z*): 262.21 [M + H]^+^, ^1^H NMR (500 MHz, DMSO-d_6_) δ 13.08 (brs, 1H, OH), 8.09 (d, *J* = 7.9 Hz, 1H, aromatic H), 7.76 (t, *J* = 8.0 Hz, 1H, aromatic H), 7.58 (d, *J* = 8.5 Hz, 1H, aromatic H), 7.32 (t, *J* = 7.6 Hz, 1H, aromatic H), 4.35 (q, *J* = 7.2 Hz, 2H, N*CH_2_*CH_3_), 4.24 (q, *J* = 7.2 Hz, 2H, O*CH_2_*CH_3_), 1.34 (t, *J* = 7.2 Hz, 3H, NCH_2_*CH_3_*), 1.21 (t, *J* = 7.1 Hz, 3H, OCH_2_*CH_3_*).

#### 4.3.3. Preparation of intermediate c1

HCl (12 N, 5.0 mL) was added to a solution of the ester **b1** (1.0 mmol) dissolved in MeOH (5.0 mL). The solution was stirred at 65 °C for 10 h. The solvent was evaporated under reduced pressure. The residue was then washed with 2-propanol and dried, which afforded intermediate **c1**.

##### 4.3.3.1. 1-ethyl-4-hydroxy-2-oxo-1,2-dihydroquinoline-3-carboxylic acid (**c1**)

Yield 61.5%, white solid. ESI-MS (*m/z*): 232.34 [M-H]^+^, ^1^H NMR (500 MHz, DMSO-d_6_) δ 14.40 (brs, 1H, OH), 8.20 (d, *J* = 8.0 Hz, 1H, aromatic H), 7.97 (t, *J* = 7.9 Hz, 1H, aromatic H), 7.89 (d, *J* = 8.7 Hz, 1H, aromatic H), 7.54 (t, *J* = 7.6 Hz, 1H, aromatic H), 4.41 (q, *J* = 7.1 Hz, 2H, N*CH_2_*CH_3_), 1.30 (t, *J* = 7.1 Hz, 3H, NCH_2_*CH_3_*).

#### 4.3.4. General procedure a for preparation of intermediates e1–e16

The substituted acyl chlorides (1.2 equiv.) and Et_3_N (1.2 equiv.) were added to a DCM solution of differently substituted methyl 2-aminobenzoates (1 equiv.) The reaction mixture was stirred at room temperature for 3 h. The solvent was evaporated under reduced pressure. The residues were used for the next step without further purification.

The residues were then dissolved in ethanol, and hydrazine hydrate (1.5 equiv.) was added. The reaction mixture was stirred at 78 °C for 10 h. The residue was then obtained by filtering the mixture and washed with ethanol to afford the intermediates **e1–e16**.

##### 4.3.4.1. 3-amino-2-pentylquinazolin-4(3H)-one (e1)

Yield 80.5%, white solid. ESI-MS (*m/z*): 232.31 [M + H]^+^, ^1^H NMR (500 MHz, DMSO-d_6_) δ 8.13 (d, *J* = 8.0 Hz, 1H, aromatic H), 7.80 (t, *J* = 7.7 Hz, 1H, aromatic H), 7.65 (d, *J* = 8.1 Hz, 1H, aromatic H), 7.50 (t, *J* = 7.6 Hz, 1H, aromatic H), 5.76 (s, 2H, NH_2_), 2.97 (t, *J* = 7.7 Hz, 2H, *CH_2_*CH_2_CH_2_CH_2_CH_3_), 1.79 (p, *J* = 7.6 Hz, 2H, CH_2_*CH_2_*CH_2_CH_2_CH_3_), 1.43 (q, *J* = 7.3 Hz, 2H, CH_2_CH_2_*CH_2_*CH_2_CH_3_), 1.37–1.30 (m, 2H, CH_2_CH_2_CH_2_*CH_2_*CH_3_), 0.94 (t, *J* = 7.3 Hz, 3H, CH_2_CH_2_CH_2_CH_2_*CH_3_*).

##### 4.3.4.2. 3-amino-6-chloro-2-pentylquinazolin-4(3H)-one (e2)

Yield 69.3%, white solid. ESI-MS (*m/z*): 266.31 [M + H]^+^, ^1^H NMR (500 MHz, DMSO-d_6_) δ 8.06 (s, 1H, aromatic H), 7.81 (d, *J* = 8.6 Hz, 1H, aromatic H), 7.67 (d, *J* = 8.6 Hz, 1H, aromatic H), 5.79 (s, 2H, NH_2_), 2.95 (t, *J* = 7.8 Hz, 2H, *CH_2_*CH_2_CH_2_CH_2_CH_3_), 1.79 (p, *J* = 6.9 Hz, 2H, CH_2_*CH_2_*CH_2_CH_2_CH_3_), 1.41–1.36 (m, 4H, CH_2_CH_2_*CH_2_CH_2_*CH_3_), 0.92 (t, *J* = 6.3 Hz, 3H, CH_2_CH_2_CH_2_CH_2_*CH_3_*).

##### 4.3.4.3. 3-amino-6-methyl-2-pentylquinazolin-4(3H)-one (e3)

Yield 63.9%, white solid. ESI-MS (*m/z*): 246.23 [M + H]^+^, ^1^H NMR (500 MHz, DMSO-d_6_) δ 7.92 (s, 1H, aromatic H), 7.61 (d, *J* = 8.3 Hz, 1H, aromatic H), 7.54 (d, *J* = 8.2 Hz, 1H, aromatic H), 5.74 (s, 2H, NH_2_), 2.93 (t, *J* = 7.7 Hz, 2H, *CH_2_*CH_2_CH_2_CH_2_CH_3_), 2.46 (s, 3H, CH_3_), 1.79 (p, *J* = 7.3 Hz, 2H, CH_2_*CH_2_*CH_2_CH_2_CH_3_), 1.44–1.36 (m, 4H, CH_2_CH_2_*CH_2_CH_2_*CH_3_), 0.95–0.88 (m, 3H, CH_2_CH_2_CH_2_CH_2_*CH_3_*).

##### 4.3.4.4. 3-amino-6-methoxy-2-pentylquinazolin-4(3H)-one (e4)

Yield 61.7%, off-white solid. ESI-MS (*m/z*): 262.35 [M + H]^+^, ^1^H NMR (500 MHz, DMSO-d_6_) δ 7.59 (d, *J* = 8.9 Hz, 1H, aromatic H), 7.48 (d, *J* = 2.9 Hz, 1H, aromatic H), 7.39 (dd, *J* = 9.0, 3.0 Hz, 1H, aromatic H), 5.76 (s, 2H, NH_2_), 3.89 (s, 3H, OCH_3_), 2.92 (t, *J* = 7.8 Hz, 2H, *CH_2_*CH_2_CH_2_CH_2_CH_3_), 1.78 (p, *J* = 7.3 Hz, 2H, CH_2_*CH_2_*CH_2_CH_2_CH_3_), 1.44–1.35 (m, 4H, CH_2_CH_2_*CH_2_CH_2_*CH_3_), 0.91 (t, *J* = 6.9 Hz, 3H, CH_2_CH_2_CH_2_CH_2_*CH_3_*).

##### 4.3.4.5. 3-amino-2-pentyl-6-(trifluoromethyl)quinazolin-4(3H)-one (e5)

Yield 56.4%, white solid. ESI-MS (*m/*z): 300.27 [M + H]^+^, ^1^H NMR (500 MHz, DMSO-*d*_6_) δ 8.38 (s, 1H, aromatic H), 8.10 (dd, *J* = 8.7, 2.3 Hz, 1H, aromatic H), 7.85 (d, *J* = 8.7 Hz, 1H, aromatic H), 5.84 (s, 2H, NH_2_), 3.00 (t, *J* = 7.7 Hz, 2H, *CH_2_*CH_2_CH_2_CH_2_CH_3_), 1.82 (p, *J* = 7.4 Hz, 2H, CH_2_*CH_2_*CH_2_CH_2_CH_3_), 1.44–1.36 (m, 4H, CH_2_CH_2_*CH_2_CH_2_*CH_3_), 0.93 (t, *J* = 6.8 Hz, 3H, CH_2_CH_2_CH_2_CH_2_*CH_3_*).

##### 4.3.4.6. 3-amino-7-chloro-2-pentylquinazolin-4(3H)-one (e6)

Yield 50.3%, white solid. ESI-MS (*m/z*): 266.16 [M + H]^+^, ^1^H NMR (500 MHz, DMSO-d_6_) δ 8.10 (d, *J* = 8.4 Hz, 1H, aromatic H), 7.66 (s, 1H, aromatic H), 7.50 (d, *J* = 8.5 Hz, 1H, aromatic H), 5.76 (s, 2H, NH_2_), 2.94 (t, *J* = 7.7 Hz, 2H, *CH_2_*CH_2_CH_2_CH_2_CH_3_), 1.78 (p, *J* = 7.3 Hz, 2H, CH_2_*CH_2_*CH_2_CH_2_CH_3_), 1.42–1.35 (m,4H, CH_2_CH_2_*CH_2_CH_2_*CH_3_), 0.91 (t, *J* = 6.8 Hz, 3H, CH_2_CH_2_CH_2_CH_2_*CH_3_*).

##### 4.3.4.7. 3-amino-7-methyl-2-pentylquinazolin-4(3H)-one (e7)

Yield 45.8%, white solid. ESI-MS (*m/z*): 246.32 [M + H]^+^, ^1^H NMR (500 MHz, DMSO-d_6_) δ 7.92 (s, 1H, aromatic H), 7.61 (d, *J* = 8.3 Hz, 1H, aromatic H), 7.54 (d, *J* = 8.2 Hz, 1H, aromatic H), 5.74 (s, 2H, NH_2_), 2.93 (t, *J* = 7.7 Hz, 2H), 2.46 (s, 3H, CH_3_), 1.79 (p, *J* = 7.3 Hz, 2H, *CH_2_*CH_2_CH_2_CH_2_CH_3_), 1.42–1.35 (m, 4H, CH_2_CH_2_*CH_2_CH_2_*CH_3_), 0.95–0.88 (m, 3H, CH_2_CH_2_CH_2_CH_2_*CH_3_*).

##### 4.3.4.8. 3-amino-7-methoxy-2-pentylquinazolin-4(3H)-one (e8)

Yield 63.4%, white solid. ESI-MS (*m/z*): 262.34 [M + H]^+^, ^1^H NMR (500 MHz, DMSO-d_6_) δ 8.02 (d, *J* = 9.4 Hz, 1H, aromatic H), 7.07 (d, *J* = 5.7 Hz, 2H, aromatic H), 5.69 (s, 2H, NH_2_), 3.91 (s, 3H, OCH_3_), 2.93 (t, *J* = 7.7 Hz, 2H, *CH_2_*CH_2_CH_2_CH_2_CH_3_), 1.79 (p, *J* = 7.3 Hz, 2H, CH_2_*CH_2_*CH_2_CH_2_CH_3_), 1.43–1.35 (m, 4H, CH_2_CH_2_*CH_2_CH_2_*CH_3_), 0.96–0.89 (m, 3H, CH_2_CH_2_CH_2_CH_2_*CH_3_*).

##### 4.3.4.9. 3-amino-6,7-dimethoxy-2-pentylquinazolin-4(3H)-one (e9)

Yield 55.6%, white solid. ESI-MS (*m/z*): 292.16 [M + H]^+^, ^1^H NMR (500 MHz, DMSO-d_6_) δ 7.41 (s, 1H, aromatic H), 7.09 (s, 1H, aromatic H), 5.71 (s, 2H, NH_2_), 3.93 (s, 3H, OCH_3_), 3.90 (s, 3H, OCH_3_), 2.91 (t, *J* = 7.8 Hz, 2H, *CH_2_*CH_2_CH_2_CH_2_CH_3_), 1.78 (p, *J* = 7.4 Hz, 2H, CH_2_*CH_2_*CH_2_CH_2_CH_3_), 1.41–1.35 (m, 4H, CH_2_CH_2_*CH_2_CH_2_*CH_3_), 0.92 (t, *J* = 6.9 Hz, 3H, CH_2_CH_2_CH_2_CH_2_*CH_3_*).

##### 4.3.4.10. 3-amino-8-chloro-2-pentylquinazolin-4(3H)-one (e10)

Yield 39.8%, a white solid. ESI-MS (*m/z*): 266.34 [M + H]^+^, ^1^H NMR (500 MHz, DMSO-d_6_) δ 8.09 (d, *J* = 8.0 Hz, 1H, aromatic H), 7.96 (d, *J* = 7.7 Hz, 1H, aromatic H), 7.47 (t, *J* = 7.8 Hz, 1H, aromatic H), 5.80 (s, 2H, NH_2_), 2.99 (t, *J* = 7.6 Hz, 2H, *CH_2_*CH_2_CH_2_CH_2_CH_3_), 1.83 (p, *J* = 7.5 Hz, 2H, CH_2_*CH_2_*CH_2_CH_2_CH_3_), 1.45–1.32 (m, 4H, CH_2_CH_2_*CH_2_CH_2_*CH_3_), 0.93 (t, *J* = 6.9 Hz, 3H, CH_2_CH_2_CH_2_CH_2_*CH_3_*).

##### 4.3.4.11. 3-amino-8-methyl-2-pentylquinazolin-4(3H)-one (e11)

Yield 46.3%, white solid. ESI-MS (*m/z*): 246.51 [M + H]^+^, ^1^H NMR (500 MHz, DMSO-d_6_) δ 8.01 (d, *J* = 8.0 Hz, 1H, aromatic H), 7.45 (s, 1H, aromatic H), 7.32 (d, *J* = 8.1 Hz, 1H, aromatic H), 5.71 (s, 2H, NH_2_), 2.94 (t, *J* = 7.7 Hz, 2H, *CH_2_*CH_2_CH_2_CH_2_CH_3_), 2.47 (s, 3H, CH_3_), 1.79 (p, *J* = 7.2 Hz, 2H, CH_2_*CH_2_*CH_2_CH_2_CH_3_), 1.43–1.35 (m, 4H, CH_2_CH_2_*CH_2_CH_2_*CH_3_), 0.95–0.86 (m, 3H, CH_2_CH_2_CH_2_CH_2_*CH_3_*).

##### 4.3.4.12. 3-amino-8-methoxy-2-pentylquinazolin-4(3H)-one (e12)

Yield 54.8%, off-white solid. ESI-MS (*m/z*): 262.43 [M + H]^+^, ^1^H NMR (500 MHz, DMSO-d_6_) δ 7.67 (d, *J* = 7.9 Hz, 1H, aromatic H), 7.42 (t, *J* = 8.0 Hz, 1H, aromatic H), 7.33 (d, *J* = 7.9 Hz, 1H, aromatic H), 5.77 (s, 2H, NH_2_), 3.93 (s, 3H, OCH_3_), 2.94 (t, *J* = 7.8 Hz, 2H, *CH_2_*CH_2_CH_2_CH_2_CH_3_), 1.79 (p, *J* = 7.2 Hz, 2H, CH_2_*CH_2_*CH_2_CH_2_CH_3_), 1.44–1.36 (m, 4H, CH_2_CH_2_*CH_2_CH_2_*CH_3_), 0.92 (t, *J* = 6.8 Hz, 3H, CH_2_CH_2_CH_2_CH_2_*CH_3_*).

##### 4.3.4.13. 3-amino-2-methylquinazolin-4(3H)-one (e13)

Yield 64.7%, white solid. ESI-MS (*m/z*): 176.33 [M + H]^+^, ^1^H NMR (500 MHz, DMSO-d_6_) δ 8.13 (d, *J* = 8.0 Hz, 1H, aromatic H), 7.80 (t, *J* = 7.7 Hz, 1H, aromatic H), 7.62 (d, *J* = 8.2 Hz, 1H, aromatic H), 7.50 (t, *J* = 7.5 Hz, 1H, aromatic H), 5.83 (s, 2H, NH_2_), 2.61 (s, 3H, CH_3_).

##### 4.3.4.14. 3-amino-2-ethylquinazolin-4(3H)-one (e14)

Yield 59.6%, white solid. ESI-MS (*m/z*): 190.21 [M + H]^+^, ^1^H NMR (500 MHz, DMSO-d_6_) δ 8.12 (d, *J* = 5.9 Hz, 1H, aromatic H), 7.78 (t, *J* = 7.3 Hz, 1H, aromatic H), 7.64 (d, *J* = 6.4 Hz, 1H, aromatic H), 7.49 (t, *J* = 7.2 Hz, 1H, aromatic H), 5.77 (s, 2H, NH_2_), 2.99 (p, *J* = 6.7, 6.2 Hz, 2H, *CH_2_*CH_3_), 1.30 (q, *J* = 6.3, 5.7 Hz, 3H, CH_2_*CH_3_*).

##### 4.3.4.15. 3-amino-2-propylquinazolin-4(3H)-one (e15)

Yield 68.7%, white solid. ESI-MS (*m/z*): 204.41 [M + H]^+^, ^1^H NMR (500 MHz, DMSO-d_6_) δ 8.14 (d, *J* = 7.8 Hz, 1H, aromatic H), 7.80 (t, *J* = 7.8 Hz, 1H, aromatic H), 7.65 (d, *J* = 8.1 Hz, 1H, aromatic H), 7.51 (t, *J* = 7.5 Hz, 1H, aromatic H), 5.76 (s, 2H, NH_2_), 2.95 (t, *J* = 7.6 Hz, 2H, *CH_2_*CH_2_CH_3_), 1.83 (h, *J* = 7.4 Hz, 2H, CH_2_*CH_2_*CH_3_), 1.03 (t, *J* = 7.3 Hz, 3H, CH_2_CH_2_*CH_3_*).

##### 4.3.4.16. 3-amino-2-butylquinazolin-4(3H)-one (e16)

Yield 74.6%, white solid. ESI-MS (*m/z*): 218.42 [M + H]^+^, ^1^H NMR (500 MHz, DMSO-d_6_) δ 8.14 (d, *J* = 8.0 Hz, 1H, aromatic H), 7.80 (t, *J* = 7.6 Hz, 1H, aromatic H), 7.65 (d, *J* = 8.1 Hz, 1H, aromatic H), 7.51 (t, *J* = 7.5 Hz, 1H, aromatic H), 5.76 (s, 2H, NH_2_), 2.97 (t, *J* = 7.7 Hz, 2H, *CH_2_*CH_2_CH_2_CH_3_), 1.79 (p, *J* = 7.6 Hz, 2H, CH_2_*CH_2_*CH_2_CH_3_), 1.44 (h, *J* = 7.5 Hz, 2H, CH_2_CH_2_*CH_2_*CH_3_), 0.97 (t, *J* = 7.3 Hz, 3H, CH_2_CH_2_CH_2_*CH_3_*).

#### 4.3.5. General procedure B for preparation of target compounds (f1– f16)

At 0 °C, HATU (1.5 equiv.) was added to the solution of the acid **c1** (1.1 equiv.), followed by DIPEA (1.5 equiv.) dissolved in DMF (10 mL). After 10 min, the amines **e1–16** (1.0 equiv.) were added, and the reaction mixture was stirred for 48 h at room temperature. The mixture was poured into the cold water. The precipitate was collected and washed with water and dried with anhydrous Na_2_SO_4_. The crude solid was purified through a silica gel column chromatography to afford the target compounds **f1–16**. The purity of all tested compounds was >95%, as determined by HPLC analysis.

##### 4.3.5.1. N-(4-oxo-2-pentylquinazolin-3(4H)-yl)-1-ethyl-4-hydroxy-2-oxo-1,2-dihydroquinoline-3-carboxamide (f1)

Yield 21.5%, white solid, m.p.: 168.5–171.0 °C. ESI-MS (*m/z*): 447.22 [M + H]^+^, ^1^H NMR (500 MHz, DMSO-d_6_) δ 12.31 (s, 1H, NH), 8.18 (d, *J* = 8.0 Hz, 1H, aromatic H), 8.14 (d, *J* = 7.8 Hz, 1H, aromatic H), 7.90 (d, *J* = 8.0 Hz, 2H, aromatic H), 7.77 (d, *J* = 8.8 Hz, 1H, aromatic H), 7.72 (d, *J* = 8.2 Hz, 1H, aromatic H), 7.57 (t, *J* = 7.5 Hz, 1H, aromatic H), 7.45 (t, *J* = 7.7 Hz, 1H, aromatic H), 4.40 (dd, *J* = 13.6, 7.0 Hz, 2H, N*CH_2_*CH_3_), 2.85–2.75 (m, 2H, aliphatic H), 1.77 (dd, *J* = 15.8, 8.1 Hz, 2H, aliphatic H), 1.25 (s, 7H, aliphatic H), 0.85 (s, 3H, aliphatic H). ^13 ^C NMR (125 MHz, CDCl_3_-*d*) δ 171.95, 171.63, 161.89, 159.50 (C = O), 158.03 (C = N), 146.97, 139.38, 134.67, 127.32, 127.06, 126.50, 125.79, 122.63, 120.88, 115.62, 114.40, 96.61 (aromatic carbons), 36.43, 33.86, 31.36, 26.13, 22.31, 13.89, 12.78 (aliphatic carbons). HRMS calcd for C_25_H_26_N_4_O_4_ [M + H]^+^, 447.2036; found, 447.2027. HPLC purity: 97.30%.

##### 4.3.5.2. N-(6-chloro-4-oxo-2-pentylquinazolin-3(4H)-yl)-1-ethyl-4-hydroxy-2-oxo-1,2-dihydroquinoline-3-carboxamide (f2)

Yield 21.5%, pale yellow solid, m.p.: 141.5–143.1 °C. ESI-MS (*m/z*): 481.42 [M + H]^+^, ^1^H NMR (500 MHz, DMSO-d_6_) δ 8.16 (d, *J* = 7.9 Hz, 1H, aromatic H), 8.06 (d, *J* = 6.4 Hz, 1H, aromatic H), 7.88 (t, *J* = 8.9 Hz, 2H, aromatic H), 7.71–7.67 (m, 3H, aromatic H), 7.42–7.36 (m, 1H, aromatic H), 4.46–4.25 (m, 2H, N*CH_2_*CH_3_), 2.79–2.73 (m, 2H, aliphatic H), 1.78–1.74 (m, 2H, aliphatic H), 1.39–1.23 (m, 10H, aliphatic H). ^13 ^C NMR (100 MHz, DMSO-d_6_) δ 170.60, 161.81, 160.96, 160.02 (C = O), 159.53 (C = N), 145.85, 134.59, 133.47, 130.62, 129.66, 127.81, 126.35, 125.66, 125.19, 122.92, 121.41, 98.88 (aromatic carbons), 34.09, 33.34, 31.25, 25.59, 22.35, 14.23, 13.26 (aliphatic carbons). HRMS calcd for C_25_H_26_ClN_4_O_4_ [M + H]^+^, 481.1663; found, 481.1637. HPLC purity: 99.38%.

##### 4.3.5.3. N-(6-methyl-4-oxo-2-pentylquinazolin-3(4H)-yl)-1-ethyl-4-hydroxy-2-oxo-1,2-dihydroquinoline-3-carboxamide (f3)

Yield 36.9%, white solid, m.p.: 160.6–162.3 °C. ESI-MS (*m/z*): 461.15 [M + H]^+^, ^1^H NMR (500 MHz, CDCl_3_-d) δ 15.16 (s, 1H, OH), 12.57 (s, 1H, NH), 8.30 (d, *J* = 7.8 Hz, 1H, aromatic H), 8.10 (s, 1H, aromatic H), 7.80 (t, *J* = 7.9 Hz, 1H, aromatic H), 7.71 (d, *J* = 8.1 Hz, 1H, aromatic H), 7.63 (d, *J* = 8.0 Hz, 1H, aromatic H), 7.48 (d, *J* = 8.6 Hz, 1H, aromatic H), 7.39 (t, *J* = 7.6 Hz, 1H, aromatic H), 4.44 (q, *J* = 7.1 Hz, 2H, N*CH_2_*CH_3_), 2.91 (t, *J* = 7.6 Hz, 2H, aliphatic H), 2.53 (s, 3H, aliphatic H), 1.90 (p, *J* = 7.3 Hz, 2H, aliphatic H), 1.44 (q, *J* = 8.2, 7.7 Hz, 6H, aliphatic H), 0.93 (t, *J* = 7.0 Hz, 3H, aliphatic H). ^13 ^C NMR (125 MHz, CDCl_3_-*d*) δ 172.01, 171.64, 161.97, 159.54 (C = O), 157.21 (C = N), 144.91, 139.42, 136.75, 136.17, 134.74, 127.11, 126.58, 125.89, 122.66, 120.66, 115.72, 114.38, 96.71 (aromatic carbons), 37.61, 33.87, 31.46, 26.27, 22.36, 21.25, 13.94, 12.84 (aliphatic carbons). HRMS calcd for C_26_H_29_N_4_O_4_ [M + H]^+^, 461.2174; found, 461.2183. HPLC purity: 95.57%.

##### 4.3.5.4. N-(6-methoxy-4-oxo-2-pentylquinazolin-3(4H)-yl)-1-ethyl-4-hydroxy-2-oxo-1,2-dihydroquinoline-3-carboxamide (f4)

Yield 20.3%, white solid, m.p.: 98.7–99.9 °C. ESI-MS (*m/z*): 477.31 [M + H]^+^, ^1^H NMR (500 MHz, DMSO-d_6_) δ 12.31 (s, 1H, NH), 8.27 (d, *J* = 8.5 Hz, 1H, aromatic H), 8.02 (d, *J* = 8.4 Hz, 1H, aromatic H), 7.86 (t, *J* = 7.6 Hz, 1H, aromatic H), 7.77 (d, *J* = 8.8 Hz, 1H, aromatic H), 7.66 (t, *J* = 7.7 Hz, 2H, aromatic H), 7.54–7.49 (m, 1H, aromatic H), 4.41 (q, *J* = 6.8 Hz, 2H, N*CH_2_*CH_3_), 3.91 (s, 3H, OCH_3_), 2.56 (s, 2H, aliphatic H), 1.82–1.71 (m, 3H, aliphatic H), 1.32 (t, *J* = 7.0 Hz, 7H, aliphatic H), 0.85 (t, *J* = 7.0 Hz, 3H, aliphatic H). ^13 ^C NMR (100 MHz, DMSO-*d*_6_) δ 171.03, 160.96, 160.52, 157.86 (C = O), 157.17 (C = N), 142.86, 131.15, 127.93, 126.76, 120.90, 119.60, 110.05, 106.10, 98.86 (aromatic carbons), 56.12 (OCH_3_), 34.83, 33.68, 31.49, 26.14, 22.38, 14.34, 13.11 (aliphatic carbons). HRMS calcd for C_26_H_29_N_4_O_5_ [M + H]^+^, 477.2160; found, 477.2132. HPLC purity: 98.41%.

##### 4.3.5.5. N-(4-oxo-2-pentyl-6-(trifluoromethyl)quinazolin-3(4H)-yl)-1-ethyl-4-hydroxy-2-oxo-1,2-dihydroquinoline-3-carboxamide (f5)

Yield 19.8%, yellow solid, m.p.: 128.3–130.2 °C. ESI-MS (*m/z*): 515.32 [M + H]^+^, 1H NMR (500 MHz, CDCl_3_-d) δ 12.62 (s, 1H, NH), 8.56 (s, 1H, aromatic H), 8.28 (d, *J* = 8.0 Hz, 1H, aromatic H), 7.99 (d, *J* = 14.9 Hz, 1H, aromatic H), 7.85 (d, *J* = 8.6 Hz, 1H, aromatic H), 7.80 (t, *J* = 7.9 Hz, 1H, aromatic H), 7.48 (d, *J* = 8.6 Hz, 1H, aromatic H), 7.38 (t, *J* = 7.5 Hz, 1H, aromatic H), 4.42 (q, *J* = 7.2 Hz, 2H, N*CH_2_*CH_3_), 2.98 (s, 2H, aliphatic H), 1.90–1.87 (m, 2H, aliphatic H), 1.29–1.26 (m, 7H, aliphatic H), 0.91 (t, *J* = 7.1 Hz, 3H, aliphatic H). ^13 ^C NMR (100 MHz, DMSO-d_6_) δ 170.93, 161.45, 161.15, 160.41 (C = O), 158.34 (C = N), 149.43, 139.74, 135.81, 129.25, 128.90, 123.27, 120.98, 115.82, 96.47 (aromatic carbons), 36.90, 33.40, 25.93, 25.39, 22.25, 14.19, 13.17 (aliphatic carbons). HRMS calcd for C_26_H_26_F_3_N_4_O_4_ [M + H]^+^, 515.1912; found, 515.1901. HPLC purity: 99.91%.

##### 4.3.5.6. N-(7-chloro-4-oxo-2-pentylquinazolin-3(4H)-yl)-1-ethyl-4-hydroxy-2-oxo-1,2-dihydroquinoline-3-carboxamide (f6)

Yield 20.6%, pale yellow solid, m.p.: 128.4–130.2 °C. ESI-MS (*m/z*): 481.12 [M + H]^+^, ^1^H NMR (500 MHz, DMSO-d_6_) δ 12.32 (s, 1H, NH), 8.13 (t, *J* = 8.9 Hz, 2H, aromatic H), 7.88 (d, *J* = 8.1 Hz, 1H, aromatic H), 7.73 (d, *J* = 9.0 Hz, 1H, aromatic H), 7.59 (d, *J* = 8.6 Hz, 1H, aromatic H), 7.50 (d, *J* = 9.1 Hz, 1H, aromatic H), 7.43 (d, *J* = 7.7 Hz, 1H, aromatic H), 4.41–4.36 (m, 2H, N*CH_2_*CH_3_), 2.87–2.70 (m, 2H, aliphatic H), 1.80–1.74 (m, 2H, aliphatic H), 1.30 (t, *J* = 7.7 Hz, 7H, aliphatic H), 0.85 (t, *J* = 7.4 Hz, 3H, aliphatic H). ^13 ^C NMR (100 MHz, DMSO-d_6_) δ 171.69, 162.55, 161.14, 160.54 (C = O), 158.38 (C = N), 148.06, 139.08, 135.79, 131.78, 128.47, 126.75, 123.26, 121.44, 98.42 (aromatic carbons), 36.20, 34.17, 31.47, 26.01, 22.40, 14.35, 13.19 (aliphatic carbons). HRMS calcd for C_25_H_26_ClN_4_O_4_ [M + H]^+^, 481.1663; found, 481.1637. HPLC purity: 98.21%.

##### 4.3.5.7. N-(7-methyl-4-oxo-2-pentylquinazolin-3(4H)-yl)-1-ethyl-4-hydroxy-2-oxo-1,2-dihydroquinoline-3-carboxamide (f7)

Yield 18.7%, white solid, m.p.: 167.2–168.4 °C. ESI-MS (*m/z*): 461.14 [M + H]^+^, ^1^H NMR (500 MHz, DMSO-d_6_) δ 8.15 (t, *J* = 7.2 Hz, 1H, aromatic H), 8.03–7.98 (m, 1H, aromatic H), 7.86 (dd, *J* = 18.4, 8.5 Hz, 1H, aromatic H), 7.78–7.68 (m, 1H, aromatic H), 7.53–7.47 (m, 1H, aromatic H), 7.36 (d, *J* = 8.4 Hz, 2H, aromatic H), 4.41–4.30 (m, 2H, N*CH_2_*CH_3_), 2.84–2.81 (m, 2H, aliphatic H), 1.77–1.75 (m, 2H, aliphatic H), 1.31–1.28 (m, 7H, aliphatic H), 0.91 (t, *J* = 6.7 Hz, 3H, aliphatic H), 0.83 (t, *J* = 6.5 Hz, 3H, aliphatic H). ^13 ^C NMR (100 MHz, DMSO-d_6_) δ 170.93, 161.81, 160.94, 160.83 (C = O), 158.77 (C = N), 147.17, 127.89, 126.16, 122.89, 118.73, 117.88, 115.55, 98.88 (aromatic carbons), 34.06, 33.27, 31.23, 25.57, 22.35, 21.80, 14.23, 13.23 (aliphatic carbons). HRMS calcd for C_26_H_29_N_4_O_4_ [M + H]^+^, 461.2210; found, 461.2183. HPLC purity: 95.18%.

##### 4.3.5.8. N-(7-methoxy-4-oxo-2-pentylquinazolin-3(4H)-yl)-1-ethyl-4-hydroxy-2-oxo-1,2-dihydroquinoline-3-carboxamide (f8)

Yield 23.7%, white solid, m.p.: 146.8–147.6 °C. ESI-MS (*m/z*): 477.41 [M + H]^+^, ^1^H NMR (500 MHz, DMSO-d_6_) δ 8.16 (d, *J* = 8.1 Hz, 1H, aromatic H), 8.02 (t, *J* = 8.0 Hz, 2H, aromatic H), 7.84 (p, *J* = 8.9, 8.3 Hz, 1H, aromatic H), 7.70 (t, *J* = 8.6 Hz, 1H, aromatic H), 7.40 (dd, *J* = 16.0, 8.3 Hz, 1H, aromatic H), 7.06 (s, 1H, aromatic H), 4.34–4.31 (m, 2H, N*CH_2_*CH_3_), 3.94 (s, 3H, OCH_3_), 2.81–2.66 (m, 2H, aliphatic H), 1.77–1.75 (m, 2H, aliphatic H), 1.40–1.20 (m, 7H, aliphatic H), 0.85 (s, 3H, aliphatic H). ^13 ^C NMR (100 MHz, DMSO-d_6_) δ 170.86, 164.88, 164.19, 160.56 (C = O), 159.41 (C = N), 149.35, 149.30, 133.45, 130.35, 127.90, 122.91, 116.45, 113.78, 108.00, 98.88 (aromatic carbons), 56.27 (OCH_3_), 34.16, 33.42, 31.54, 26.14, 22.42, 14.25, 13.27 (aliphatic carbons). HRMS calcd for C_26_H_29_N_4_O_5_ [M + H]^+^, 477.2154; found, 477.2132. HPLC purity: 97.38%.

##### 4.3.5.9. N-(6,7-dimethoxy-4-oxo-2-pentylquinazolin-3(4H)-yl)-1-ethyl-4-hydroxy-2-oxo-1,2-dihydroquinoline-3-carboxamide (f9)

Yield 16.8%, white solid, m.p.: 172.4–174.1 °C. ESI-MS (*m/z*): 507.31 [M + H]^+^, ^1^H NMR (500 MHz, CDCl_3_-d) δ 15.19 (s, 1H, OH), 12.51 (s, 1H, NH), 8.25 (d, *J* = 8.0 Hz, 1H, aromatic H), 8.03 (s, 1H, aromatic H), 7.76 (t, *J* = 7.9 Hz, 1H, aromatic H), 7.59 (s, 1H, aromatic H), 7.44 (d, *J* = 8.7 Hz, 1H, aromatic H), 7.34 (t, *J* = 7.6 Hz, 1H, aromatic H), 4.43–4.35 (m, 2H, N*CH_2_*CH_3_), 4.03 (s, 3H, OCH_3_), 3.99 (s, 3H, OCH_3_), 2.87–2.78 (m, 2H, aliphatic H), 1.87 (dd, *J* = 11.4, 5.5 Hz, 2H, aliphatic H), 1.41 (t, *J* = 7.1 Hz, 7H, aliphatic H), 0.90 (t, *J* = 7.0 Hz, 3H, aliphatic H). ^13 ^C NMR (125 MHz, CDCl_3_-d) δ 172.02, 171.70, 162.52, 161.97 (C = O), 158.94 (C = N), 156.90, 155.22, 148.77, 143.35, 139.45, 134.70, 125.92, 122.64, 115.77, 114.37, 107.93, 106.13, 96.73 (aromatic carbons), 56.36 (OCH_3_), 56.27 (OCH_3_), 36.46, 33.93, 31.47, 26.37, 22.34, 13.93, 12.82 (aliphatic carbons). HRMS calcd for C_27_H_31_N_4_O_6_ [M + H]^+^, 507.2269; found, 507.2238. HPLC purity: 98.28%.

##### 4.3.5.10. N-(8-chloro-4-oxo-2-pentylquinazolin-3(4H)-yl)-1-ethyl-4-hydroxy-2-oxo-1,2-dihydroquinoline-3-carboxamide (f10)

Yield 23.5%, yellow solid, m.p.: 158.6–160.4 °C. ESI-MS (*m/z*): 481.11 [M + H]^+^, ^1^H NMR (500 MHz, DMSO-d_6_) δ 8.15 (dd, *J* = 16.8, 7.8 Hz, 1H, aromatic H), 8.08 (d, *J* = 8.0 Hz, 1H, aromatic H), 8.03–7.97 (m, 1H, aromatic H), 7.94 (d, *J* = 7.7 Hz, 1H, aromatic H), 7.83 (p, *J* = 8.3, 7.8 Hz, 1H, aromatic H), 7.74–7.66 (m, 1H, aromatic H), 7.45 (d, *J* = 8.0 Hz, 1H, aromatic H), 4.40–4.26 (m, 2H, N*CH_2_*CH_3_), 2.89–2.73 (m, 2H, aliphatic H), 1.82 (p, *J* = 7.5 Hz, 2H, aliphatic H), 1.40–1.38 (m, 3H, aliphatic H), 1.25–1.23 (m, 4H, aliphatic H), 0.92 (t, *J* = 7.0 Hz, 3H, aliphatic H). ^13 ^C NMR (100 MHz, DMSO-d_6_) δ 170.51, 162.76, 160.41, 159.75 (C = O), 158.99 (C = N), 143.48, 134.48, 130.83, 126.78, 125.56, 122.92, 121.83, 115.79, 115.69, 96.97 (aromatic carbons), 34.17, 33.46, 31.42, 25.82, 22.43, 14.36, 13.24 (aliphatic carbons). HRMS calcd for C_25_H_26_ClN_4_O_4_ [M + H]^+^, 481.1652; found, 481.1637. HPLC purity: 99.17%.

##### 4.3.5.11. N-(8-methyl-4-oxo-2-pentylquinazolin-3(4H)-yl)-1-ethyl-4-hydroxy-2-oxo-1,2-dihydroquinoline-3-carboxamide (f11)

Yield 13.6%, white solid, m.p.: 142.7–144.3 °C. ESI-MS (*m/z*): 461.32 [M + H]^+^, ^1^H NMR (500 MHz, DMSO-d_6_) δ 8.17 (d, *J* = 7.9 Hz, 1H, aromatic H), 7.96 (d, *J* = 8.0 Hz, 1H, aromatic H), 7.91–7.84 (m, 1H, aromatic H), 7.74 (q, *J* = 7.1 Hz, 2H, aromatic H), 7.65 (d, *J* = 7.4 Hz, 1H, aromatic H), 7.43 (t, *J* = 8.1 Hz, 1H, aromatic H), 4.38 (s, 2H, N*CH_2_*CH_3_), 2.89–2.69 (m, 2H, aliphatic H), 1.82–1.78 (m, 2H, aliphatic H), 1.38–1.26 (m, 7H, aliphatic H), 0.93 (t, *J* = 7.0 Hz, 3H, aliphatic H), 0.86 (s, 3H, aliphatic H). ^13 ^C NMR (100 MHz, DMSO-d_6_) δ 170.86, 161.82, 161.06, 160.96 (C = O), 157.37 (C = N), 145.45, 134.61, 126.00, 124.02, 122.92, 120.08, 115.60, 109.59, 98.88 (aromatic carbons), 33.92, 31.39, 25.75, 25.25, 22.41, 17.38, 14.26, 13.25 (aliphatic carbons). HRMS calcd for C_26_H_29_N_4_O_4_ [M + H]^+^, 461.2203; found, 461.2183. HPLC purity: 95.24%.

##### 4.3.5.12. N-(8-methoxy-4-oxo-2-pentylquinazolin-3(4H)-yl)-1-ethyl-4-hydroxy-2-oxo-1,2-dihydroquinoline-3-carboxamide (f12)

Yield 11.6%, white solid, m.p.: 178.3–180.1 °C. ESI-MS (*m/z*): 477.21 [M + H]^+^, ^1^H NMR (500 MHz, DMSO-d_6_) δ 8.19 (d, *J* = 8.1 Hz, 1H, aromatic H), 7.67 (d, *J* = 7.9 Hz, 1H, aromatic H), 7.55 (d, *J* = 8.5 Hz, 1H, aromatic H), 7.45 (t, *J* = 8.6 Hz, 1H, aromatic H), 7.41–7.37 (m, 2H, aromatic H), 7.31–7.26 (m, 1H, aromatic H), 4.32–4.27 (m, 2H, N*CH_2_*CH_3_), 3.96 (s, 3H, OCH_3_), 2.82–2.68 (m, 2H, aliphatic H), 1.78–1.76 (m, 2H, aliphatic H), 1.25–1.23 (m, 7H, aliphatic H), 0.86–0.83 (m, 3H, aliphatic H). ^13 ^C NMR (100 MHz, DMSO-d_6_) δ 170.33, 160.83, 159.55, 157.56 (C = O), 154.74 (C = N), 143.28, 137.74, 126.80, 122.55, 121.33, 117.79, 115.68, 114.74, 98.87 (aromatic carbons), 56.56 (OCH_3_), 34.43, 33.94, 31.42, 26.17, 22.32, 14.27, 13.39 (aliphatic carbons). HRMS calcd for C_26_H_29_N_4_O_5_ [M + H]^+^, 477.2162; found, 477.2132. HPLC purity: 96.30%.

##### 4.3.5.13. N-(2-methyl-4-oxoquinazolin-3(4H)-yl)-1-ethyl-4-hydroxy-2-oxo-1,2-dihydroquinoline-3-carboxamide (f13)

Yield 19.5%, white solid, m.p.: 168.5–170.3 °C. ESI-MS (*m/z*): 391.14 [M + H]^+^, ^1^H NMR (500 MHz, DMSO-d_6_) δ 8.21 (d, *J* = 7.8 Hz, 1H, aromatic H), 8.09 (d, *J* = 7.9 Hz, 1H, aromatic H), 7.83 (t, *J* = 7.8 Hz, 1H, aromatic H), 7.60–7.58 (m, 3H, aromatic H), 7.50 (t, *J* = 7.5 Hz, 1H, aromatic H), 7.37 (d, *J* = 8.6 Hz, 1H, aromatic H), 7.17 (q, *J* = 9.9, 7.0 Hz, 1H, aromatic H), 4.27–4.07 (m, 2H, N*CH_2_*CH_3_), 2.55 (s, 3H, CH_3_), 1.19–1.14 (m, 3H, NCH_2_*CH_3_*). ^13 ^C NMR (100 MHz, DMSO-d_6_) δ 176.40, 170.23, 164.16, 159.77 (C = O), 158.03 (C = N), 147.34, 139.39, 134.82, 132.30, 127.10, 122.91, 121.51, 118.25, 97.41 (aromatic carbons), 36.91, 22.07, 13.41 (aliphatic carbons). HRMS calcd for C_21_H_19_N_4_O_4_ [M + H]^+^, 391.1421; found, 391.1401. HPLC purity: 97.45%.

##### 4.3.5.14. N-(2-ethyl-4-oxoquinazolin-3(4H)-yl)-1-ethyl-4-hydroxy-2-oxo-1,2-dihydroquinoline-3-carboxamide (f14)

Yield 10.9%, grey solid, m.p.: 140.8–142.6 °C. ESI-MS (*m/z*): 405.21 [M + H]^+^, ^1^H NMR (500 MHz, DMSO-d_6_) δ 8.18 (d, *J* = 8.0 Hz, 1H, aromatic H), 8.14 (d, *J* = 7.9 Hz, 1H, aromatic H), 7.87 (t, *J* = 7.7 Hz, 1H, aromatic H), 7.81 (d, *J* = 8.6 Hz, 1H, aromatic H), 7.70 (d, *J* = 8.7 Hz, 1H, aromatic H), 7.67–7.59 (m, 1H, aromatic H), 7.55 (t, *J* = 7.2 Hz, 1H, aromatic H), 7.40–7.38 (m, 1H, aromatic H), 4.42–4.28 (m, 2H, N*CH_2_*CH_3_), 2.86–2.83 (m, 2H, *CH_2_*CH_3_), 1.34–1.28 (m, 3H, NCH_2_*CH_3_*), 1.28–1.21 (m, 3H, CH_2_*CH_3_*). ^13 ^C NMR (100 MHz, DMSO-d_6_) δ 170.79, 162.56, 161.49, 160.84 (C = O), 159.51 (C = N), 159.21, 147.08, 139.62, 135.27, 134.40, 127.55, 126.85, 121.15, 114.70, 98.42 (aromatic carbons), 37.26, 26.74, 13.26, 10.66 (aliphatic carbons). HRMS calcd for C_22_H_21_N_4_O_4_ [M + H]^+^, 405.1583; found, 405.1557. HPLC purity: 99.59%.

##### 4.3.5.15. N-(4-oxo-2-propylquinazolin-3(4H)-yl)-1-ethyl-4-hydroxy-2-oxo-1,2-dihydroquinoline-3-carboxamide (f15)

Yield 15.9%, grey solid, m.p.: 158.6–160.7 °C. ESI-MS (*m/z*): 419.32 [M + H]^+^, ^1^H NMR (500 MHz, DMSO-d_6_) δ 8.17 (dd, *J* = 14.5, 7.9 Hz, 2H, aromatic H), 7.90 (t, *J* = 7.9 Hz, 2H, aromatic H), 7.73 (t, *J* = 10.1 Hz, 2H, aromatic H), 7.57 (t, *J* = 7.6 Hz, 1H, aromatic H), 7.47–7.40 (m, 1H, aromatic H), 4.39–4.35 (m, 2H, N*CH_2_*CH_3_), 2.85–2.83 (m, 2H, *CH_2_*CH_2_CH_3_), 1.81 (h, *J* = 8.2, 7.6 Hz, 2H, CH_2_*CH_2_*CH_3_), 1.30 (dt, *J* = 15.0, 6.6 Hz, 3H, NCH_2_*CH_3_*), 0.98–0.96 (m, 3H, CH_2_CH_2_*CH_3_*). ^13 ^C NMR (100 MHz, DMSO-d_6_) δ 170.84, 160.97, 159.34, 158.65 (C = O), 147.06 (C = N), 138.88, 135.35, 134.43, 126.85, 122.72, 121.18, 115.63, 96.77 (aromatic carbons), 37.22, 35.19, 19.31, 14.10, 13.28 (aliphatic carbons). HRMS calcd for C_23_H_23_N_4_O_4_ [M + H]^+^, 419.1739; found, 419.1714. HPLC purity: 97.17%.

##### 4.3.5.16. N-(2-butyl-4-oxoquinazolin-3(4H)-yl)-1-ethyl-4-hydroxy-2-oxo-1,2-dihydroquinoline-3-carboxamide (f16)

Yield 18.1%, white solid, m.p.: 139.8–140.5 °C. ESI-MS (*m/z*): 433.14 [M + H]^+^, ^1^H NMR (500 MHz, DMSO-d_6_) δ 8.15 (t, *J* = 9.4 Hz, 2H, aromatic H), 7.88 (t, *J* = 7.8 Hz, 2H, aromatic H), 7.71 (d, *J* = 8.5 Hz, 2H, aromatic H), 7.56 (t, *J* = 7.6 Hz, 1H, aromatic H), 7.42 (d, *J* = 9.7 Hz, 1H, aromatic H), 4.38 (q, *J* = 9.5 Hz, 2H, N*CH_2_*CH_3_), 2.89–2.81 (m, 2H, *CH_2_*CH_2_CH_2_CH_3_), 1.75 (p, *J* = 7.2 Hz, 2H, CH_2_*CH_2_*CH_2_CH_3_), 1.40 (p, *J* = 7.5 Hz, 2H, CH_2_CH_2_*CH_2_*CH_3_), 1.33–1.26 (m, 3H, NCH_2_*CH_3_*), 0.90 (t, *J* = 7.4 Hz, 3H, CH_2_CH_2_CH_2_*CH_3_*). ^13 ^C NMR (100 MHz, DMSO-d_6_) δ 170.90, 160.95, 159.63, 159.11 (C = O), 158.84 (C = N), 147.06, 139.66, 135.35, 127.55, 126.85, 122.92, 121.10, 115.60, 96.67 (aromatic carbons), 36.92, 33.01, 28.06, 22.16, 14.22, 13.25 (aliphatic carbons). HRMS calcd for C_24_H_25_N_4_O_4_ [M + H]^+^, 433.1854; found, 433.1870. HPLC purity: 96.31%.

### 4.4. Bioassays

#### 4.4.1. S. aureus GyrB inhibition assay

The IC_50_ values for *S. aureus* GyrB were determined according to our previously reported protocol.^16^ For this assay, the reaction mixture (10 μL) consisted of 5 nM *S. aureus* Gyrase (Inspiralis Ltd., Norwich, UK), assay buffer [40 mM HEPES-KOH (pH 7.6), 10 mM magnesium acetate, 10 mM dithiothreitol, 50 g/L BSA, and 500 mM potassium glutamate], the test compound (0.01–100 μM), 1% DMSO, 10 nM linear pBR322 DNA, and 100 mM ATP. The target compound was dissolved in DMSO (10 mM). A series of dilutions (0.1–1000 μM) were prepared from the stock solution with the assay buffer and DMSO. The dilutions (1 μL) were respectively added to the PCR tubes along with the buffer (7 μL), the linear pBR322 DNA (0.5 μL), *S. aureus* Gyrase (0.5 μL), and ATP (1 μL). The PCR tubes were sealed and incubated at 37 °C for 30 min. The ADP-Glo reagent (40 μL) was added to stop the reaction and use up the remaining ATP.

The detection reagent (50 μL) was then added and mixed. After 5 min, the mixture in each PCR tube was put in the well of a 96-well plate and its luminescence was measured by the BioTek Synergy 2 microplate reader. The activity values (%) of *S. aureus* Gyrase treated by the test compound at different concentrations were determined based on the luminescence. GraphPad Prism version 5 software (GraphPad Software Inc., La Jolla, CA) was used to calculate the IC_50_ values. Novobiocin was used as the positive control to ensure that the assay was reliable;^28^ the assay was performed in duplicate.

#### 4.4.2. Minimal inhibitory concentration (MIC) measurement

All of the bacterial strains used in this study were obtained from the Collection Centre of Pathogen Microorganism of Chinese Academy of Medical Sciences (CAMS-CCPM-A) in China. All of the isolates were stored at −80 °C and streaked on tryptic soy agar (TSA) plates to obtain overnight cultures. The MICs of the test compounds were determined by the broth microdilution method in accordance with the Clinical and Laboratory Standards Institute (CLSI) guidelines.^20^ Cation-adjusted Mueller-Hinton broth (CAMHB) was used as the growth medium for MIC assays. Briefly, 100 μL of serially diluted compounds (starting concentrations were 64 μg/mL) in CAMH broth were added to the wells of 96-well microtiter plates. Then, 10 µL of 5 × 10^6^ CFU/mL bacterial culture was added to each well. The plates were incubated at 37 °C for 18–20 h prior to the MIC determination. The MIC was defined as the lowest concentration of a compound that inhibited visual growth of the bacteria. Vancomycin was set as the positive control for this assay.

#### 4.4.3. Cytotoxicity assay

Cytotoxicity in terms of CC_50_ was determined by the SRB assay according to the protocol described in our previous publication.^16^ Briefly, human HUVECs or HepG2 cells were seeded in the wells of 96-well plates at a concentration of 1 × 10^5^ cells per well and incubated at 37 °C for 24 h. The cells were treated with the serially diluted compounds (10 nM and 100 μM) and incubated at 37 °C with 5% CO_2_ for 48 h. The cells were fixed with 10% trichloroacetic acid *(w/v)* at 4 °C for 1 h, washed five times with distilled water, and stained with 0.4% SRB solution at room temperature for another 20 min. The cells were then washed five times with 1% acetic acid and air-dried. The protein-bound dye was dissolved in 10 mM Tris-base solution and shaken for 5 min. The optical density (OD) was measured at 540 nm using a microplate reader to estimate the cell viability (%). With the viability values (%) of the cells treated by the compound at different concentrations as input, the CC_50_ value was determined by the non-linear regression with normalised dose-response fit implemented in GraphPad Prism version 5 software (GraphPad Software Inc., La Jolla, CA). The experiment was performed in duplicate, and paclitaxel was used as the positive drug for this assay.

#### 4.4.4. Mouse plasma stability assay

The mouse plasma was purchased from Charles River and stored at −20 °C. The stock solution of the test compound dissolved in DMSO (50 mM) was prepared and diluted to obtain a working solution at the concentration of 0.2 mM. The working solution of the control compound, i.e. propantheline, was similarly prepared (0.2 mM). Terfenadine (5 ng/mL) and tolbutamide (10 ng/mL) dissolved in acetonitrile were used as two quenching solutions.

The mouse plasma was pre-warmed at 37 °C for 15 min. The working solution of the test/control compound (2 µL) was then added to the mouse plasma (398 µL) in a 96-well plate. Next, 30 µL of the reaction mixture was sampled and moved to 300 µL of quenching solutions at each time point: 0, 5, 15, 30, 60, and 120 min for the control compound and 0, 15, 30, 60, and 120 min for the test compound. When the sampling was done, each sample was centrifuged at 4000 rpm at 4 °C for 15 min. Then, 100 µL of the supernatant was removed and mixed with 100 µL of distilled water for LC-MS/MS analysis with Shimadzu HPLC system and the AB Sciex API 4000 QTRAP instrument. The remaining compound (%) after incubation in plasma versus the incubation time was plotted. The half-life (*t*_1/2_) value of the compound was determined *via* linear regression from the plot.

#### 4.4.5. Mouse microsomal stability assay

The mouse liver microsomes were purchased from BioreclamationIVT and stored at −80 °C. The working solutions (0.2 mM) of the control compound, i.e. dextromethorphan, and the test compound dissolved in DMSO were prepared. Terfenadine (5 ng/mL) and tolbutamide (10 ng/mL) dissolved in acetonitrile were used as two quenching solutions. NADPH was dissolved in the phosphate buffer (50 mM K2HPO4, pH 7.4) at 5 mM. The mouse liver microsomes were thawed at 37 °C. The concentration of the stock solution was 20 mg/mL. The working solution (0.629 mg/mL) was prepared by dilution in phosphate buffer.

The working solution of the control/test compound (1.5 µL) was mixed with the liver microsome working solution (238.5 µL) in a 96-well plate and pre-incubated at 37 °C for 5 min. The 5 mM NADPH working solution (60 µL) was added to the solution to initiate the metabolic reaction. At each time point (0, 5, 15, 30, and 60 for the control/test compound), 30 µL of the reaction mixture was sampled and moved to 300 µL of quenching solutions. When the sampling was done, each sample was centrifuged at 4000 rpm at 4 °C for 15 min. The supernatant was analysed with the same HPLC system as the plasma stability assay. The residues (%) of the test/control compound along with the incubation time were calculated based on which the half-life (*t*_1/2_) value was determined.

## Supplementary Material

Supplemental Material
