# Supplementary material for: Discovery of N-quinazolinone-4-hydroxy-2-quinolone-3-carboxamides as DNA gyrase B-targeted antibacterial agents
Source: J Enzyme Inhib Med Chem. 2022 Jun 7;37(1):1620–31. doi: 10.1080/14756366.2022.2084088 (PMC9186351; doi:10.1080/14756366.2022.2084088)
Supplement: Supplemental Material [file IENZ_A_2084088_SM8912.pdf]

SUPPORTING INFORMATION

Discovery of *N*-quinazolinone-4-hydroxy-2-quinolone-3-carboxamides as DNA gyrase B-targeted antibacterial agents

Wenjie Xue[a,b], Yaling Wang[a,c], Xu Lian[a], Xueyao Li[a], Jing Pang[d], Johannes Kirchmair[e], Kebin Wu[f], Zunsheng Han [a], Xuefu You[d], Hongmin Zhang[f], Jie Xia[a,*] and Song Wu[a,*]

[a] *State Key Laboratory of Bioactive Substance and Function of Natural Medicines, Department of New Drug Research and Development, Institute of Materia Medica, Chinese Academy of Medical Sciences and Peking Union Medical College, Beijing 100050, China;* [b] *Department of Pharmacy, Shanxi Bethune Hospital, Shanxi Academy of Medical Sciences, Tongji Shanxi Hospital, Third Hospital of Shanxi Medical University, Taiyuan 030032, China;* [c] *School of Pharmacy, Jiangsu Ocean University, Lianyungang 222005, China;* [d]*Institute of Medicinal Biotechnology, Chinese Academy of Medical Sciences and Peking Union Medical College, Beijing 100050, China;* [e] *Division of Pharmaceutical Chemistry, Department of Pharmaceutical Sciences, University of Vienna, 1090 Vienna, Austria;* [f] *Department of Biology, Guangdong Provincial Key Laboratory of Cell Microenvironment and Disease Research, Shenzhen Key Laboratory of Cell Microenvironment and SUSTech-HKU Joint Laboratories for Matrix Biology, Southern University of Science and Technology, Shenzhen 518055, China*

[*]Correspondence should be addressed to J.X. (jie.william.xia@hotmail.com) or S.W. (ws@imm.ac.cn)

Figure S1. ^{1}H NMR and ^{13}C NMR spectra of all the synthesized *N*-(4-oxoquinazolin-3(4*H*)-yl)-4-hydroxy-2-quinolone-3-carboxamides.

*1-ethyl-N-(4-oxoquinazolin-3(4H)-yl)-4-hydroxy-2-oxo-1,2-dihydroquinoline-3-carboxamide**(f1)***

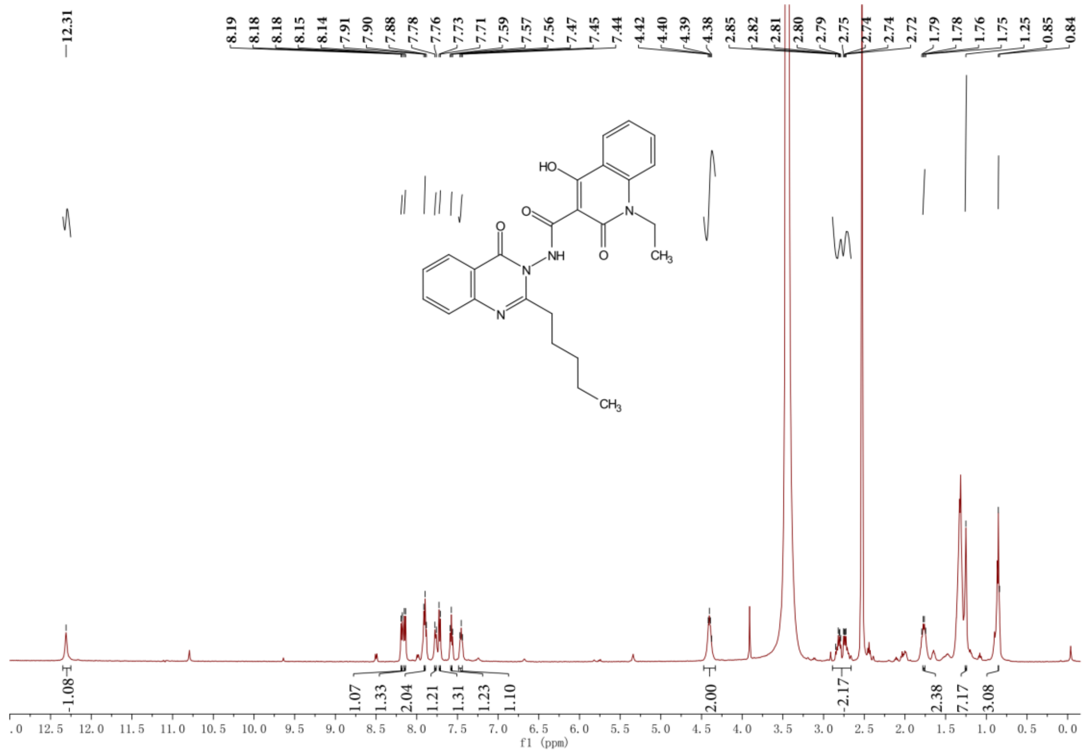

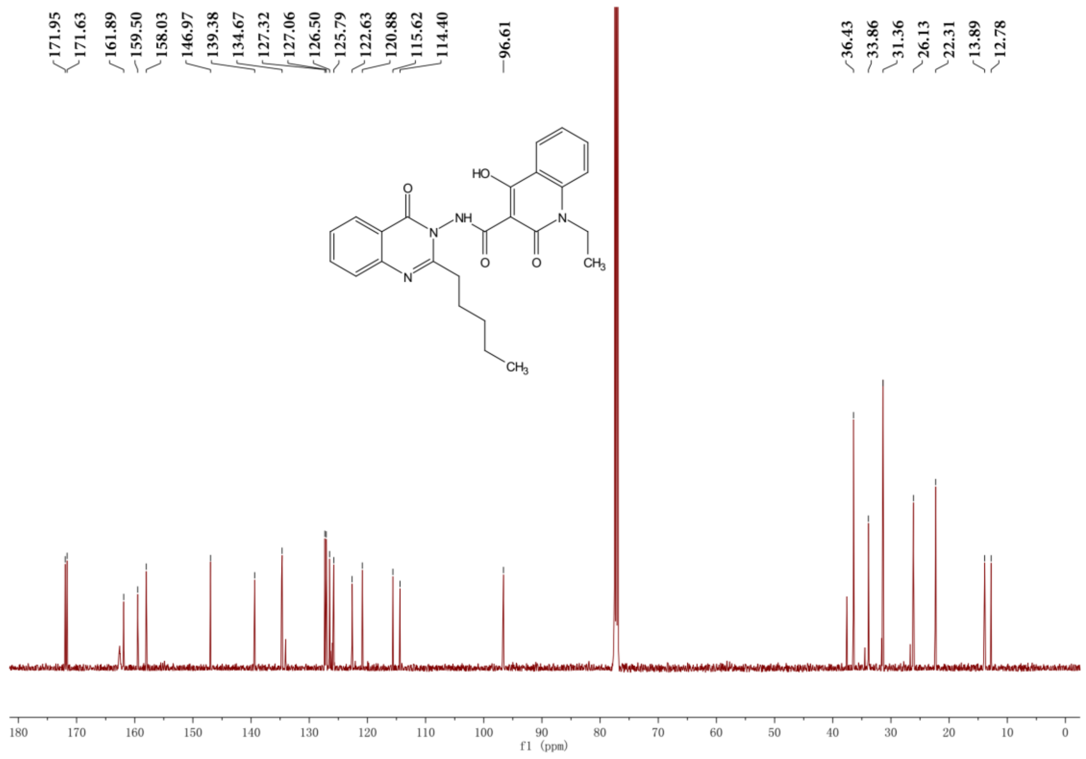

N-(6-chloro-4-oxo-2-pentylquinazolin-3(4H)-yl)-1-ethyl-4-hydroxy-2-oxo-1,2-dihydro quinoline-3-carboxamide ***(f2)***

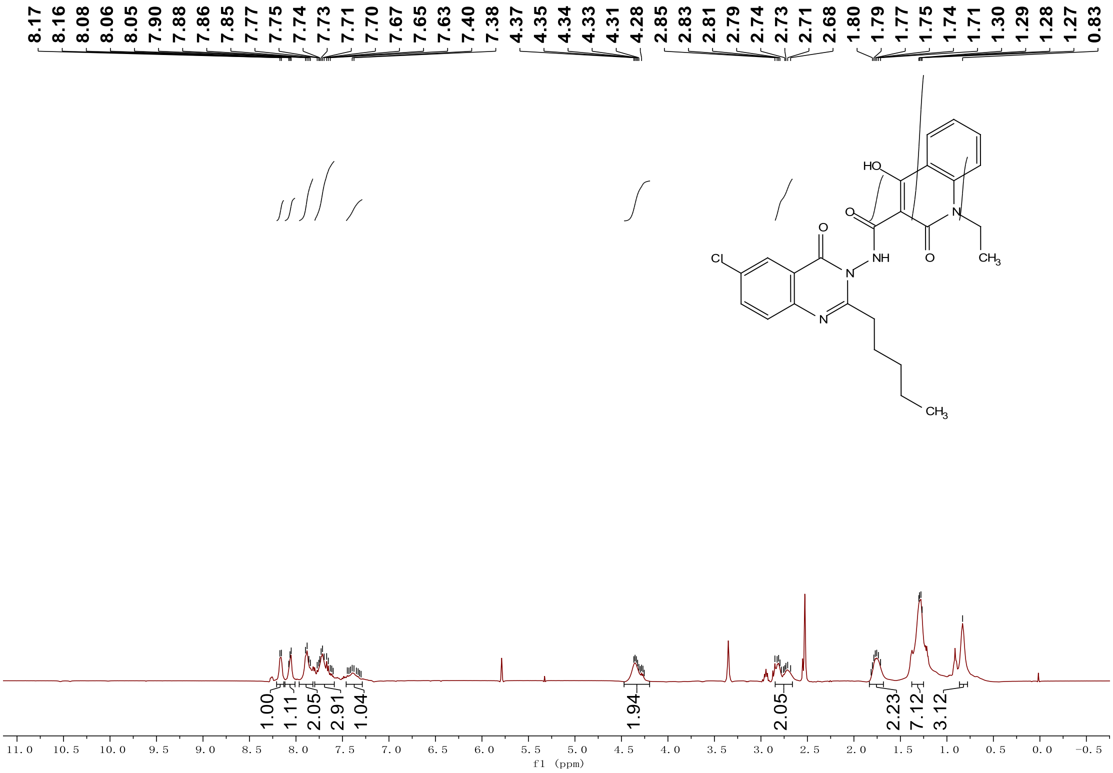

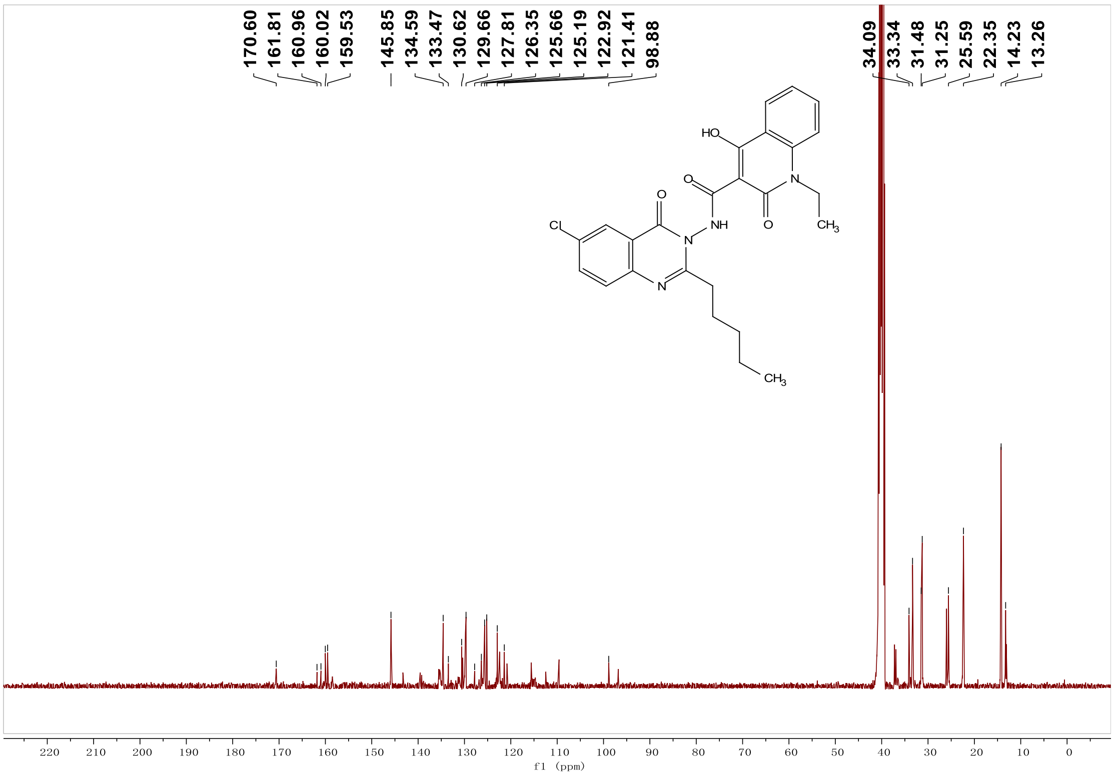

N-(6-methyl-4-oxo-2-pentylquinazolin-3(4H)-yl)-1-ethyl-4-hydroxy-2-oxo-1,2-dihydro quinoline-3-carboxamide ***(f3)***

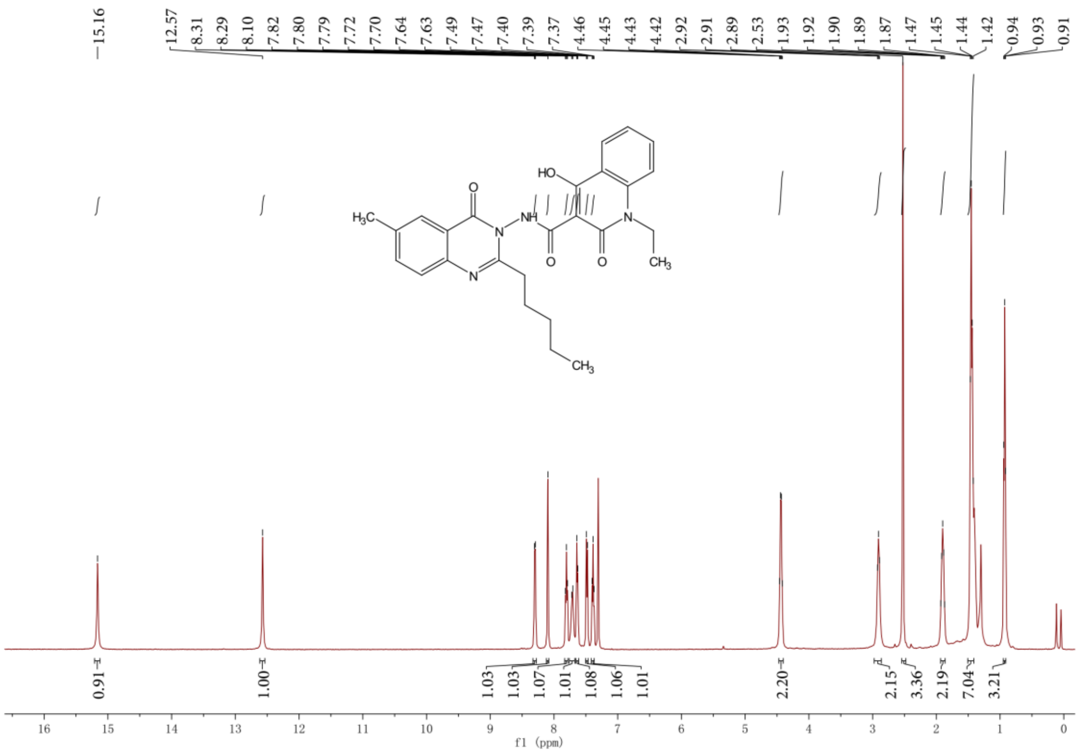

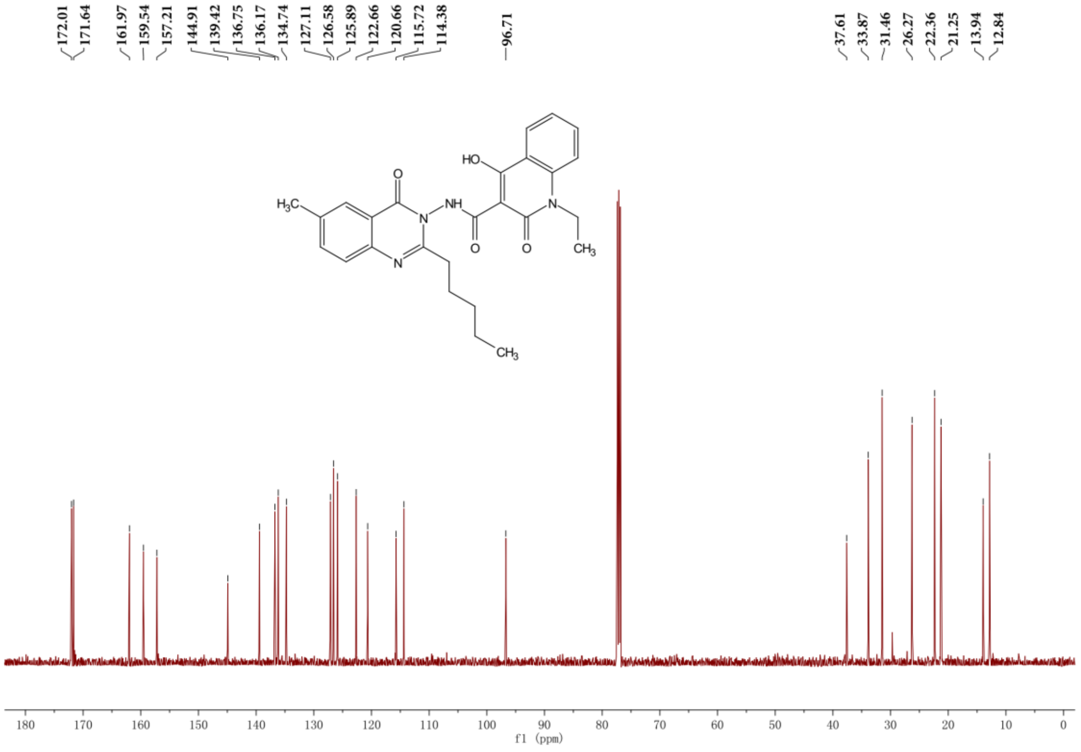

N-(6-methoxy-4-oxo-2-pentylquinazolin-3(4H)-yl)-1-ethyl-4-hydroxy-2-oxo-1,2-dihydroquinoline-3-carboxamide ***(f4)***

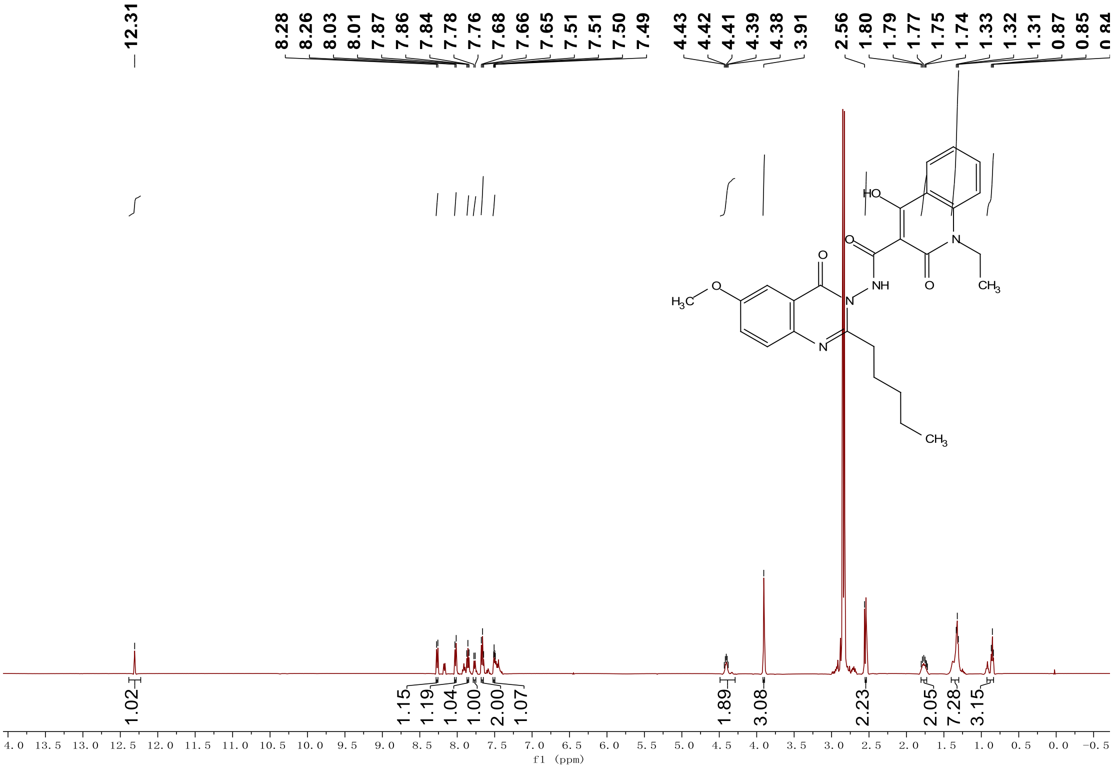

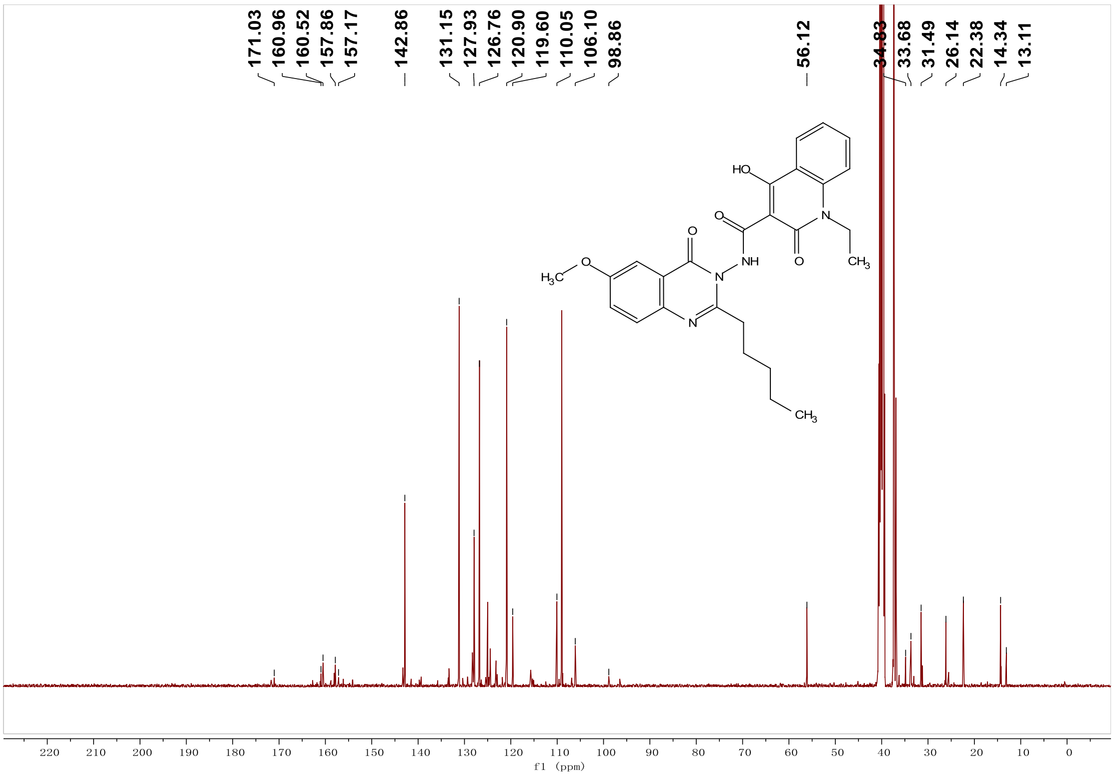

N-(4-oxo-2-pentyl-6-(trifluoromethyl)quinazolin-3(4H)-yl)-1-ethyl-4-hydroxy-2-oxo-1,2-dihydroquinoline-3-carboxamide ***(f5)***

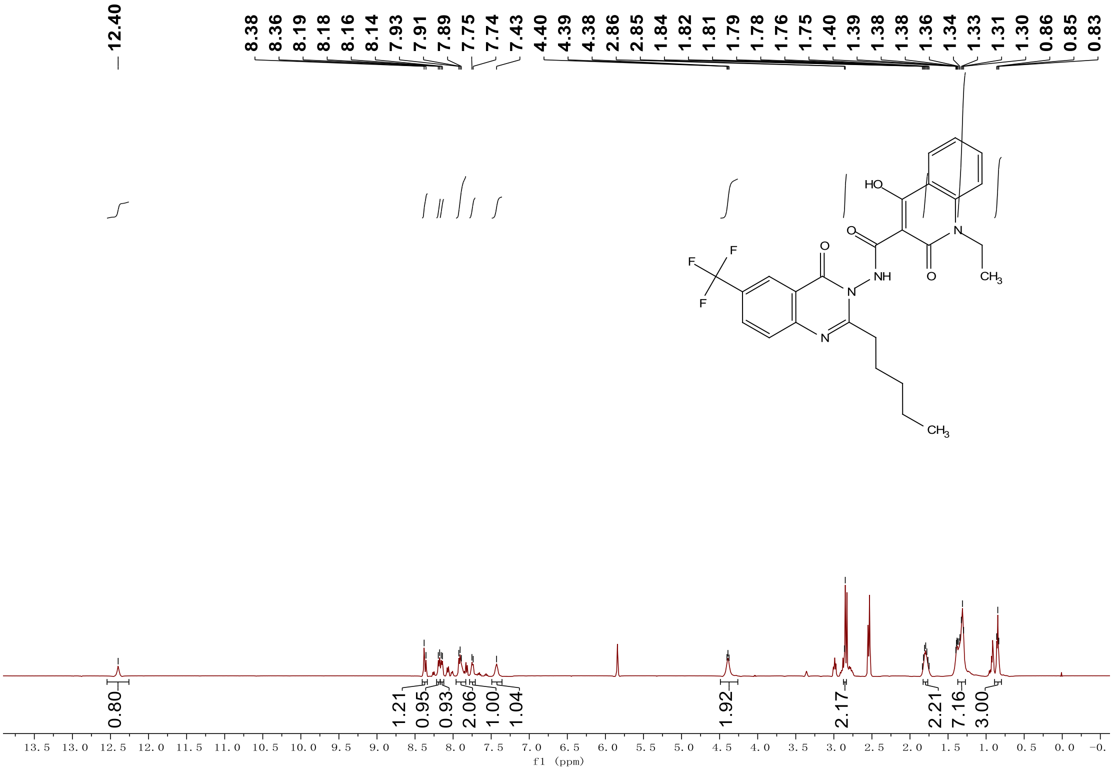

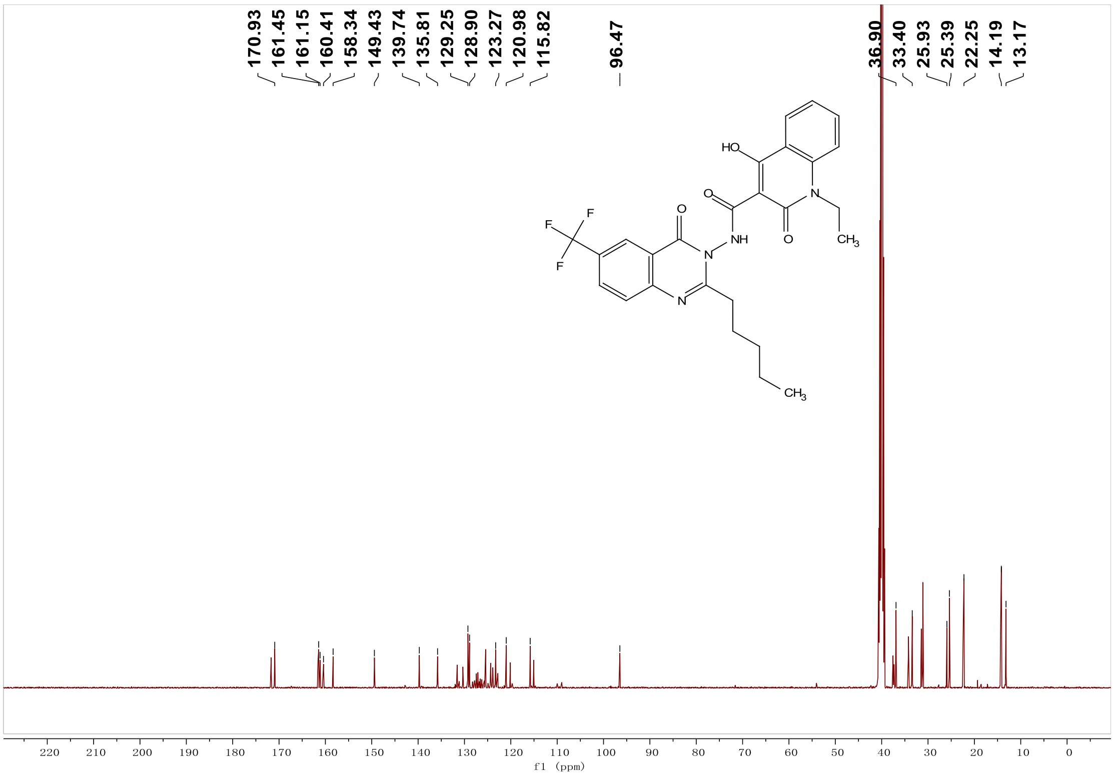

N-(7-chloro-4-oxo-2-pentylquinazolin-3(4H)-yl)-1-ethyl-4-hydroxy-2-oxo-1,2-dihydro quinoline-3-carboxamide ***(f6)***

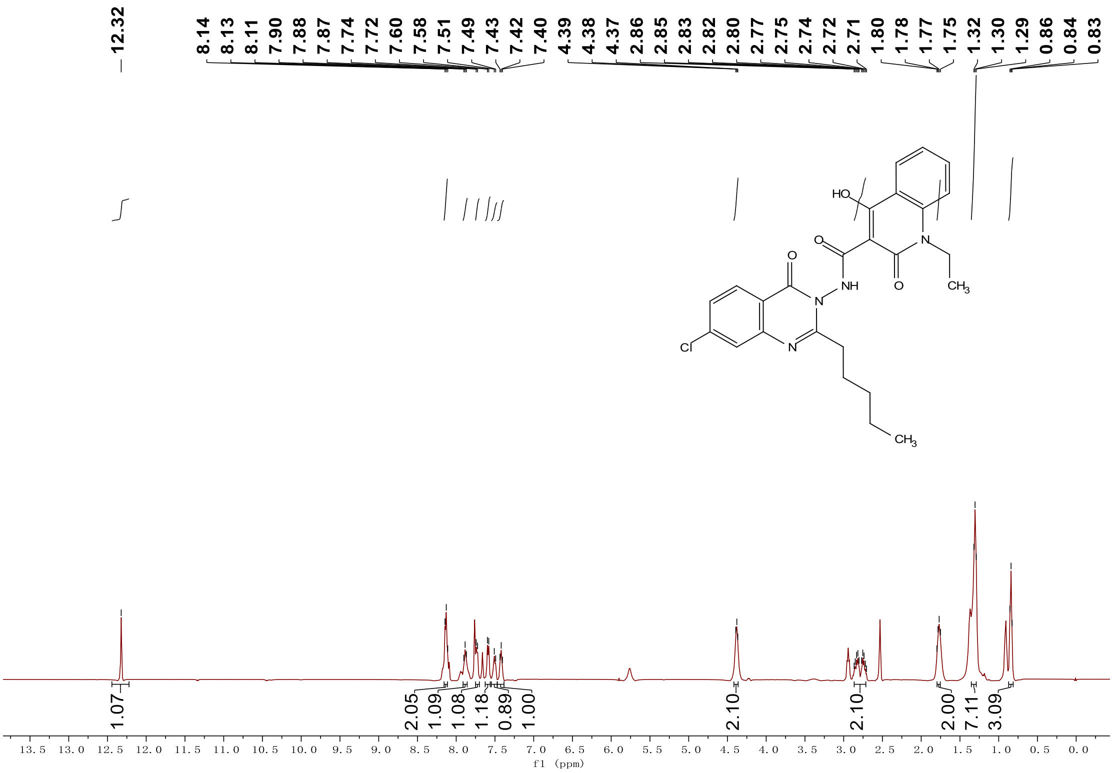

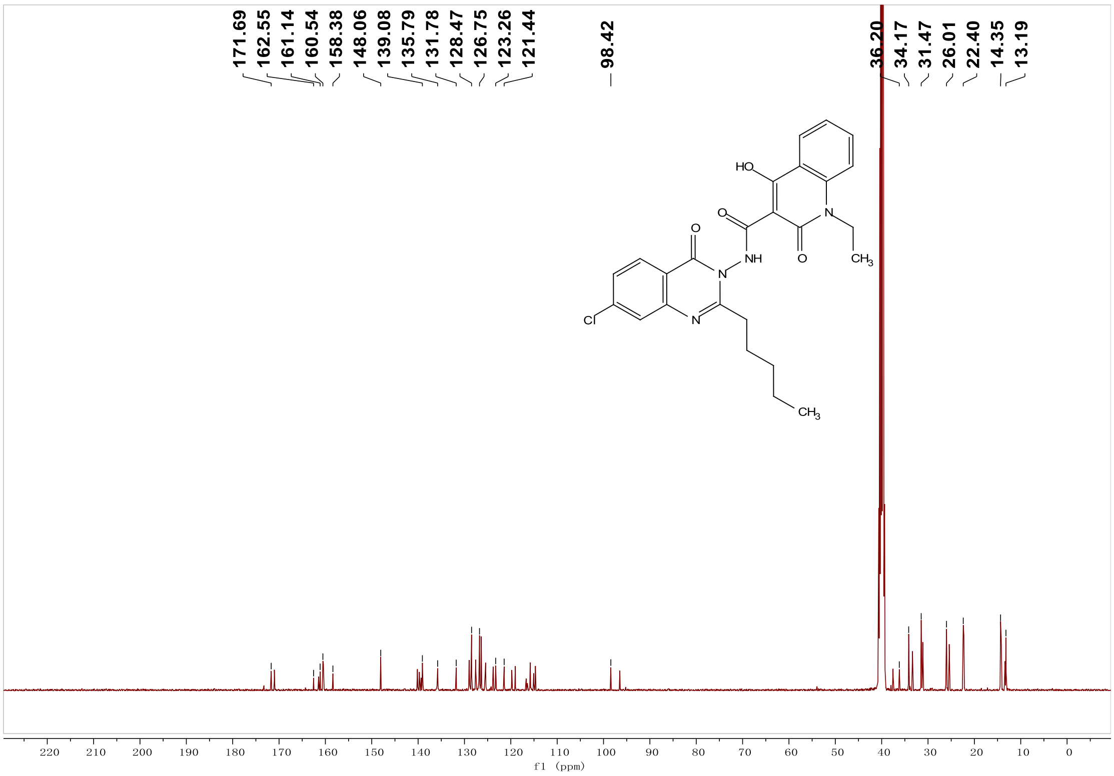

N-(7-methyl-4-oxo-2-pentylquinazolin-3(4H)-yl)-1-ethyl-4-hydroxy-2-oxo-1,2-dihydro quinoline-3-carboxamide ***(f7)***

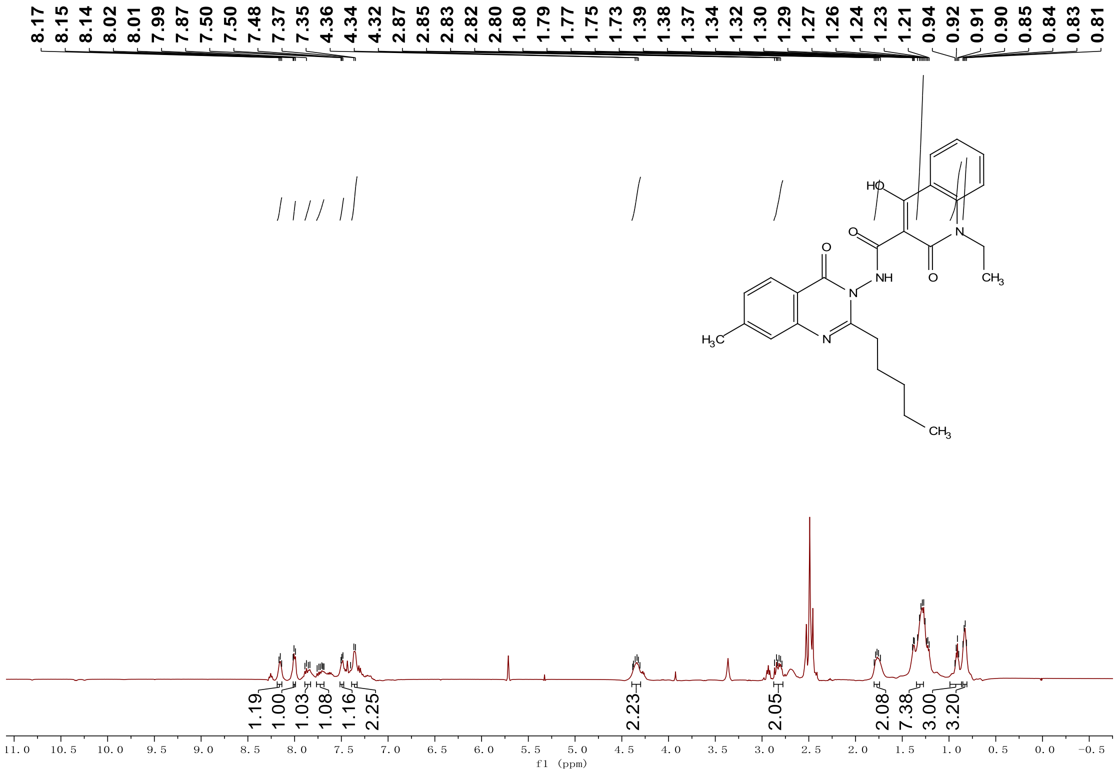

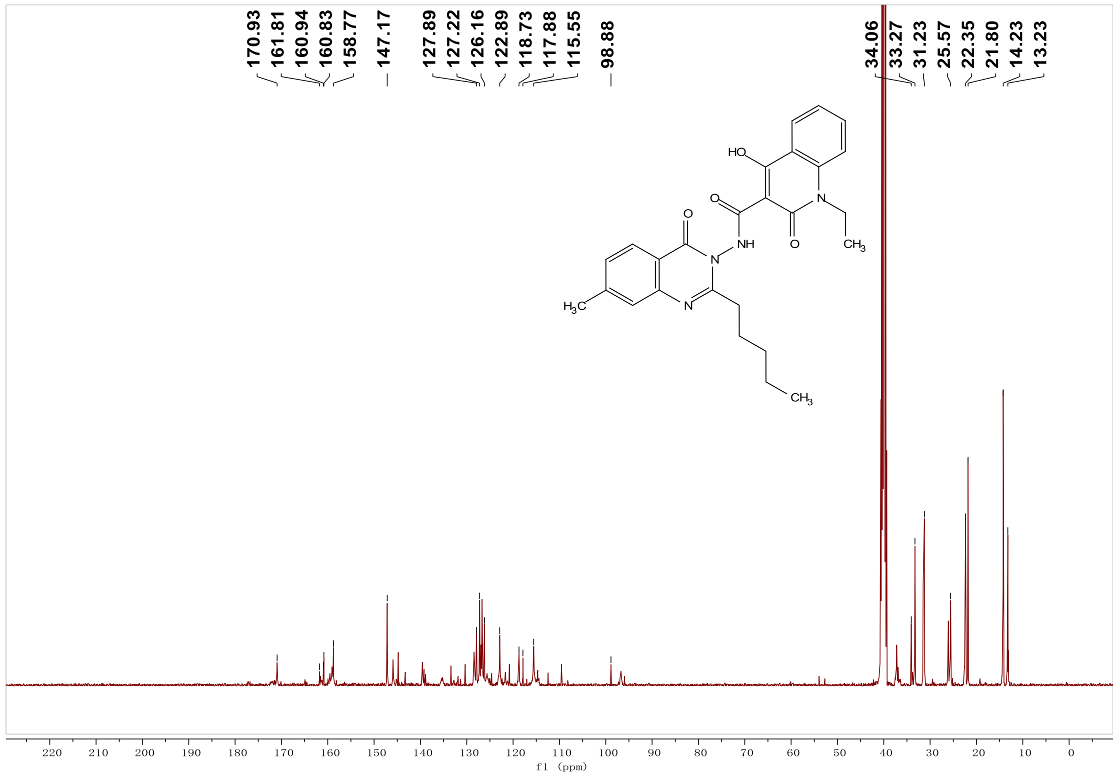

N-(7-methoxy-4-oxo-2-pentylquinazolin-3(4H)-yl)-1-ethyl-4-hydroxy-2-oxo-1,2-dihydroquinoline-3-carboxamide ***(f8)***

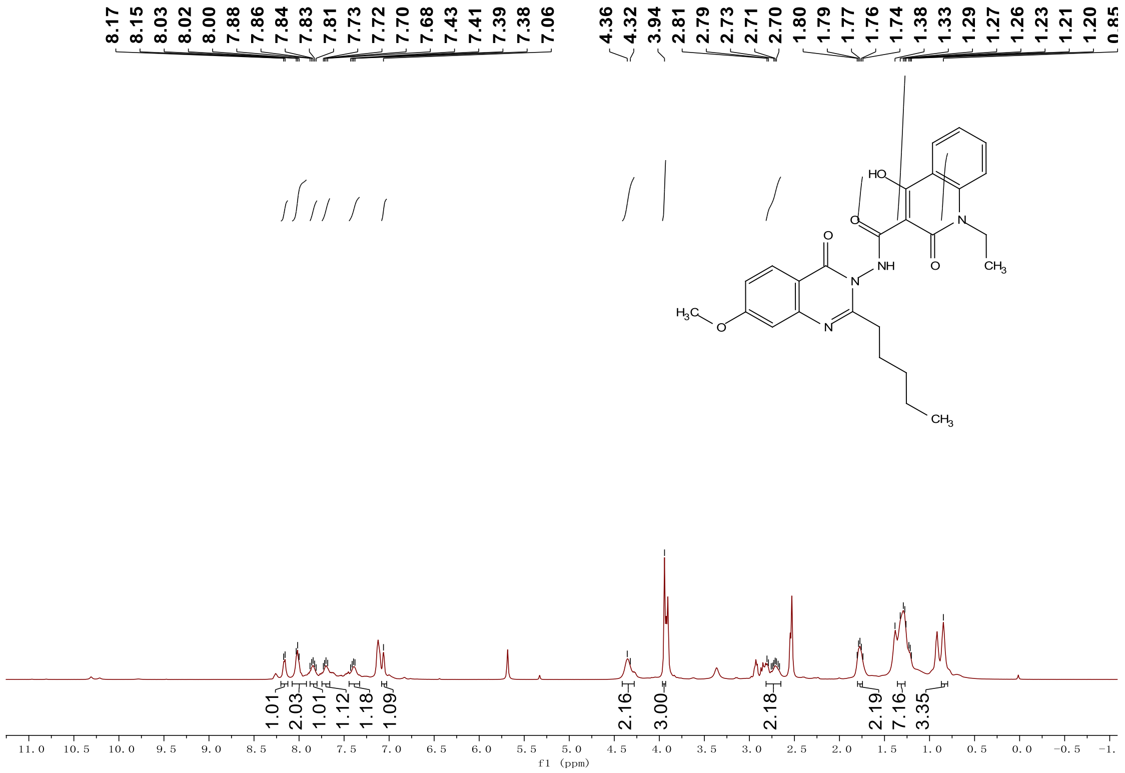

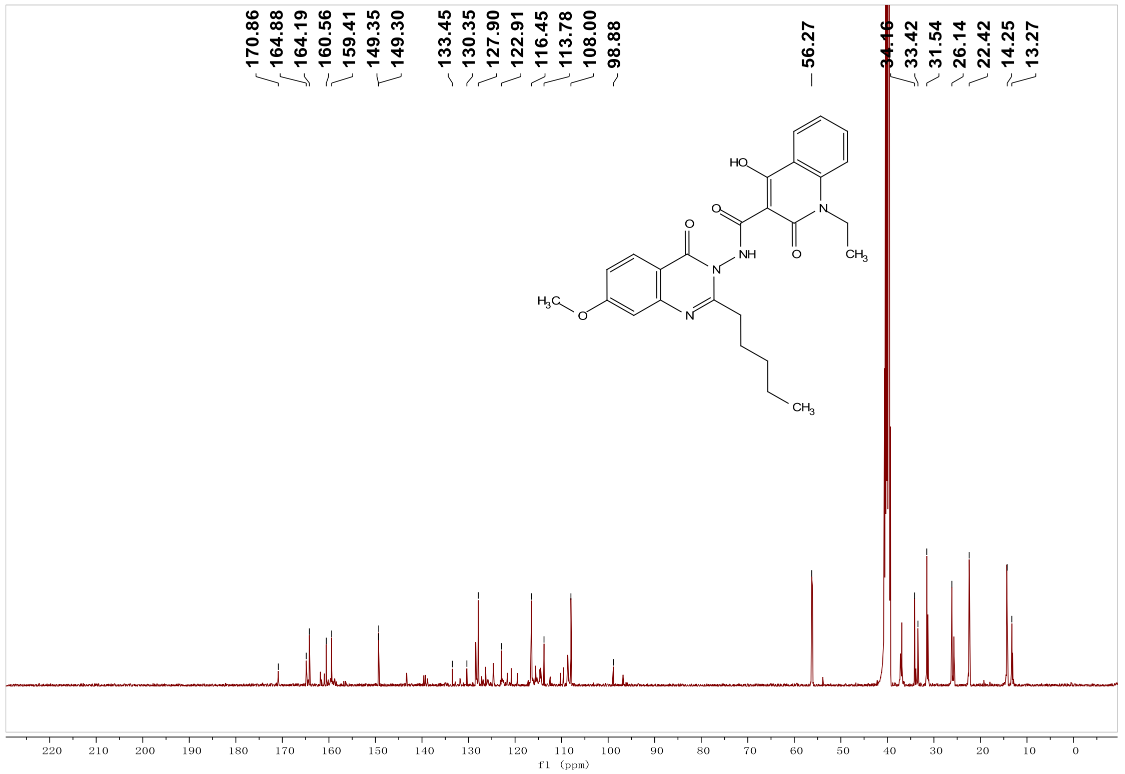

N-(6,7-dimethoxy-4-oxo-2-pentylquinazolin-3(4H)-yl)-1-ethyl-4-hydroxy-2-oxo-1,2-dihydroquinoline-3-carboxamide ***(f9)***

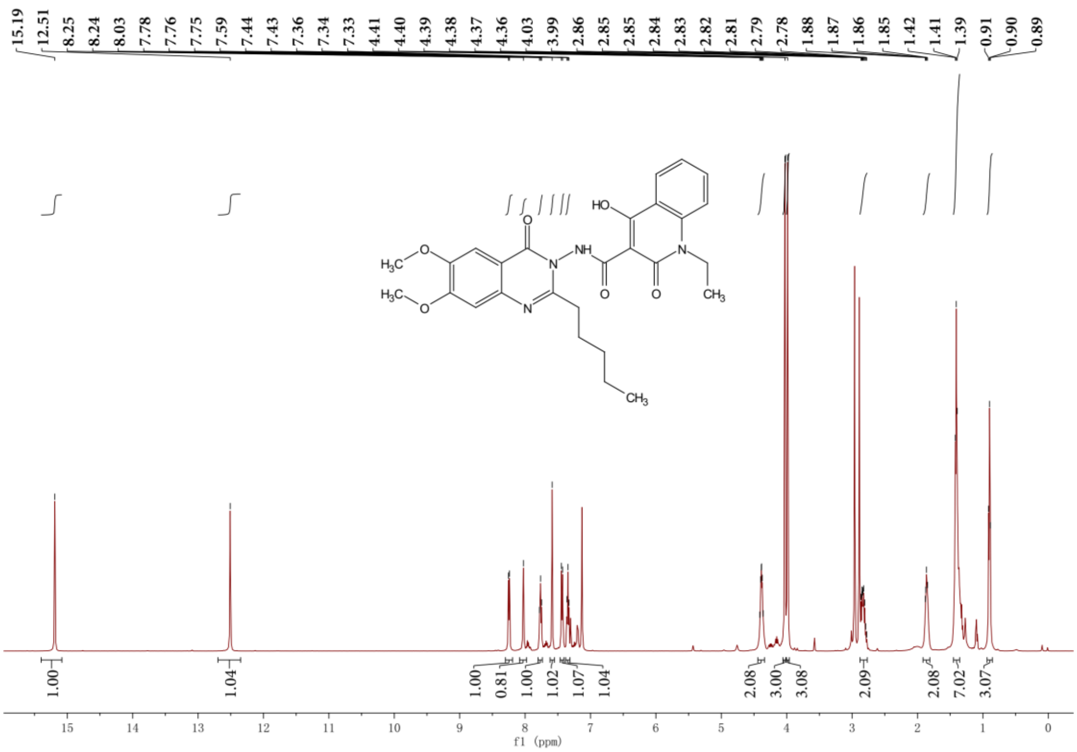

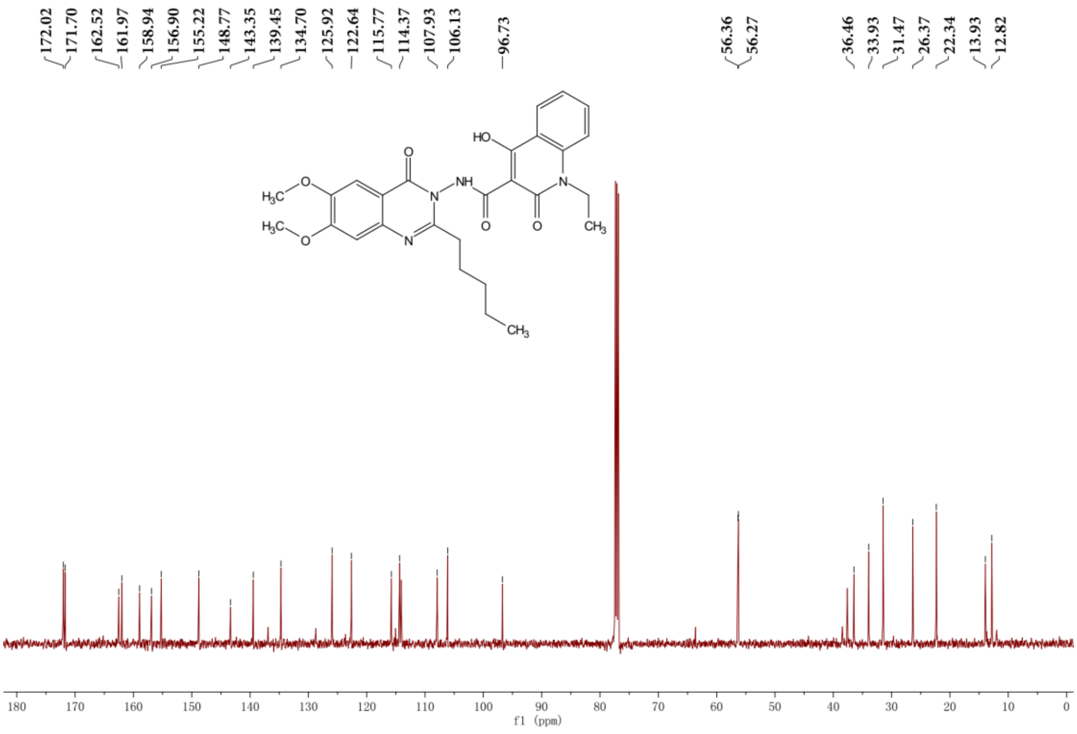

N-(8-chloro-4-oxo-2-pentylquinazolin-3(4H)-yl)-1-ethyl-4-hydroxy-2-oxo-1,2-dihydro quinoline-3-carboxamide ***(f10)***

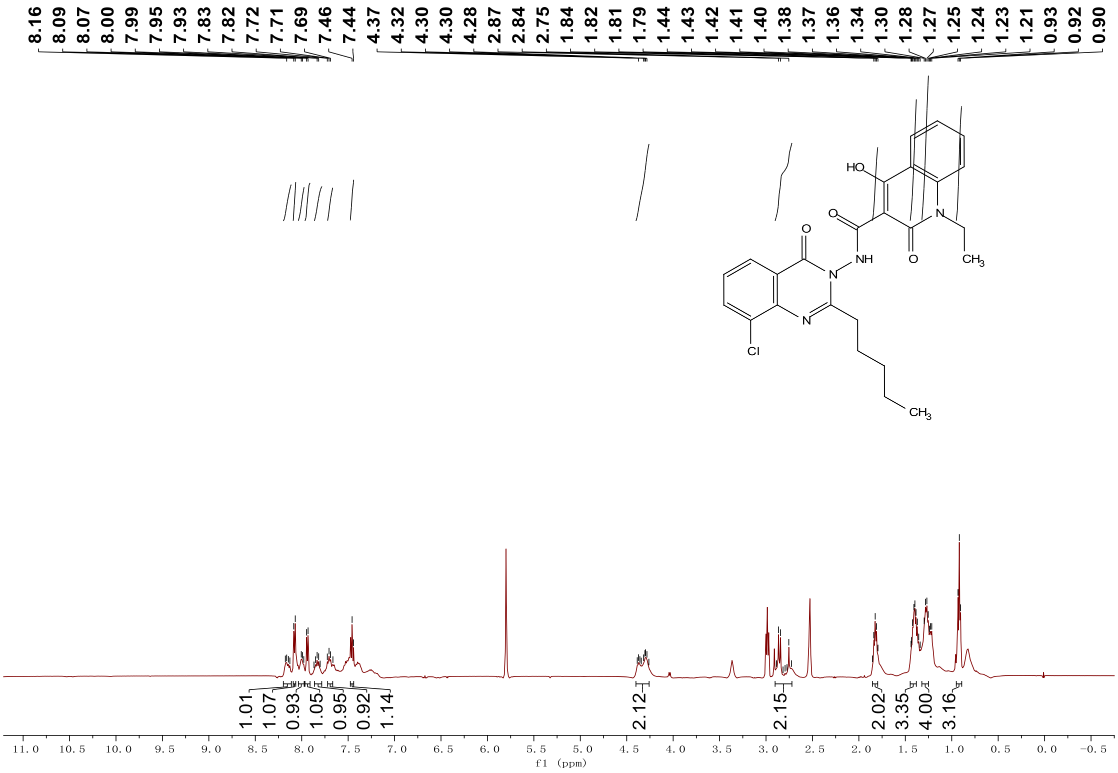

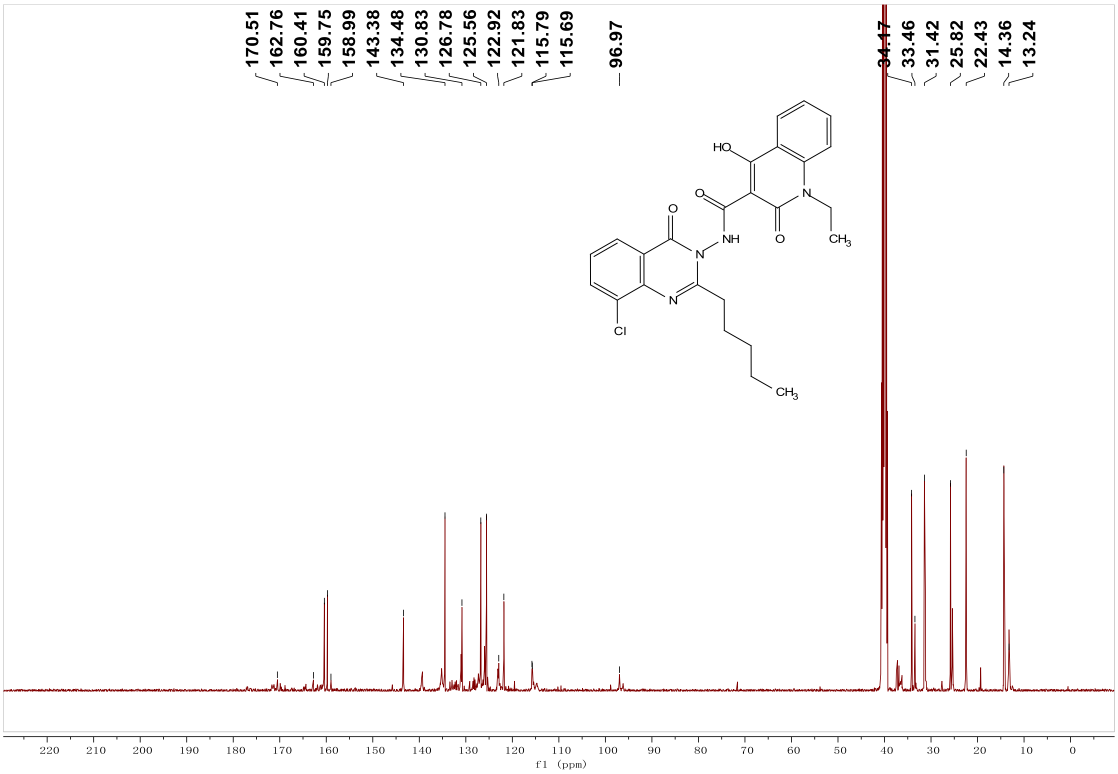

N-(8-methyl-4-oxo-2-pentylquinazolin-3(4H)-yl)-1-ethyl-4-hydroxy-2-oxo-1,2-dihydro quinoline-3-carboxamide ***(f11)***

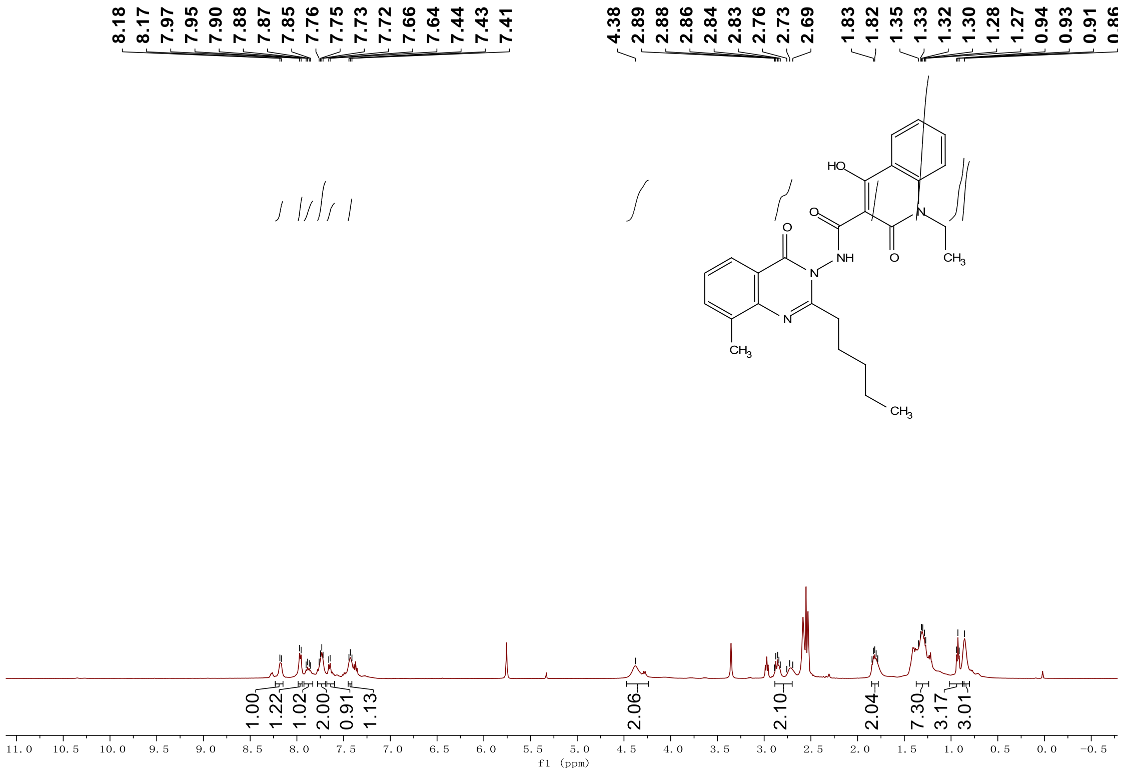

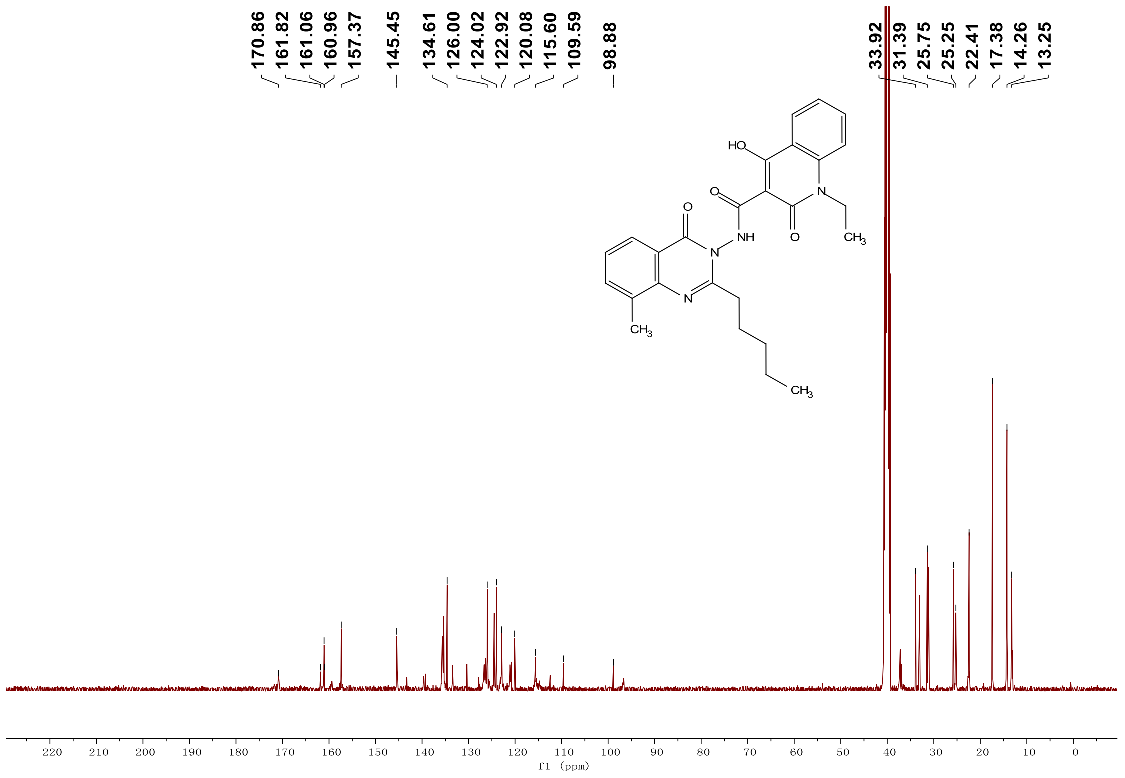

N-(8-methoxy-4-oxo-2-pentylquinazolin-3(4H)-yl)-1-ethyl-4-hydroxy-2-oxo-1,2-dihydroquinoline-3-carboxamide ***(f12)***

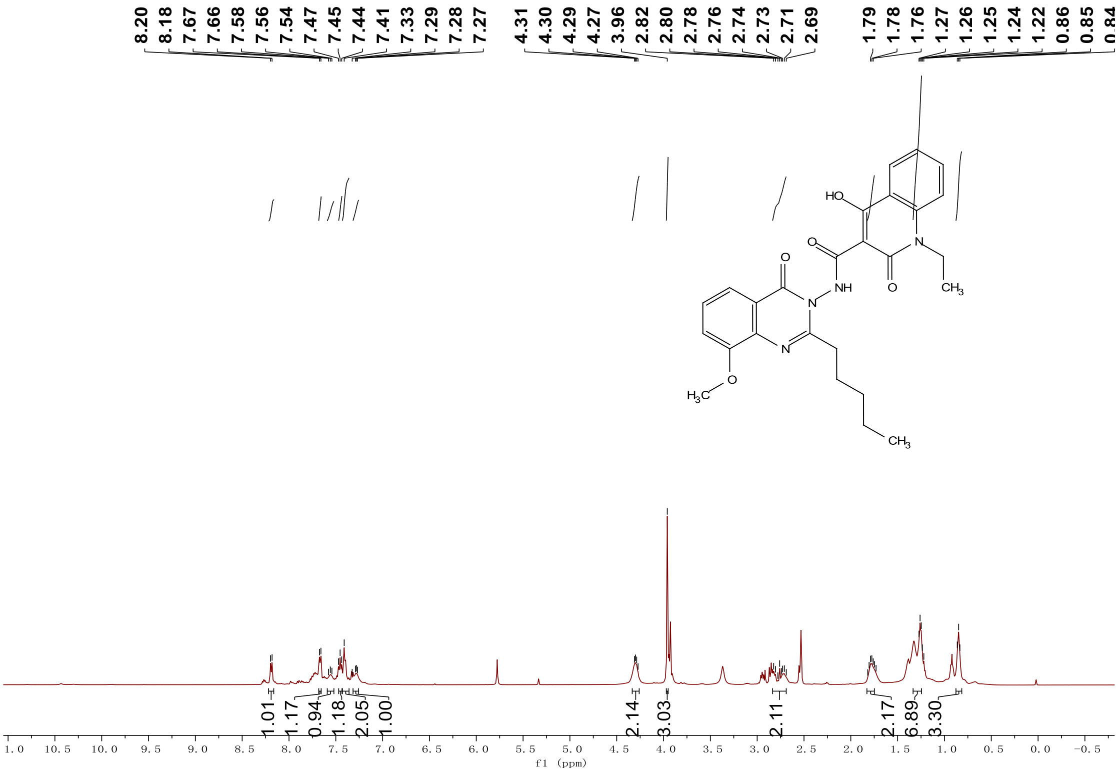

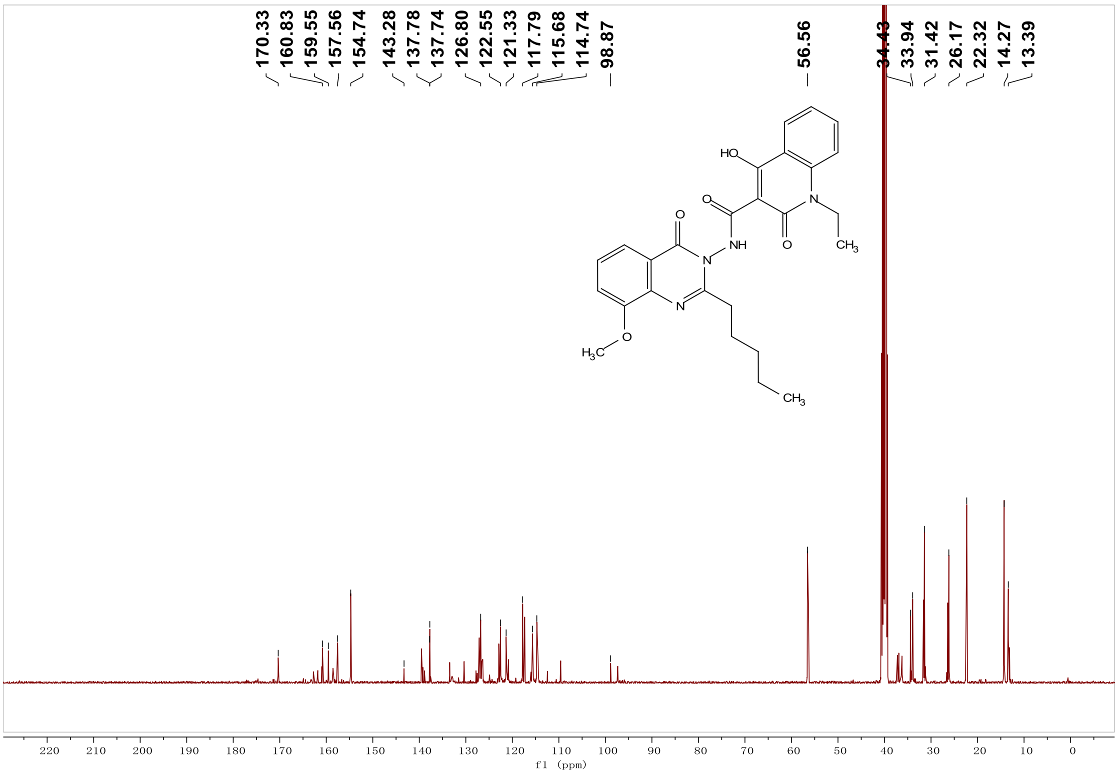

N-(2-methyl-4-oxoquinazolin-3(4H)-yl)-1-ethyl-4-hydroxy-2-oxo-1,2-dihydroquinoline-3-carboxamide ***(f13)***

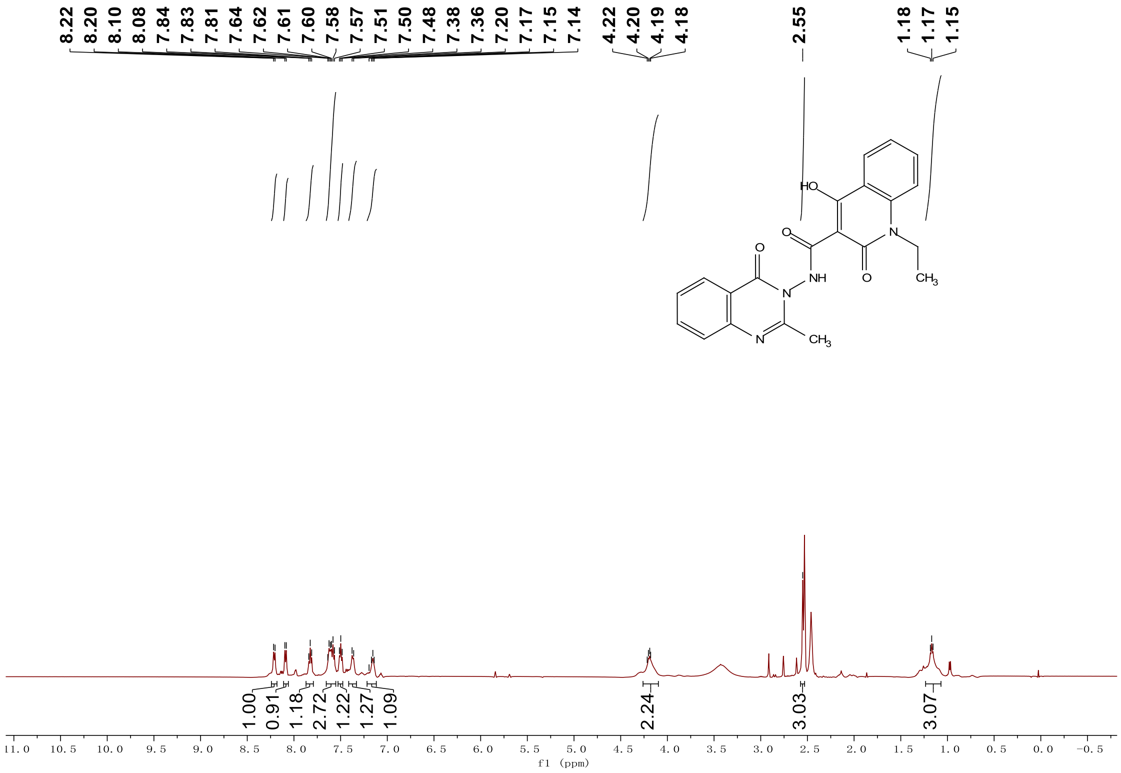

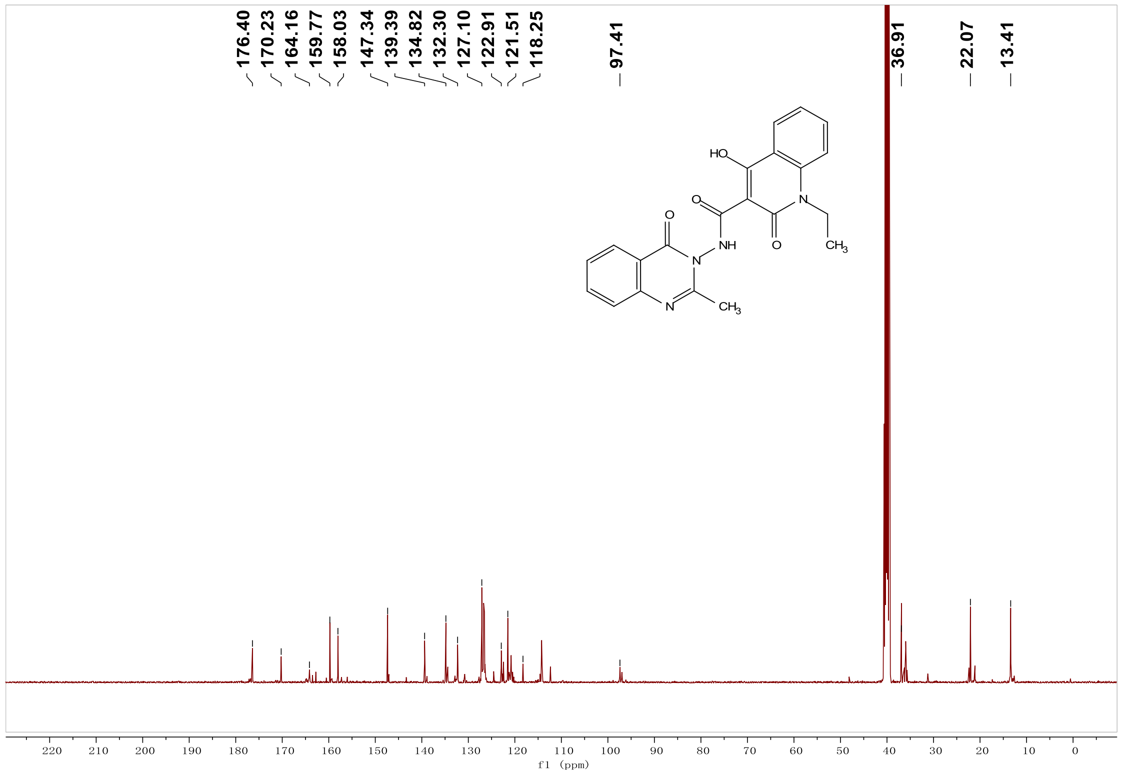

N-(2-ethyl-4-oxoquinazolin-3(4H)-yl)-1-ethyl-4-hydroxy-2-oxo-1,2-dihydroquinoline-3-carboxamide ***(f14)***

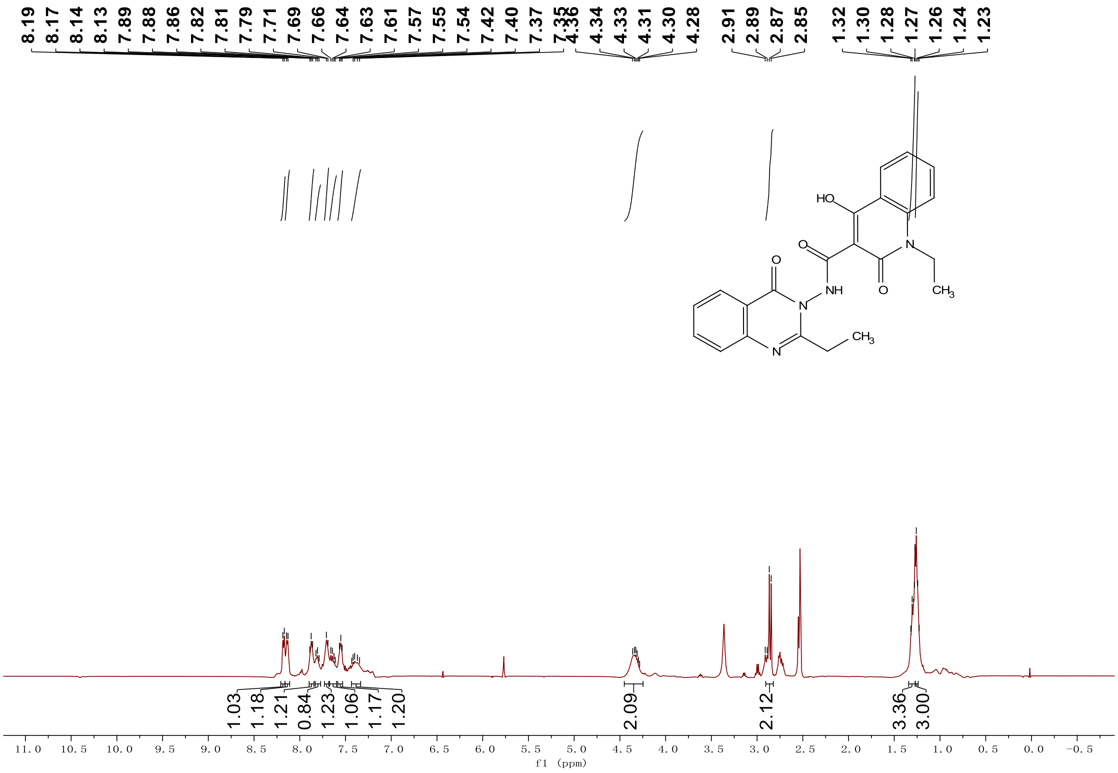

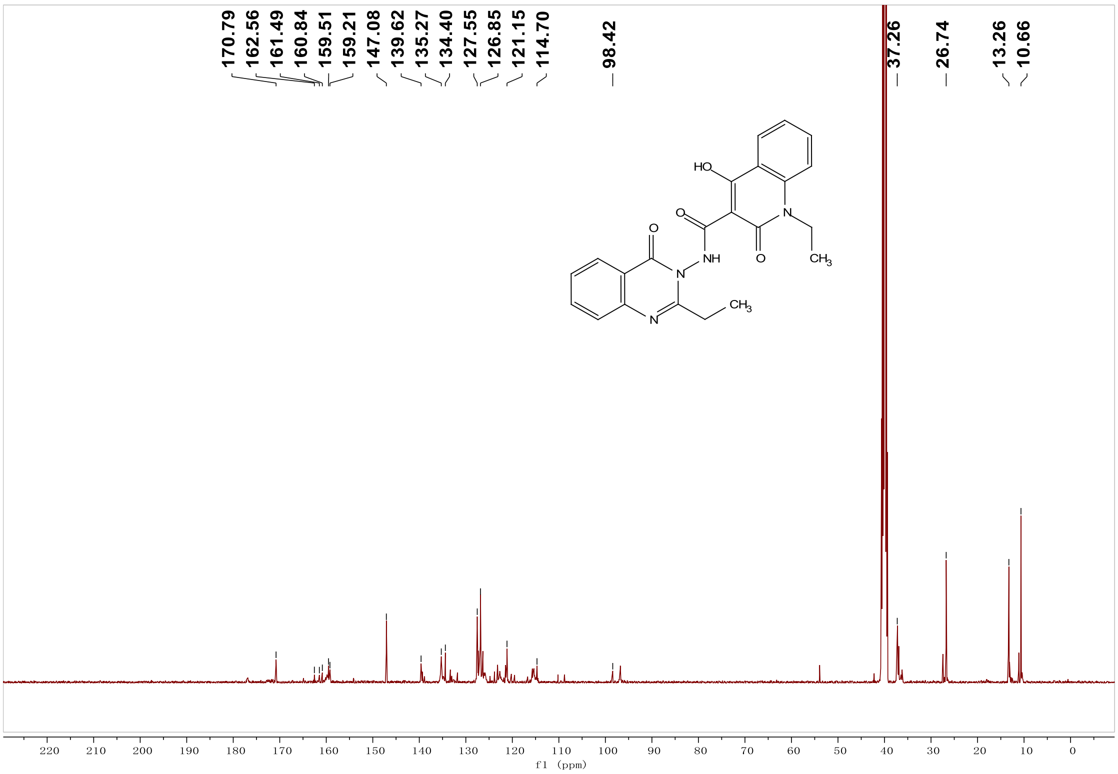

N-(4-oxo-2-propylquinazolin-3(4H)-yl)-1-ethyl-4-hydroxy-2-oxo-1,2-dihydroquinoline -3-carboxamide ***(f15)***

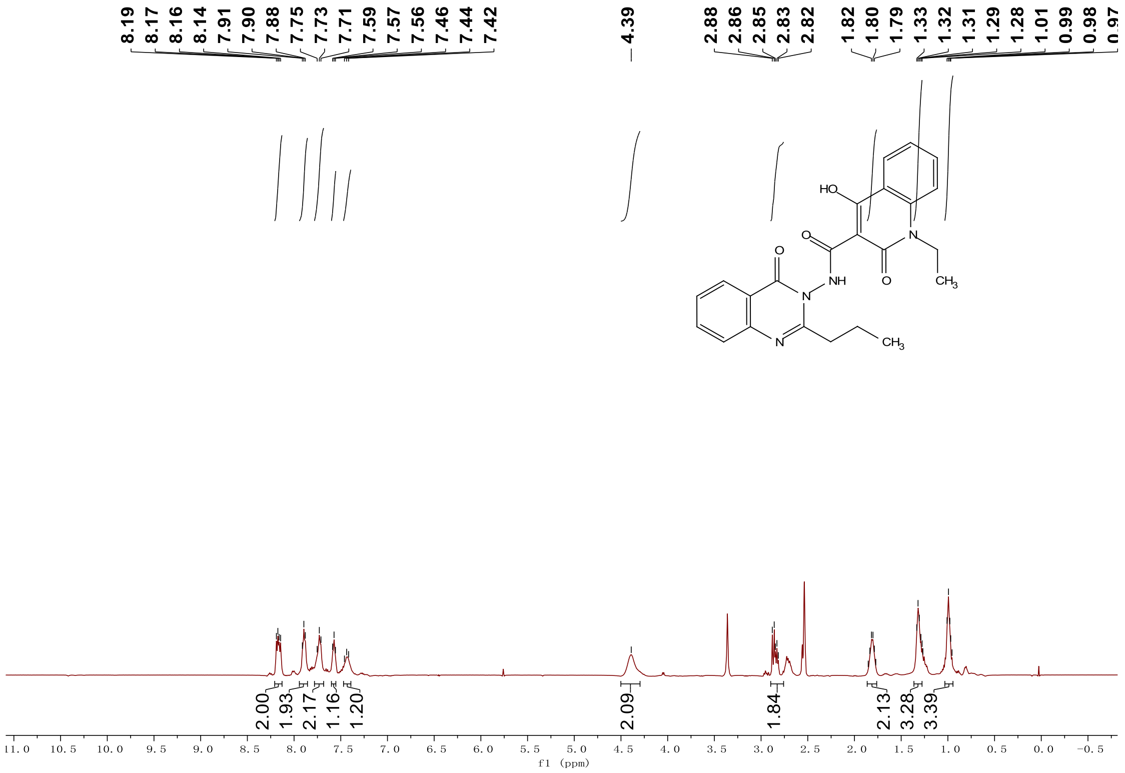

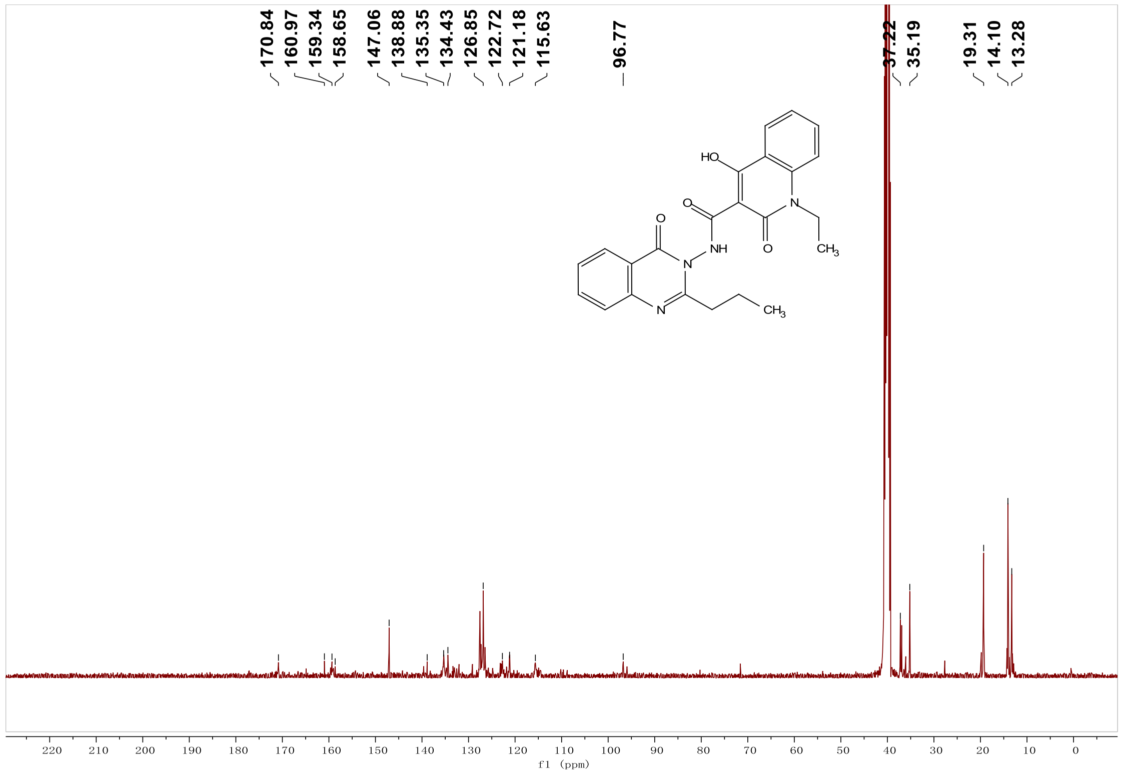

N-(2-butyl-4-oxoquinazolin-3(4H)-yl)-1-ethyl-4-hydroxy-2-oxo-1,2-dihydroquinoline-3-carboxamide ***(f16)***

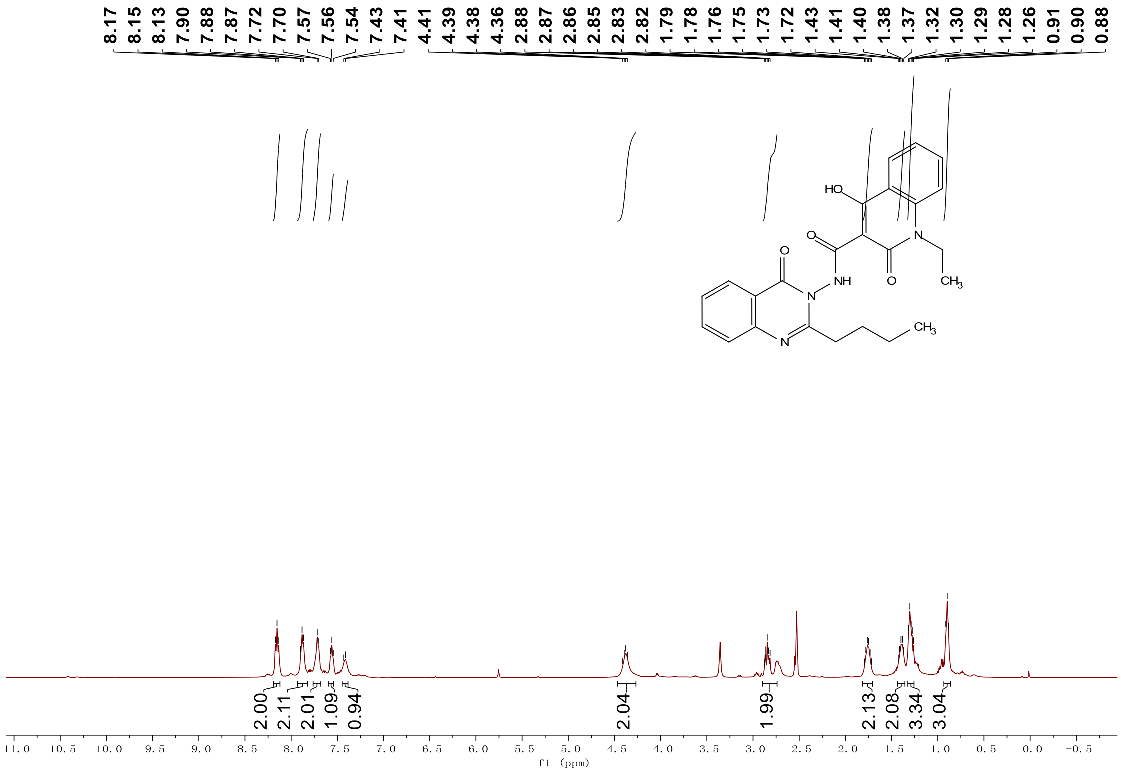

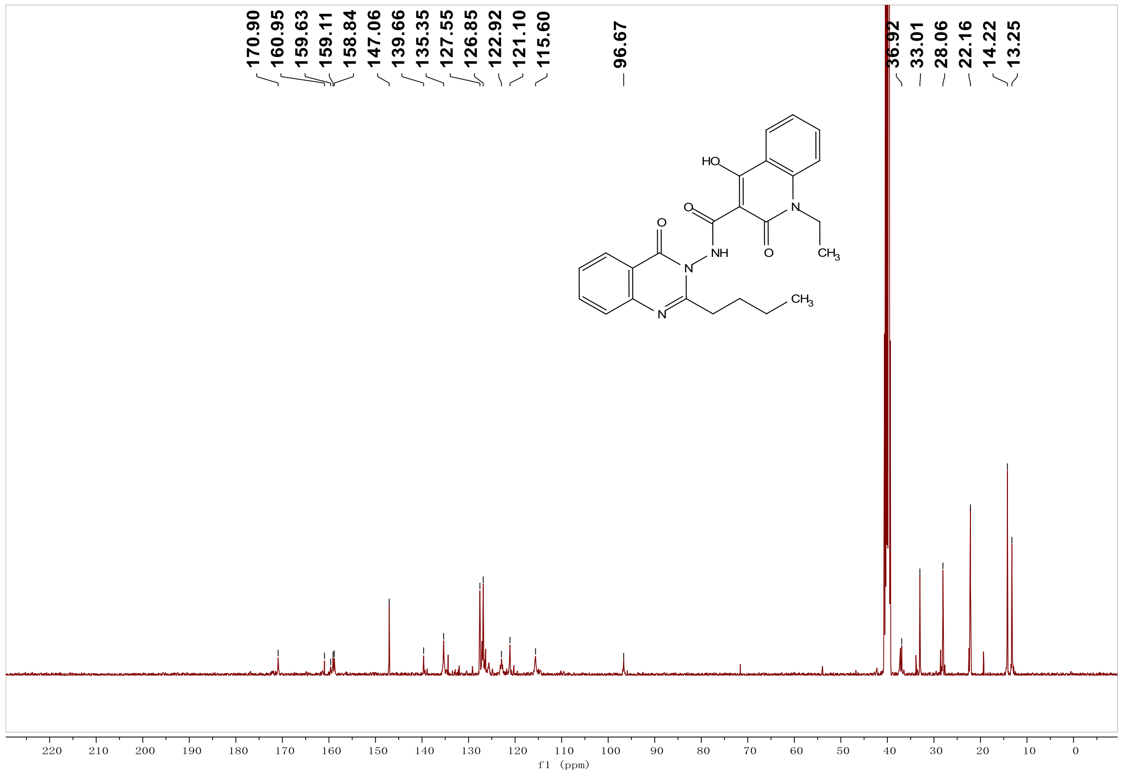

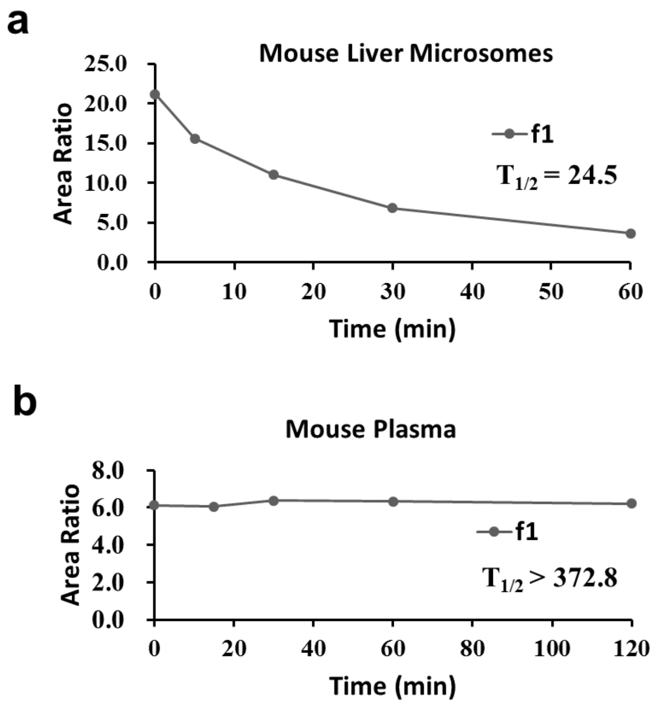

Figure S2. *In vitro* metabolic stability of compound **f1**. (a) Peak area ratio as a function of incubation time in liver microsomes. The half-life ($t_{1/2}$) of compound **f1** was estimated as 24.5 min. (b) peak area ratio as a function of incubation time in mouse plasma. The half-life ($t_{1/2}$) of compound **f1** was determined as >372.8 min.

FAME 3 Output

Visualization:

To alternate between atoms and atom numbers, move the mouse cursor over the figure.

Model: P1+P2 (depth: 5)

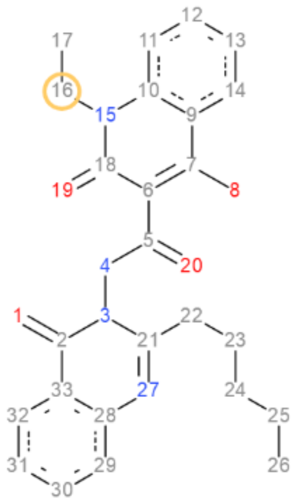

Molecule mol_1

Atom	Probability	FAMEscore
**C.16**	**0.588**	**0.539**
C.12	0.391	0.583
C.25	0.316	0.463
C.26	0.26	0.488
C.13	0.252	0.553
C.24	0.228	0.475
C.17	0.203	0.57
C.11	0.196	0.563
C.30	0.135	0.521
C.2	0.128	0.607
O.8	0.128	0.505
C.23	0.12	0.517
C.22	0.116	0.574
C.29	0.108	0.529
N.4	0.08	0.62
C.31	0.076	0.558
N.27	0.076	0.607
O.1	0.06	0.558
C.18	0.044	0.503
C.7	0.044	0.518
C.5	0.044	0.586
C.21	0.04	0.579
O.20	0.036	0.586
N.15	0.036	0.564
C.6	0.036	0.532
N.3	0.028	0.66
O.19	0.016	0.525
C.14	0.012	0.597
C.33	0.008	0.703
C.28	0.004	0.694
C.9	0.004	0.56
C.32	0.0	0.694
C.10	0.0	0.579

Figure S3. The predicted metabolic stability of **f1** by FAME3. The likelihood of being metabolized by phase 1 and 2 enzymes is listed for each heavy atom.

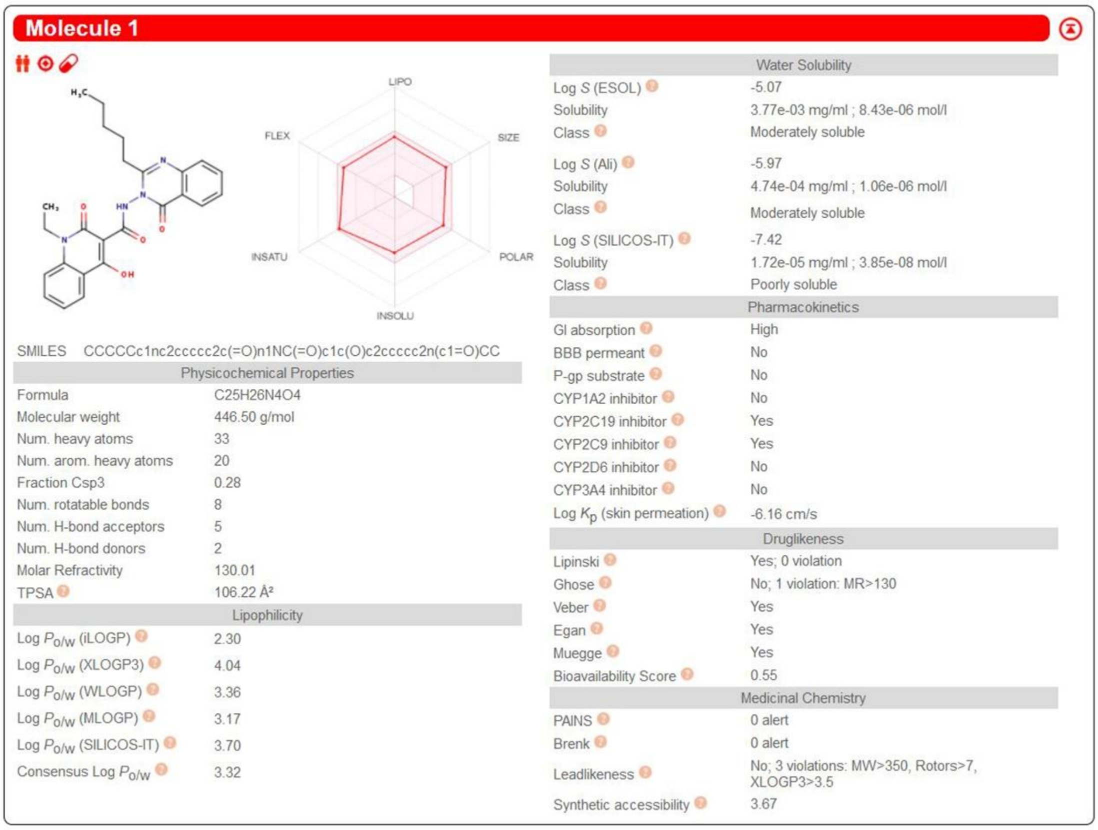

Molecule 1

SMILES CCCCCc1nc2ccccc2c(=O)n1NC(=O)c1c(O)c2ccccc2n(c1=O)CC

Physicochemical Properties	
Formula	C25H26N4O4
Molecular weight	446.50 g/mol
Num. heavy atoms	33
Num. arom. heavy atoms	20
Fraction Csp3	0.28
Num. rotatable bonds	8
Num. H-bond acceptors	5
Num. H-bond donors	2
Molar Refractivity	130.01
TPSA	106.22 Å²

Lipophilicity	
Log $P_{o/w}$ (iLOGP)	2.30
Log $P_{o/w}$ (XLOGP3)	4.04
Log $P_{o/w}$ (WLOGP)	3.36
Log $P_{o/w}$ (MLOGP)	3.17
Log $P_{o/w}$ (SILICOS-IT)	3.70
Consensus Log $P_{o/w}$	3.32

Water Solubility	
Log *S* (ESOL)	-5.07
Solubility	3.77e-03 mg/ml ; 8.43e-06 mol/l
Class	Moderately soluble
Log *S* (Ali)	-5.97
Solubility	4.74e-04 mg/ml ; 1.06e-06 mol/l
Class	Moderately soluble
Log *S* (SILICOS-IT)	-7.42
Solubility	1.72e-05 mg/ml ; 3.85e-08 mol/l
Class	Poorly soluble

Pharmacokinetics	
GI absorption	High
BBB permeant	No
P-gp substrate	No
CYP1A2 inhibitor	No
CYP2C19 inhibitor	Yes
CYP2C9 inhibitor	Yes
CYP2D6 inhibitor	No
CYP3A4 inhibitor	No
Log K_p (skin permeation)	-6.16 cm/s

Druglikeness	
Lipinski	Yes; 0 violation
Ghose	No; 1 violation: MR>130
Veber	Yes
Egan	Yes
Muegge	Yes
Bioavailability Score	0.55

Medicinal Chemistry	
PAINS	0 alert
Brenk	0 alert
Leadlikeness	No; 3 violations: MW>350, Rotors>7, XLOGP3>3.5
Synthetic accessibility	3.67

Figure S4. ADME profiles predicted for compound **f1** by SwissADME (http://www.swissadme.ch/).

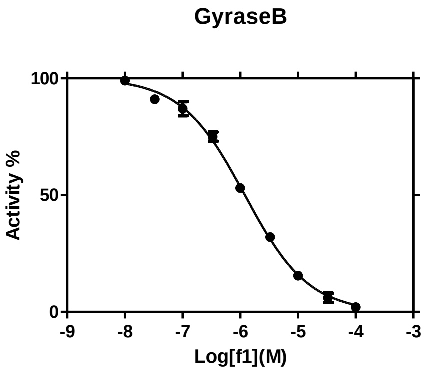
GyraseB
Activity %
100
50
0
-9
-8
-7
-6
-5
-4
-3
Log[f1](M)

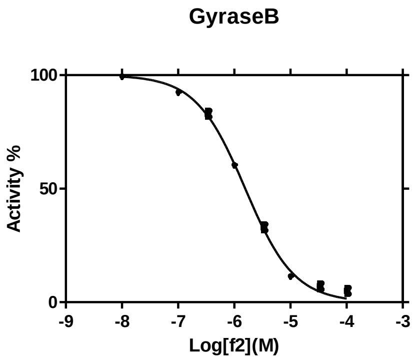
GyraseB
Activity %
100
50
0
-9
-8
-7
-6
-5
-4
-3
Log[f2](M)

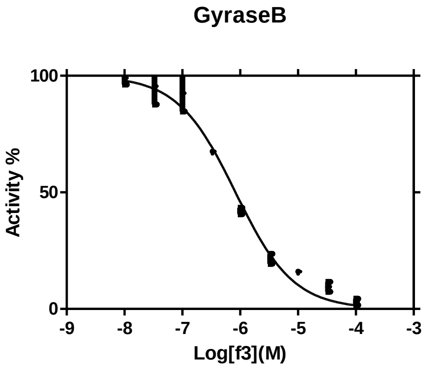
GyraseB
Activity %
100
50
0
-9
-8
-7
-6
-5
-4
-3
Log[f3](M)

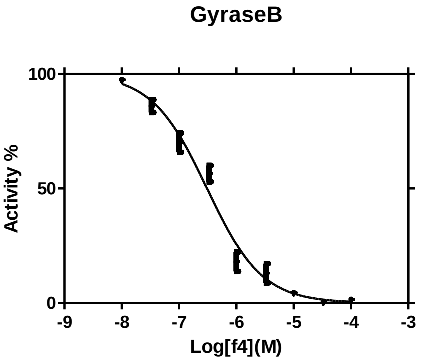
GyraseB
Activity %
100
50
0
-9
-8
-7
-6
-5
-4
-3
Log[f4](M)

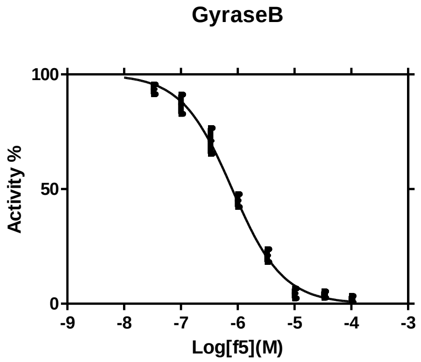
GyraseB
Activity %
100
50
0
-9
-8
-7
-6
-5
-4
-3
Log[f5](M)

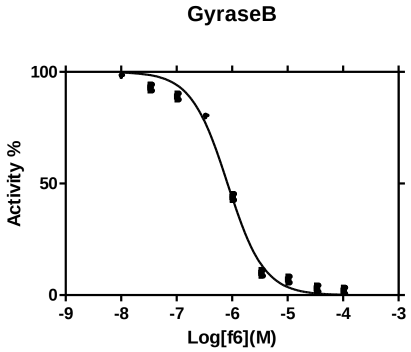
GyraseB
Activity %
100
50
0
-9
-8
-7
-6
-5
-4
-3
Log[f6](M)

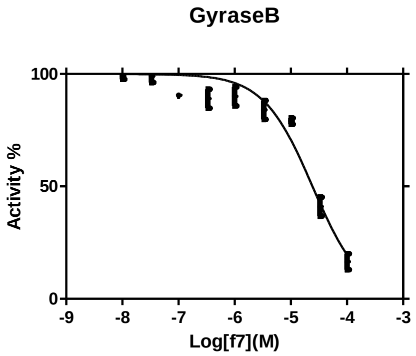
GyraseB
Activity %
100
50
0
-9
-8
-7
-6
-5
-4
-3
Log[f7](M)

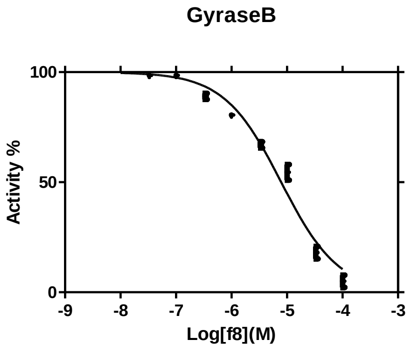
GyraseB
Activity %
100
50
0
-9
-8
-7
-6
-5
-4
-3
Log[f8](M)

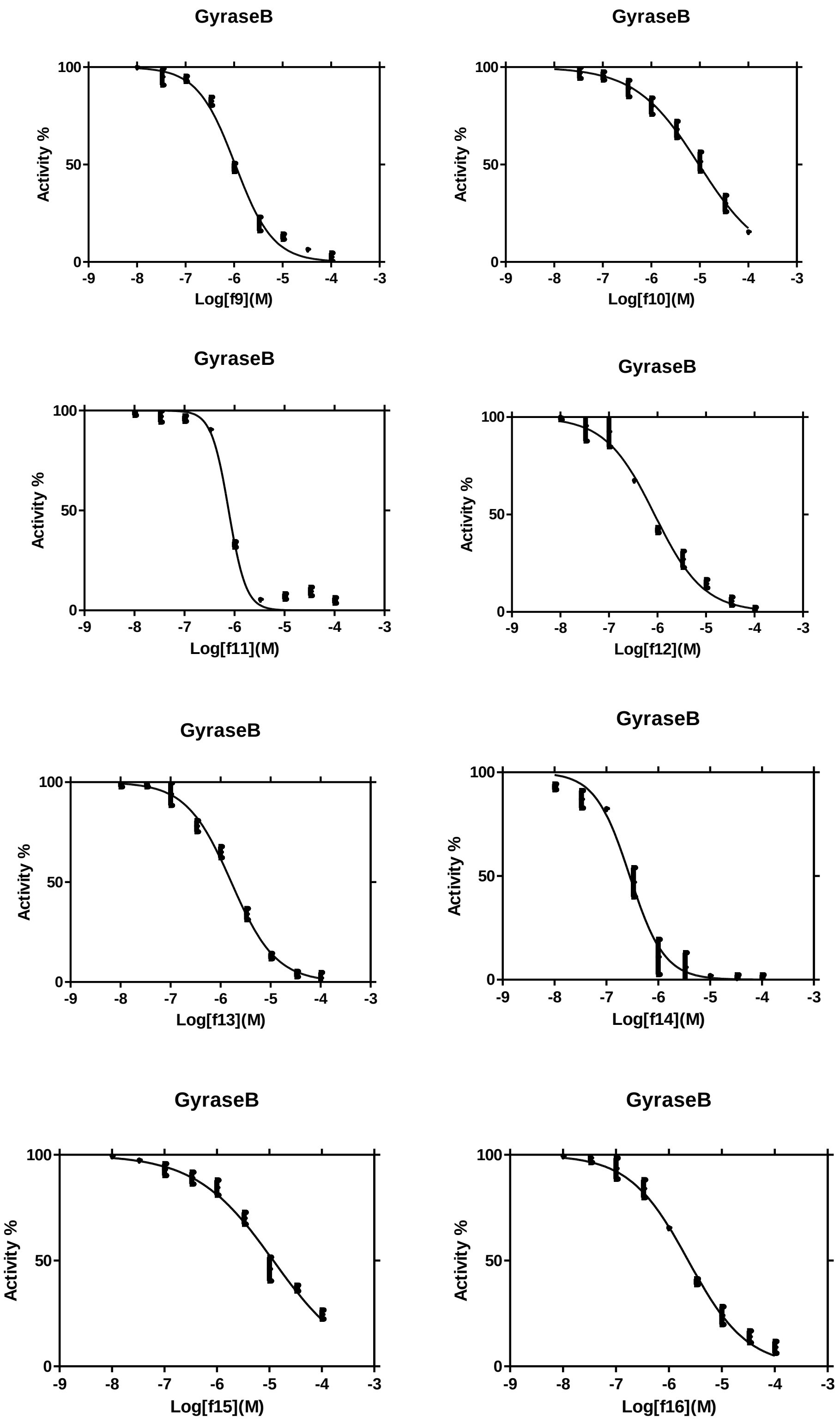

Figure S5. The dose-response curves for all the synthesized *N*-(4-oxoquinazolin-3(4*H*)-yl)-4-hydroxy-2-quinolone-3-carboxamides.

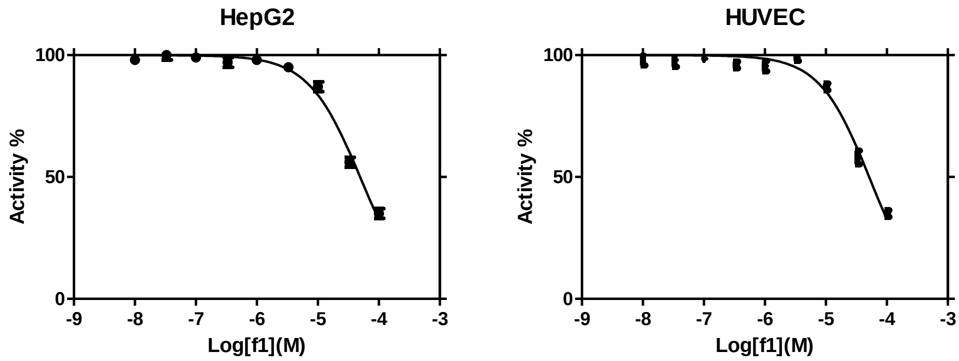

Figure S6. Dose-response curves of compound **f1** (AG-690/11765367) for its *in vitro* toxicity to HepG2 and HUVEC.

Figure S7. HPLC purity data of all the target compounds f1-f16.

f1

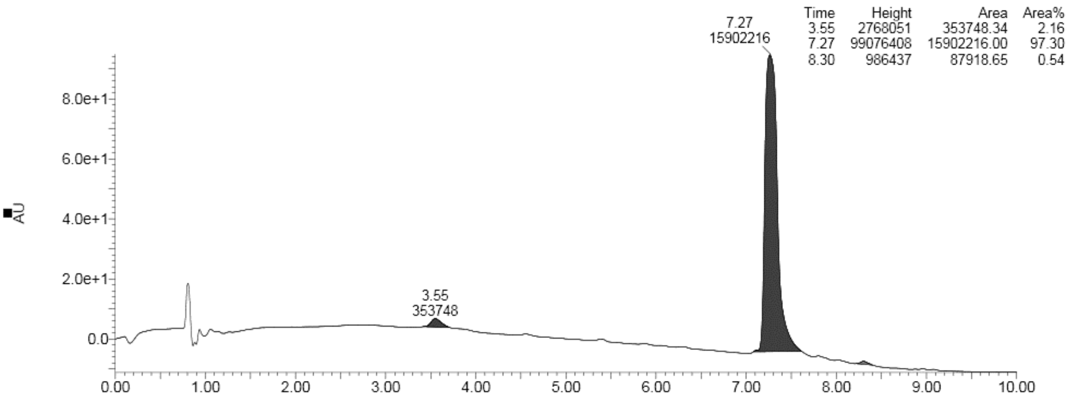

Peak	Retention Time (min)	Area (AU*s)	Area (%)
1	3.55	353748.34	2.16
2	7.27	15902216	97.30
3	8.30	87918.65	0.54

f2

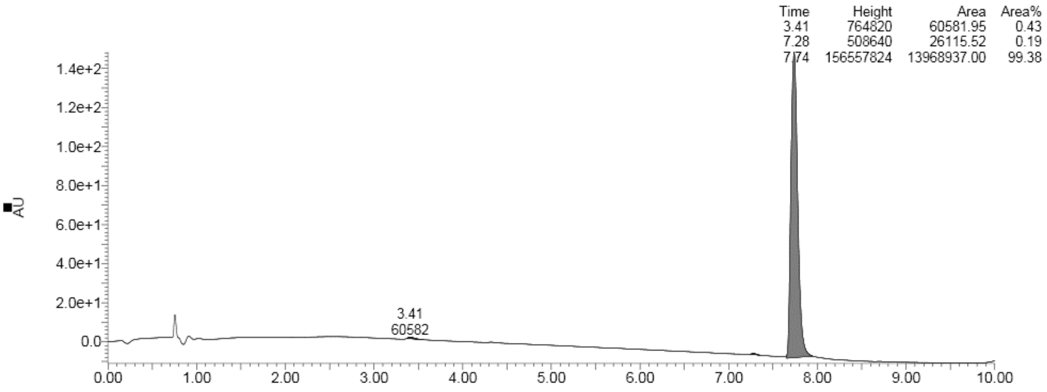

Peak	Retention Time (min)	Area (AU*s)	Area (%)
1	3.41	60581.95	0.43
2	7.28	26115.52	0.19
3	7.74	13968937.00	99.38

f3

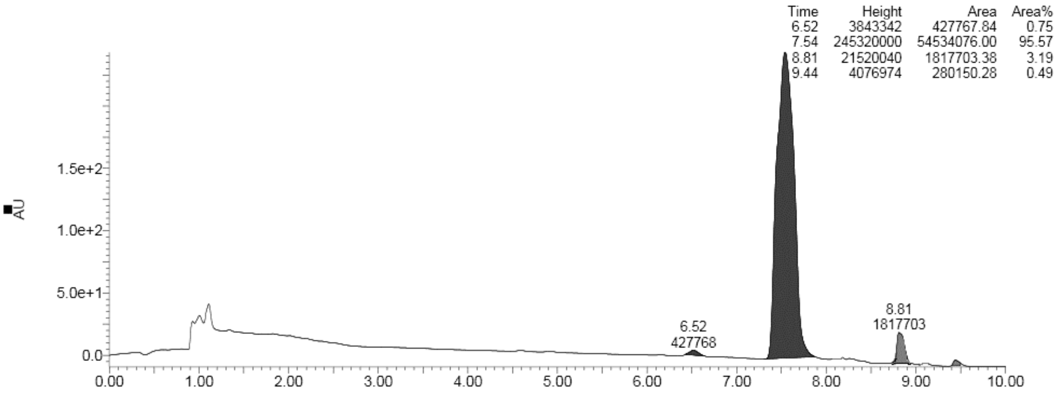

Peak	Retention Time (min)	Area (AU*s)	Area (%)
1	6.52	427767.84	0.75
2	7.54	54534076.00	95.57
3	8.81	1817703.38	3.19
4	9.44	280150.28	0.49

f4

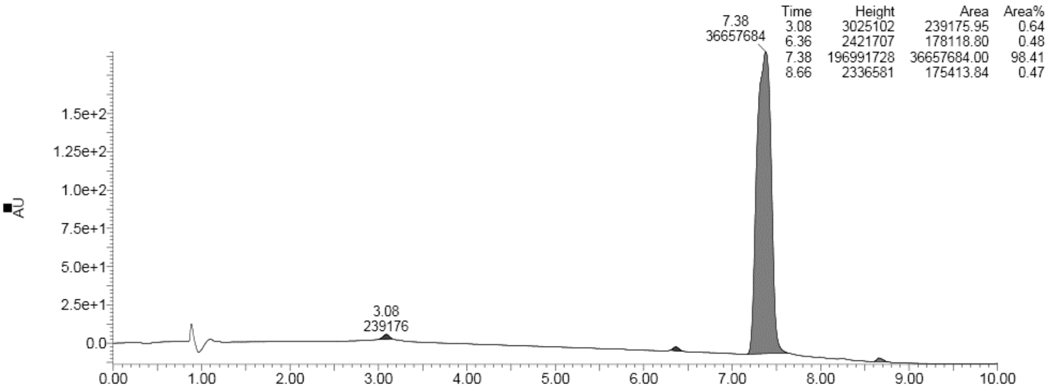

Peak	Retention Time (min)	Area (AU*s)	Area (%)
1	3.08	23917.95	0.64
2	6.36	178118.80	0.48
3	7.38	36657684.00	98.41
4	8.66	175413.84	0.47

f5

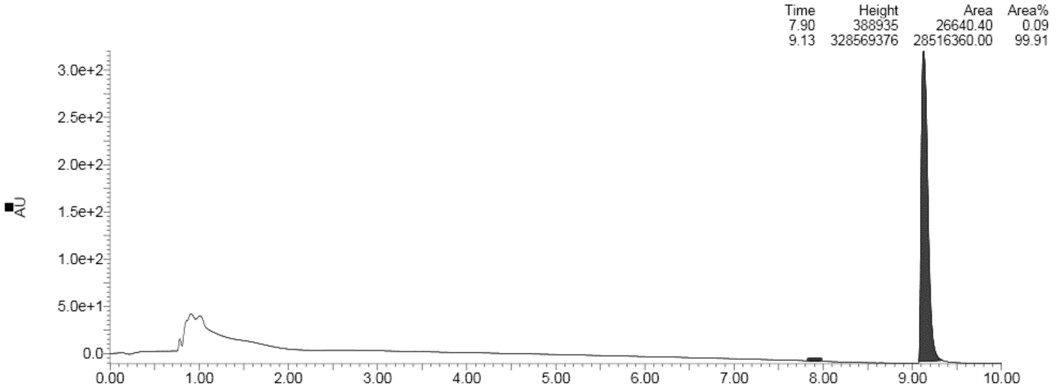

Peak	Retention Time (min)	Area (AU*s)	Area (%)
1	7.90	26640.40	0.09
2	9.13	28516360.00	99.91

f6

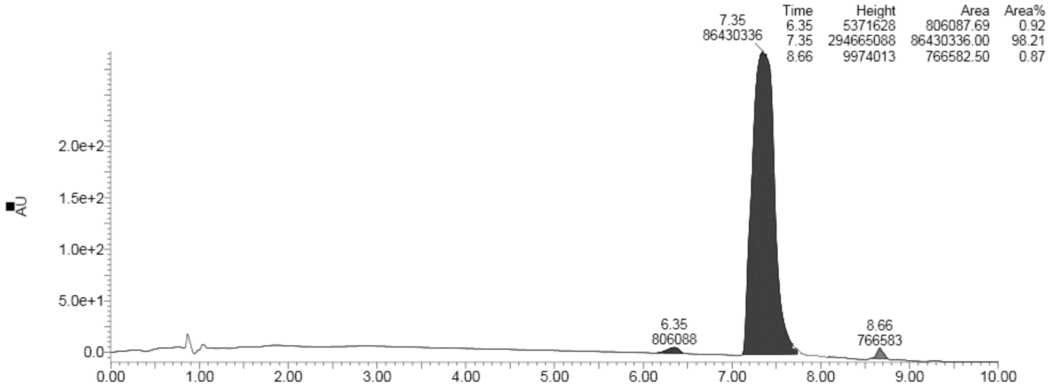

Peak	Retention Time (min)	Area (AU*s)	Area (%)
1	6.35	806087.69	0.92
2	7.35	86430336.00	98.21
3	8.66	766582.50	0.87

f7

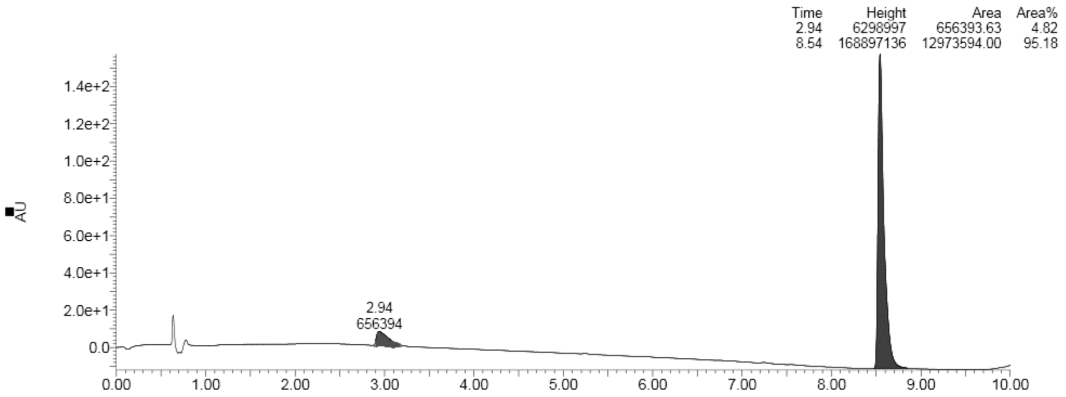

Peak	Retention Time (min)	Area (AU*s)	Area (%)
1	2.94	656393.63	4.82
2	8.54	12973594.00	95.18

f8

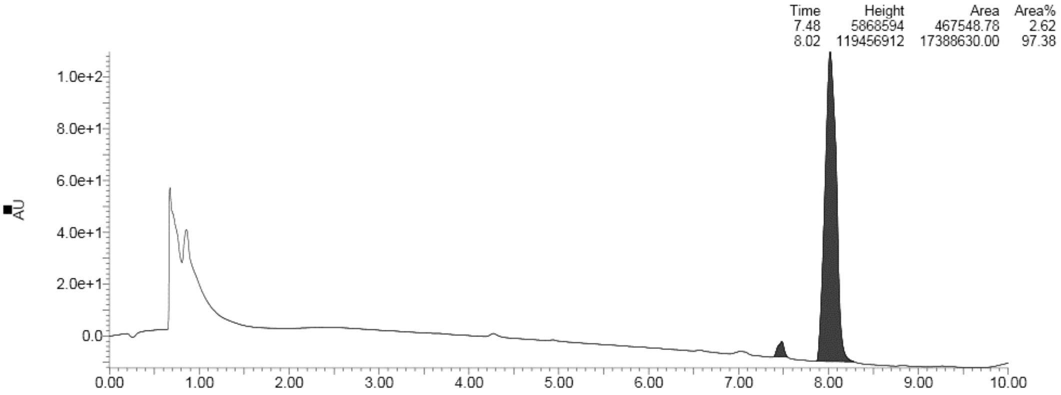

Peak	Retention Time (min)	Area (AU*s)	Area (%)
1	7.48	467548.78	2.62
2	8.02	17388630.00	97.38

f9

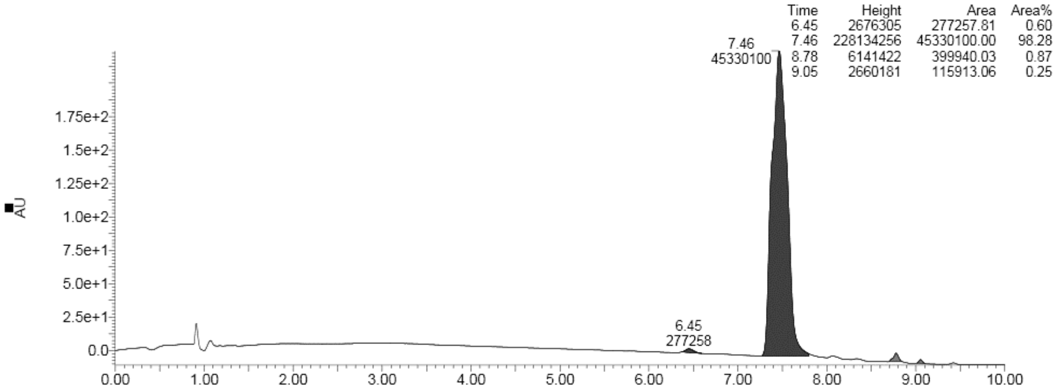

Peak	Retention Time (min)	Area (AU*s)	Area (%)
1	6.45	277257.81	0.60
2	7.46	45330100.00	98.28
3	8.78	399940.03	0.87
4	9.05	115913.06	0.25

f10

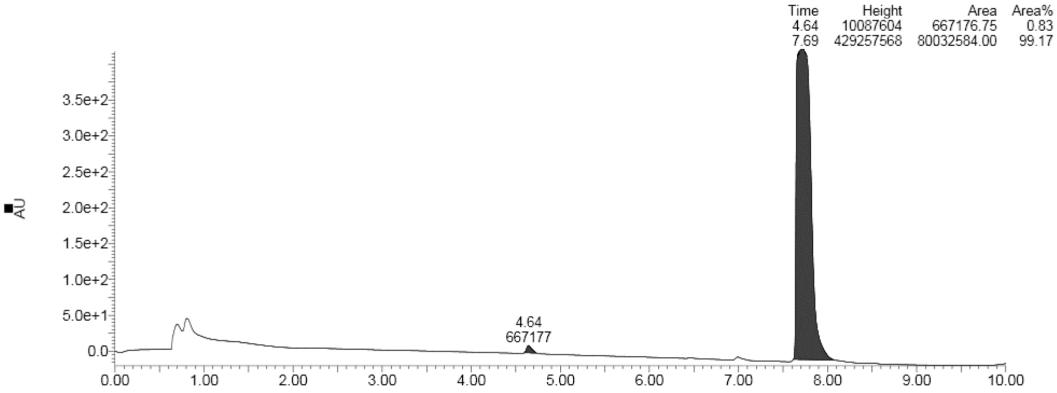

Peak	Retention Time (min)	Area (AU*s)	Area (%)
1	4.64	667176.75	0.83
2	7.69	80032584.00	99.17

f11

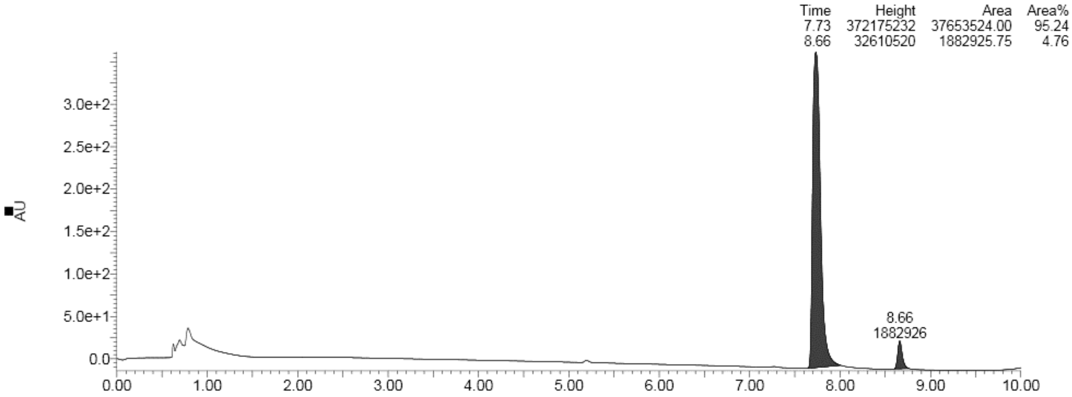

Peak	Retention Time (min)	Area (AU*s)	Area (%)
1	7.73	37653254.00	95.24
2	8.66	1882925.75	4.76

f12

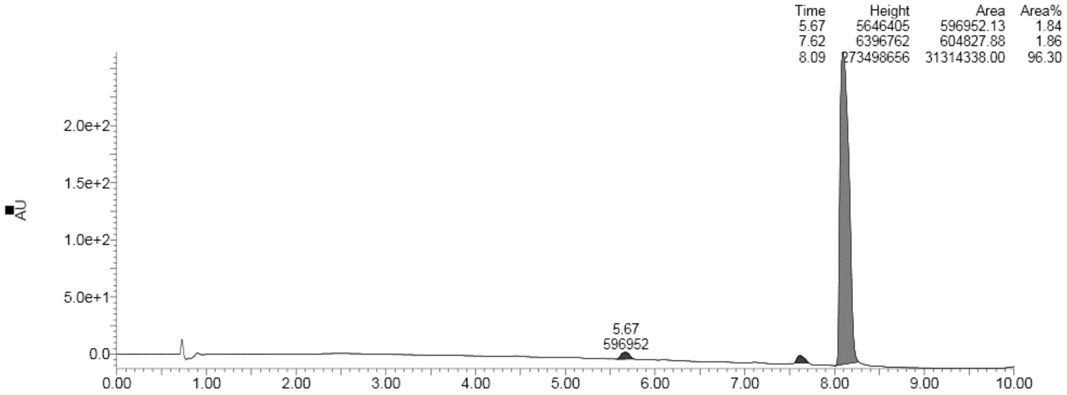

Peak	Retention Time (min)	Area (AU*s)	Area (%)
1	5.67	596952.13	1.84
2	7.62	604827.88	1.86
3	8.09	31314338.00	96.30

f13

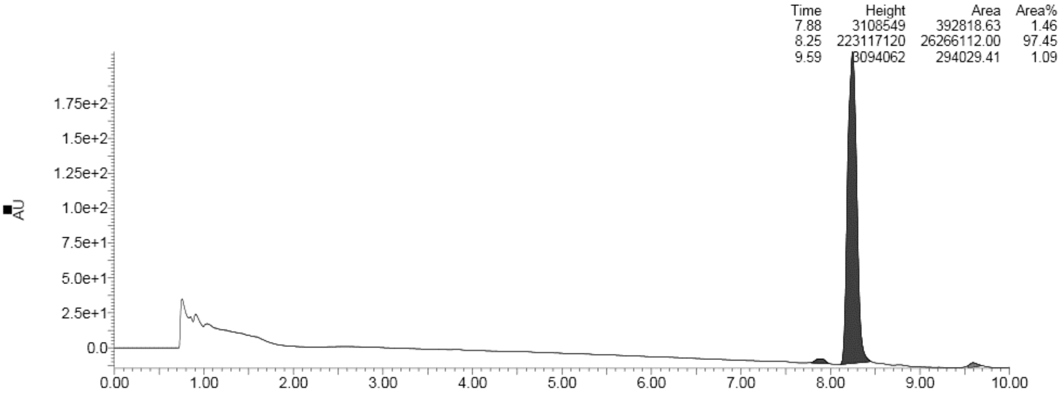

Peak	Retention Time (min)	Area (AU*s)	Area (%)
1	7.88	392818.63	1.46
2	8.25	26266112.00	97.45
3	9.59	294029.41	1.09

f14

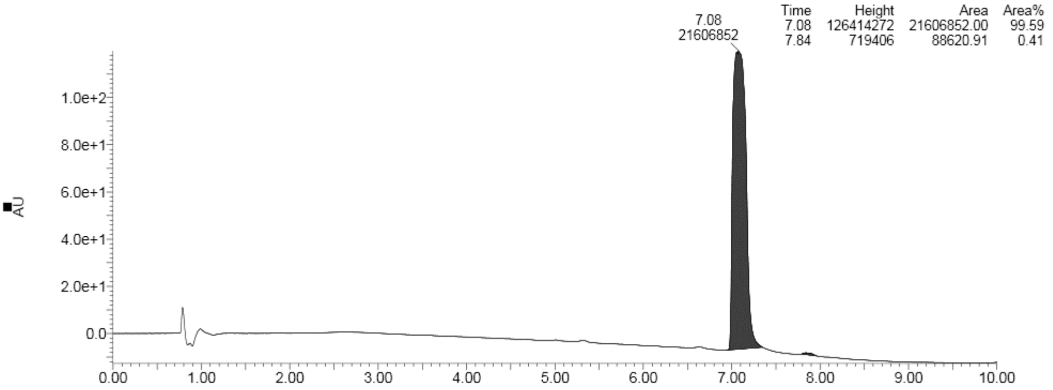

Peak	Retention Time (min)	Area (AU*s)	Area (%)
1	7.08	21606852.00	99.59
2	7.84	88620.91	0.41

f15

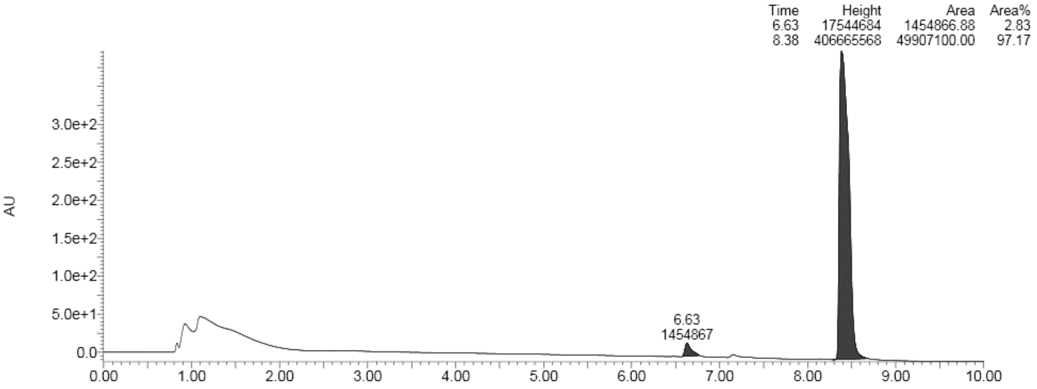

Peak	Retention Time (min)	Area (AU*s)	Area (%)
1	6.63	1454866.88	2.83
2	8.38	49907100.00	97.17

f16

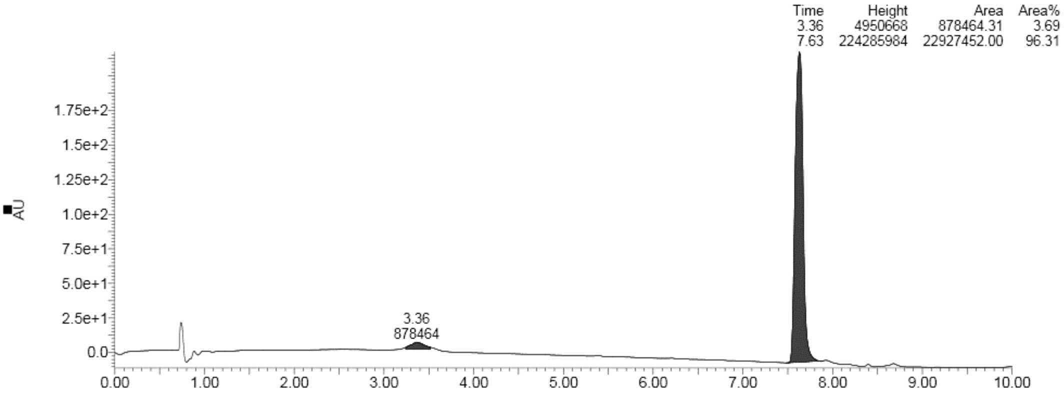

Peak	Retention Time (min)	Area (AU*s)	Area (%)
1	3.36	878464.31	3.96
2	7.63	22927452.00	96.31
